# Quantum Chemical Calculations, Topological Properties, ADME/Molecular Docking Studies, and Hirshfeld Surface Analysis on Some Organic UV-Filters

**DOI:** 10.1021/acsomega.4c10102

**Published:** 2025-04-08

**Authors:** Feride Akman, Buşra Kutlu

**Affiliations:** † Vocational School of Food, Agriculture and Livestock, Bingol University, 12000 Bingol, Turkey; ‡ Chemistry Program, Institute of Sciences, Bingol University, 12000 Bingol, Turkey

## Abstract

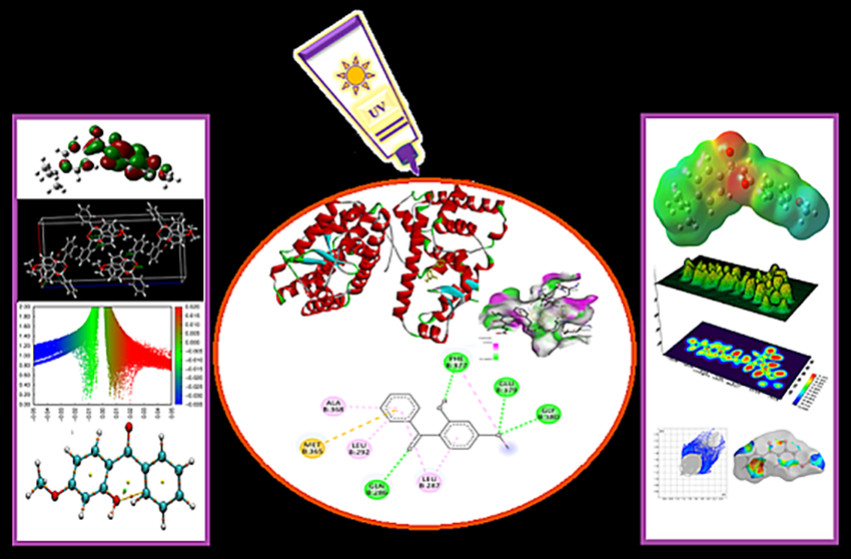

This study aimed to find a theoretical solution to the problem of photochemical instability of organic UV filters by changing the solvent environments. For this purpose, the four most important organic filters containing UV-sensitive groups, such as oxybenzone, avobenzone, octinoxate, and padimate O, were first selected, and the theoretically optimized geometries were determined by the density functional theory (DFT) method using the B3LYP/6-31G(d,p) basis set. Frontier molecular orbitals (FMOs) and molecular electrostatic potential (MEP) analyses were conducted to reveal differences in the reactivities of the molecules. The oscillator strengths, absorption wavelengths, and excitation energies in gas, water, ethanol, and *n*-hexane phases were determined with the help of the conductor-like polarizable continuum model (CPCM) and time-dependent density functional theory (TD-DFT) to study the effect of solvents on chemical parameters. In light of the obtained data, Natural localized molecular orbital (NLMO), atoms in molecules (AIM), and natural bond orbital (NBO) analyses were done to determine the stability and UV filtering capacity of the molecules. Additionally, topological and Fukui investigations were included. Molecular docking, ADME (absorption, delivery, metabolism, and excretion) properties, and Hirshfeld surface analysis were conducted. Finally, with the help of the theoretical data obtained, the results in different solvent environments are interpreted and compared with each other.

## 1 Introduction

With ozone depletion, a global problem, more UV radiation is reaching the Earth’s surface. It is widely acknowledged that extended exposure to ultraviolet (UV) radiation coupled with inadequate protective behaviors against UV exposure contributes significantly to both acute and chronic dermatological conditions, including various forms of skin cancer. Such exposure can also lead to detrimental effects on the integrity of the skin’s surface.^1^ However, simple changes in behavior can protect people from the harmful effects of UV radiation. One of these is protection with sunscreen products. Sunscreens are thought to protect the skin from many harmful effects of UV light thanks to the UV filters in them. UV filters provide UV protection in a broad band and have almost zero absorption of visible radiation, but exhibit notable light absorption characteristics within the ultraviolet A (UVA) range of 320 to 400 nm and the ultraviolet B (UVB) range of 280 to 320 nm.^2^ There are two main categories of UV filters utilized in sunscreens and other protective formulations. The first category comprises inorganic UV filters, commonly termed physical UV filters. These filters function primarily by reflecting and scattering UV radiation away from the skin. The second category consists of organic UV filters, also known as chemical UV filters, which operate by absorbing ultraviolet light.^3^ Chemical UV filters are organic substances and act by absorbing high-energy UV rays, i.e., a chemical UV filter effectively converts the high-energy UV photon into low-energy vibrations or heat by absorbing a photon and then rapidly releasing it through nonradiative pathways. The solar electromagnetic spectrum can be categorized into three groups, which include harmful ultraviolet (UV) radiation: UVA (320–400 nm), UVB (280–320 nm), and UVC (190–280 nm).^1−3^ Exposure of the skin to UVA and UVB radiation can result in various forms of damage, including erythema (sunburn), photoaging, pigmentation disorders, cutaneous malignancies, and an increased risk of both eye and skin cancers.^4,5^ Although UVC is highly detrimental, it is entirely absorbed by the Earth’s atmosphere. To mitigate the adverse effects of these harmful radiations, one of the primary constituents in sunscreens is organic UV filters, which are designed to absorb such radiation.^6^ Chemical and organic filters possess distinctive aromatic structures that can either be singular or consist of multiple interconnected units. These structures are frequently characterized by conjugated systems that include double carbon–carbon bonds and carbonyl functional groups, which contribute to their chemical stability and light-absorbing capabilities. One notable feature of these compounds is their high molar absorptivity in the ultraviolet (UV) region, making them particularly effective for sunlight protection. These filters are primarily categorized into two groups based on the wavelength of UV radiation they absorb: UVA absorbers, which target longer wavelengths (320–400 nm), and UVB absorbers, which are effective against shorter wavelengths (280–320 nm). Among the most prevalent UVB protectors are PABA (para-aminobenzoic acid) derivatives, cinnamates, salicylates, and camphor derivatives, each exhibiting specific absorption characteristics that help mitigate the harmful effects of UVB radiation on the skin. In contrast, effective UVA protectors include benzophenones, anthranilates, and dibenzoylmethanes, which provide a critical defense against the deeper penetration of UVA rays that can lead to long-term skin damage and aging. Organic filters are deliberately designed and engineered to absorb ultraviolet radiation efficiently, exhibiting varying degrees of effectiveness against UVA, UVB, or both types of radiation. During the course of our research, we primarily concentrated on the most widely used organic filters, recognized for their superior ability to absorb harmful UV rays. Our aim was to ensure comprehensive protection against sun exposure, exploring the molecular mechanisms behind their protective properties and their implications for skincare formulations. Through this investigation, we seek to contribute to the development of safer and more effective sun protection products.^1−3,7^ On the other hand, the importance of computer systems in the world of science and our daily lives is increasing day by day, and computational chemistry is constantly expanding as computer technology develops. Computational chemistry represents a specialized domain within the field of chemistry that utilizes computer simulations to address complex chemical issues. By integration of theoretical chemistry methodologies into sophisticated computer programs, this discipline enables the calculation of the structures, interactions, and properties of molecules. There are various methods in computational chemistry, including ab initio, quasiexperimental, and density functional theory. In recent years, DFT, one of these methods, has gained popularity due to its reliable results and is a widely used method in calculating the electron structure of atoms, molecules, and solids.^8,9^ It is not possible to determine many properties of molecules at the same time with a single experiment, but it can be determined in a short time with calculations that can be done with Gaussian, Quanta, Castep, Gamess, NWchem, Mopac, Orca, Quantum Espresso, and many similar software.^10^ With these calculations, the costly, months-long, and harmful process that creates unwanted byproducts is replaced by cheap, short, and harmless processes. The reliability of the data obtained by these theoretical methods is quite high.^11^ For this reason, they are widely used for calculations of molecules. Organic filters, although they cause different dermatological side effects, are better accepted by people than physical filters and attract more interest. However, the photodegradation of these organic filters is a major problem.^12^ This means that organic filters fail to provide adequate UV protection, with a potential imbalance in their UV absorption ability with prolonged sun exposure, resulting in an increase in skin-damaging reactive oxygen species (ROS).^13^ Various scientific formulation approaches such as different solvent/solvent systems,^14,15^ antioxidant agents,^13^ pH,^16^ different product combinations,^6,17,18^ and selective use of weak chromophores^6^ are used to ensure their photostabilization.

Research on the selection of solvents for sunscreen formulations remains surprisingly scarce in the existing literature. Additionally, the methods employed to assess their interactions with ultraviolet (UV) radiation, their UV-filtering capabilities, and their antioxidant properties often require extensive time and financial resources, as highlighted in various studies.^13,19,20^ The effectiveness of sunscreens is closely linked to the choice of solvents in which the active ingredients are dissolved, making solvent selection a critical aspect of formulation development. This study seeks to delve into the ultraviolet (UV) absorption spectra of sunscreen chemicals designed to block both UVA and UVB radiation. We chose these organic filters because the organic filters used in the study were designed to absorb UVA, UVB, or both.^15^ It specifically investigates the behaviors of these chemicals when dispersed in three distinct solvents selected for their varying polarities. These particular solvents were chosen based on their practical relevance in cosmetic formulations, as they can significantly affect the stability, absorption capabilities, and overall effectiveness of sunscreen products. By analyzing how these solvents influence the UV absorption characteristics of the sunscreen compounds, the research aims to provide valuable insights into optimizing sunscreen formulations, ultimately enhancing their protective efficacy against the harmful effects of UV radiation.^15,21^ To address the gaps in the current understanding of these interactions, the study employs DFT calculations, which will help in the theoretical exploration of these complex behaviors. Furthermore, the research aspires to answer fundamental scientific questions regarding the performance of organic UV filters in various solvent environments, paving the way for future experimental and theoretical inquiries related to other organic filters in sunscreen formulations. Moreover, the impaired function of retinoic acid-related orphan receptor γt (RORγ(t)), which is responsible for regulating the immune system, can cause inflammatory and autoimmune diseases and skin cancer. The activation of RORγ(t) in the skin, resulting from exposure to environmental chemicals, may contribute to the development of inflammatory skin disorders. UV filters, which are frequently utilized as additives in cosmetic and personal care products, have been extensively investigated for their endocrine-disrupting properties. Not much is known about the potential immune-impairing effects of UV-filtering chemicals, and RORγ activation caused by xenobiotics may impair immune responses.^22^ Therefore, this study evaluated whether the molecules studied could interact with RORγ(t) by using molecular docking. Moreover, the characteristics of organic filters’ ADME properties were investigated with the help of the SwissADME web server. Moreover, the approach of encapsulating UV filters in innovative hybrid materials proposed by Fantini et al.^23^ significantly reduces the need for stabilizers in formulations. It results in more effective sun protection products and safer, more environmentally friendly formulations. It will minimize negative impacts on health and the environment while advancing the development of effective and sustainable materials.^23,24^ Therefore, through this research, we aim to contribute to the development of environmentally sustainable materials. Consequently, this work serves as a fundamental step toward achieving our next goal.

## 2 Computational Details

The theoretical calculations of organic filters utilized in this study will be conducted employing density functional theory (DFT) with the assistance of the Gaussian 09W^25^ and GaussView 0.5^26^ software packages. The molecular structures analyzed will be evaluated in the gas, aqueous, *n*-hexane, and ethanol phases utilizing the 6-31G(d,p) basis set in conjunction with the B3LYP hybrid functional, which integrates Becke’s three-parameter hybrid functional (B3)^27^ and the Lee–Yang–Parr (LYP) functional.^28^ Following the geometry optimizations, to understand the effect of solvents on the chemical parameters and reactivity of organic filters and their electronic properties, oscillator strengths, absorption wavelengths, HOMO–LUMO orbital energies, and energy gaps in different phases (gas, water, ethanol, *n*-hexane) will be determined by TD-DFT/CPCM using the methods and basis sets described previously.^29^ NBO and NLMO analyses were conducted by using the same method. The electron localization function (ELF), noncovalent interactions (NCI), reduced density gradient (RDG) scatterplots, atoms in molecules (AIM), and Fukui analysis were conducted using the Multiwfn software^30^ and Visual Molecular Dynamics (VMD) software.^31^ The ADME features were evaluated utilizing the SwissADME web server.^32^ Molecular docking analysis was conducted using Auto Dock 4^33^ and Discovery Studio 2021 visualizer.^34^ The crystallographic structures of oxybenzone and avobenzone were obtained from the Cambridge Crystallographic Data Centre (CCDC) under reference numbers 1177255 and 1990014, respectively.^35^ The crystal packing diagrams were generated using free Mercury 4.0 software.^36^ Hirshfeld surface analysis for the organic UV-filters was conducted using CrystalExplorer 17.5.^37^

## 3 Results and Discussion

### 3.1 Optimized Molecular Structure

**Figure 1:**
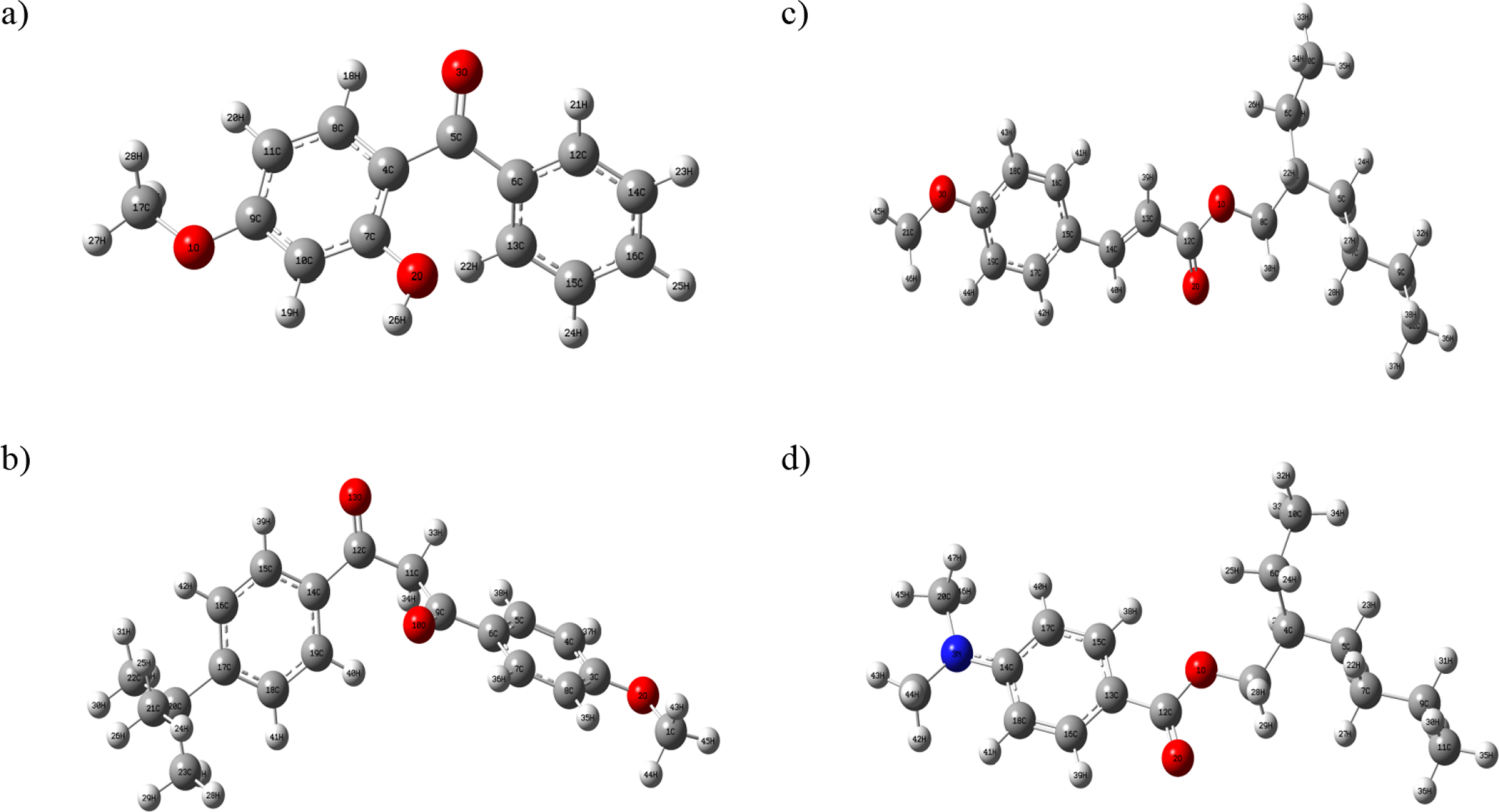
Optimized molecular structure of oxybenzone (a), avobenzone (b), octinoxate (c), and padimate O (d).

In order to perform the calculations specified in this study, it is essential to identify the most stable structures of the organic filters, including oxybenzone, avobenzone, octinoxate, and padimate O. This involves determining their optimized molecular structures, which will be computed in the gas phase using the 6-31G(d,p) basis set in conjunction with the B3LYP hybrid functional. The optimized molecular structures of the calculated organic filters are given in Figure 1, respectively.

### 3.2 Frontier Molecular Orbital (FMO) Analysis

**Figure 2:**
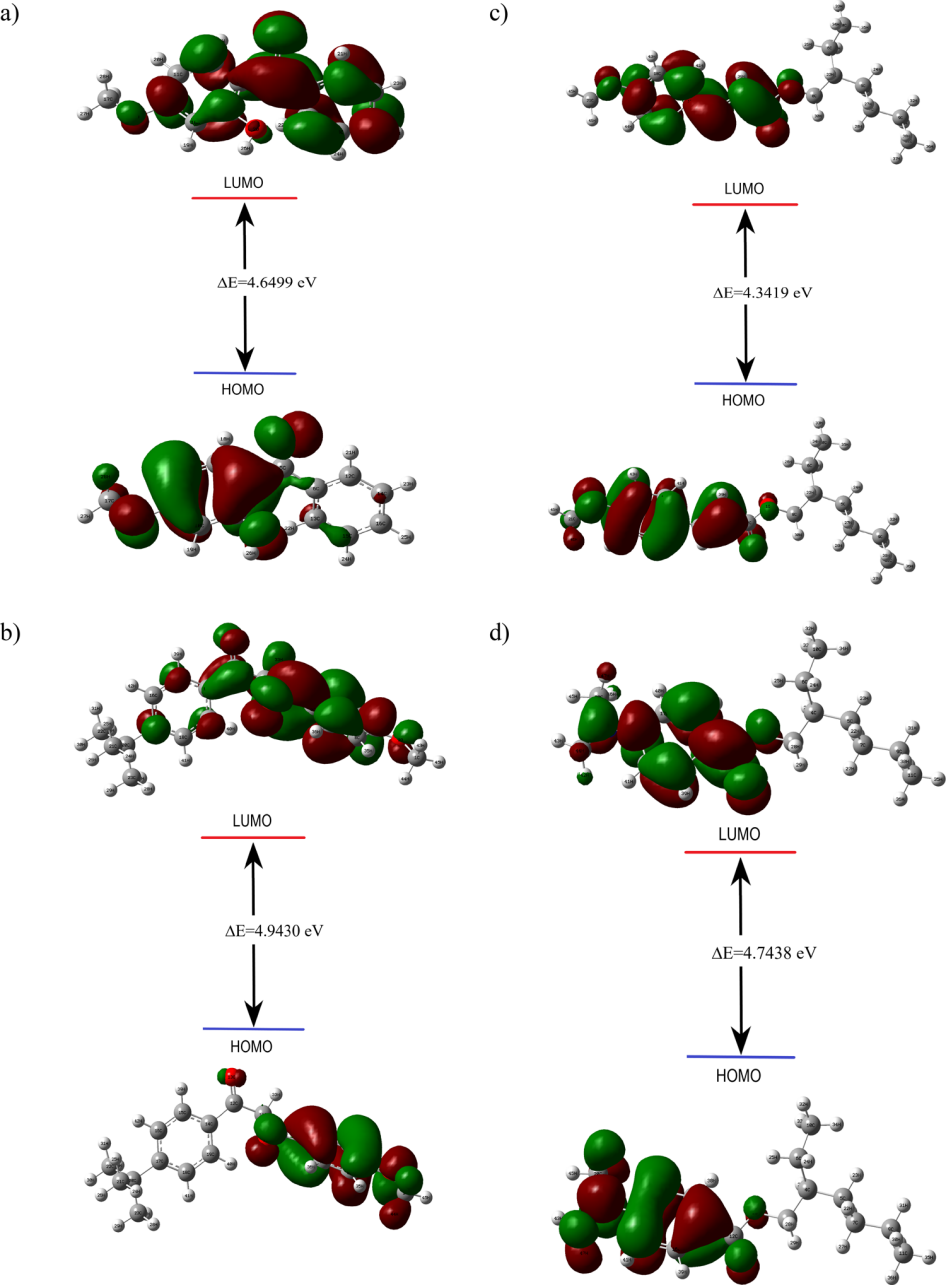
FMO diagram of oxybenzone (a), avobenzone (b), octinoxate (c), and padimate O (d).

The lowest unoccupied molecular orbital (LUMO) and the highest occupied molecular orbital (HOMO), collectively termed the frontier molecular orbitals (FMOs), play a crucial role in determining various molecular properties. These properties include, but are not limited to, chemical reactions, molecular stability, chemical reactivity, and optical and electrical characteristics.^38,39^ HOMO and LUMO analyses also give us information about the charge transfer within the molecule. Figure 2 depicts HOMO–LUMO orbitals for organic UV filters such as oxybenzone, avobenzone, octinoxate, and padimate O, respectively. With the help of LUMO and HOMO energies, electronegativity (χ), nucleophilic index (*N*), ionization potential (IP), chemical hardness (η), optical softness (σ_o_), chemical potential (μ), electron affinity (EA), chemical softness (ς), global electrophilicity index (ω), and maximum charge transfer index (Δ*N*_max_) are formulated as follows:^40−43^
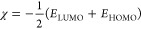
1
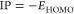
2
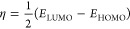
3
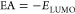
4
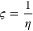
5
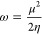
6
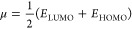
7
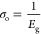
8
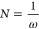
9
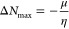
10

The chemical reactivity of a molecule can be determined by looking at the energy band gap between the LUMO and the HOMO orbitals. The smaller this energy, the lower the kinetic stability, the more polarizable, and the higher the chemical reactivity of that molecule.^38,39^ Chemical hardness and softness are qualitative indicators of how much a molecule’s electron cloud is perturbed in an electric field,^44^ and the energy band gap gives us information about these indicators. If the energy band gap (*E*_LUMO_ – *E*_HOMO_) is small, the molecule is chemically soft, and if it is large, it is chemically hard. Moreover, the low value of the chemical hardness indicates the strength of the electron-donating group in the molecule.^44^ The nonchemical meaning of hardness is resistance to deformation or change, while softness is the opposite.^45^

Chemical potential refers to the transfer of charge from a high chemical potential to a low chemical potential system. It depicts the tendency of an electron to escape and is known as chemical hardness versus behavior.^46,47^ To understand the strong and weak electrophilic properties of molecules, we need to look at their global electrophilicity. It is an indicator of the decrease in the total energy of a molecule due to electron motion between acceptor and donor groups in a molecule. According to a study by Domingo et al. in 2008:^48^ ω < 0.8 eV, weakly electrophilic; 0.8 < ω < 1.5 eV, moderately electrophilic; ω > 1.5 eV, highly electrophilic. The maximum charge transfer index indicates the maximum accepted charge (electrophilia) from the environment.^49^ Some chemical parameters of organic filters, such as frontier orbital analysis, chemical hardness, global electrophilicity, electronegativity, ionization energy, electron affinity, chemical potential, chemical softness, and maximum charge transfer index, were calculated theoretically and given in Table 1. Sahoo et al. reported that the energy gap of avobenzone in ethanol (DFT/IEFPCM method) is approximately 4.70 eV, which is in close agreement with our calculated value of 4.73 eV.^50^ Cooper et al. reported that the energy gap of oxybenzone is 3.92 eV and 316 nm, which is in close agreement with our calculated value of 4.01 eV and 309.04 nm (in hexane).^51^ When we compare the energy gap values of organic UV filters in the gas phase, it can be said that the octinoxate molecule has higher chemical reactivity than other molecules with an energy of 4.34 eV; i.e., more charge transfer occurs in the octinoxate molecule, while the avobenzone molecule has low chemical reactivity with an energy of 4.94 eV. The same result emerges when comparing the cases in other solvent media, i.e., the octinoxate is the most reactive molecule in all water, ethanol, and *n*-hexane media as well. According to Domingo et al.’s scale, we can say that in the gas phase, the octinoxate molecule has a more electrophilic character than other organic UV filters due to its higher electrophilic index of 3.05 eV, but in water and ethanol environments, avobenzone is more strongly electrophilic.

**Table 1:** Some Chemical Parameters of Organic Filters (a: Oxybenzone, b: Avobenzone, c: Octinoxate, and d: padimate O) Computed Using the TD-DFT Method, Both in Gas and Liquid Phases^a^

	gas				water				ethanol				hexane			
parameter (eV)	a	b	c	d	a	b	c	d	a	b	c	d	a	b	c	d
*E*_HOMO_	–6.0311	–6.3492	–5.8126	–5.3212	–6.1715	–6.3707	–5.9261	–5.4064	–6.1669	–6.3702	–5.9226	–5.4039	–6.0932	–6.3590	–5.8622	–5.3601
*E*_LUMO_	–1.3813	–1.4063	–1.4708	–0.5774	–1.6925	–1.6455	–1.6789	–0.8713	–1.6817	–1.6346	–1.6716	–0.8607	–1.5100	–1.4841	–1.5565	–0.6980
Δ*E*	4.6499	4.9430	4.3419	4.7438	4.4790	4.7253	4.2472	4.5351	4.4853	4.7356	4.2510	4.5432	4.5832	4.8749	4.3057	4.6621
IP	6.0311	6.3492	5.8126	5.3212	6.1715	6.3707	5.9261	5.4064	6.1669	6.3702	5.9226	5.4039	6.0932	6.3590	5.8622	5.3601
EA	1.3813	1.4063	1.4708	0.5774	1.6925	1.6455	1.6789	0.8713	1.6817	1.6346	1.6716	0.8607	1.5100	1.4841	1.5565	0.6980
χ	3.7062	3.8778	3.6417	2.9493	3.9320	4.0081	3.8025	3.1388	3.9243	4.0024	3.7971	3.1323	3.8016	3.9216	3.7093	3.0290
μ	–3.7062	–3.8778	–3.6417	–2.9493	–3.9320	–4.0081	–3.8025	–3.1388	–3.9243	–4.0024	–3.7971	–3.1323	–3.8016	–3.9216	–3.7093	–3.0290
η	2.3249	2.4715	2.1709	2.3719	2.2395	2.3626	2.1236	2.2675	2.2426	2.3678	2.1255	2.2716	2.2916	2.4375	2.1528	2.3311
ζ	0.4301	0.4046	0.4606	0.4216	0.4465	0.4233	0.4709	0.4410	0.4459	0.4223	0.4705	0.4402	0.4364	0.4103	0.4645	0.4290
ω	2.9540	3.0421	3.0545	1.8337	3.4519	3.3998	3.4044	2.1725	3.4335	3.3827	3.3917	2.1596	3.1532	3.1547	3.1956	1.9680
Δ*N*_max_	1.5941	1.5690	1.6775	1.2434	1.7558	1.6965	1.7906	1.3843	1.7499	1.6903	1.7865	1.3789	1.6589	1.6089	1.7230	1.2994
σ_o_	0.2151	0.2023	0.2303	0.2108	0.2233	0.2116	0.2355	0.2205	0.2230	0.2112	0.2352	0.2201	0.2182	0.2051	0.2323	0.2145
*N*	0.3385	0.3287	0.3274	0.5454	0.2897	0.2941	0.2937	0.4603	0.2912	0.2956	0.2948	0.4631	0.3171	0.3170	0.3129	0.5081

a Δ*E*: Energy gap; *E*_LUMO_: Energy of LUMO orbital; *E*_HOMO_: Energy of HOMO orbital; *N*: Nucleophilic index; χ: Electronegativity; ω: Global electrophilicity index; IP: Ionization potential; η: Chemical hardness; μ: Chemical potential; EA: Electron affinity; Δ*N*_max_: Maximum charge transfer index; σ_o_: Optical softness; ζ: Chemical softness.

### 3.3 UV–Vis Analysis

**Figure 3:**
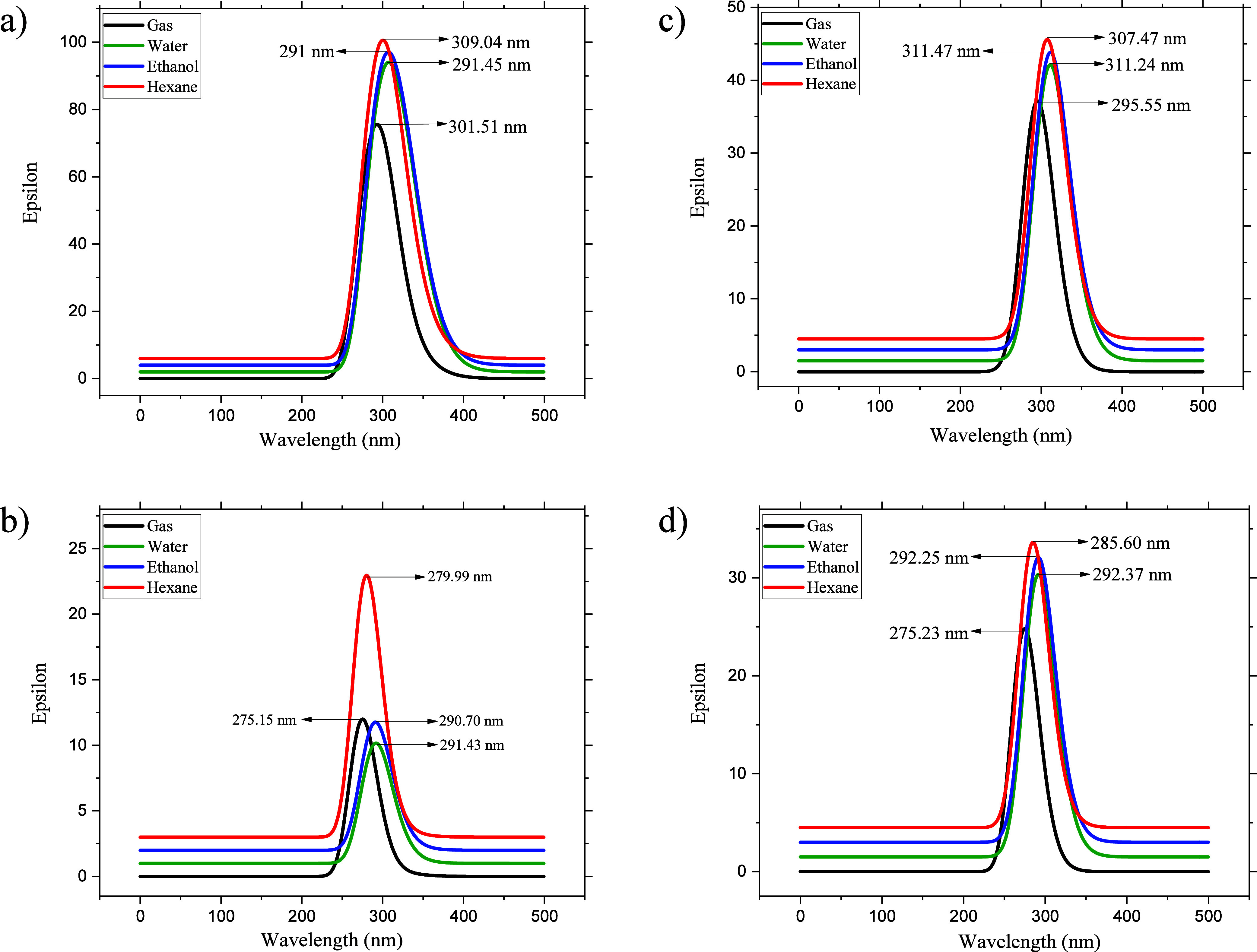
Theoretical UV–vis spectra of oxybenzone (a), avobenzone (b), octinoxate (c), and padimate O (d) in gas and water, ethanol, and *n*-hexane as solvents.

TD-DFT is a widely used method to find out the UV–vis spectra of molecules.^52,53^ For molecules, UV–vis spectra and some absorption parameters such as absorption wavelengths, excitation energies, oscillator strengths, and transition probabilities to HOMO–LUMO orbitals were analyzed by the TD-DFT approach using gas, water, *n*-hexane, and ethanol solvents by choosing the CPCM model, as shown in Table 2. Figure 3 depicts UV–vis spectra for organic UV filters such as oxybenzone, avobenzone, octinoxate, and padimate O in gas, water, ethanol, and *n*-hexane mediums, respectively. The theoretical UV spectra plotted were compared, and different absorption peaks were observed in different solvents. According to the results, the molecule with the maximum excitation energy (4.5061 eV) and lowest absorbance (275.15 nm) in the gas phase is avobenzone. Similarly, the molecule with the maximum excitation and lowest wavelength in solvent environments such as water, ethanol, and *n*-hexane is avobenzone. The excitation energy of avobenzone increases in the order water < ethanol < *n*-hexane < gas. This is similar to the other two molecules except for oxybenzone. The photostability of organic UV filters is important because they can interact with solvents and formulation components. It must therefore be effective and stable. For example, avobenzone is a chemical compound that exists in two distinct forms: the unstable keto form and the more stable enol form. The instability inherent to the keto form contributes to its susceptibility to photodegradation. However, it is noteworthy that this compound can readily undergo conversion to the enol form when in solution. The photodegradation kinetics of these filters in different solvents are also different. In dilute solution, the dominant photochemical reaction in an organic solvent is photoisomerization, where a photostationary state is observed.^14,21,54,55^ Investigations have also examined the photostability of the studied organic filters in solvent media and have shown that the maximum absorption wavelength (λ_max_) is affected by the polarity of the solvent used.^14,21,54^ For example, in polar solvents such as dimethyl sulfoxide and methanol, avobenzone exhibits a bathochromic shift, indicating an absorption at longer wavelengths. Conversely, in nonpolar solvents like ethyl acetate and cyclohexane, avobenzone experiences a hypochromic shift, resulting in absorption at shorter wavelengths.^14,54^ This result is consistent with our results calculated by the DFT method. Avobenzone shows high wavelength absorption in polar solvents like water and ethanol, while in apolar solvents like hexane it results in absorption at shorter wavelengths.^14,54^ This trend is similar for organic filters other than oxybenzone.^54^ This behavior of avobenzone is consistent with the properties observed in most sunscreen formulations.

**Table 2:** Absorption Data of Organic Filters (a: Oxybenzone, b: Avobenzone, c: Octinoxate, and d: Padimate O) Computed Using the TD-DFT Method, Both in Gas and Liquid Phases^a^

	a					b					c					d				
medium	state trans.	*E*_ex_ (eV)	λ (nm)	*f* (eV)	*P*_trans_ (%)	state trans.	*E*_ex_ (eV)	λ (nm)	*f* (eV)	*P*_trans_ (%)	state trans.	*E*_ex_ (eV)	λ (nm)	*f* (eV)	*P*_trans_ (%)	state trans.	*E*_ex_ (eV)	λ (nm)	*f* (eV)	*P*_trans_ (%)
gas	S_0_ → S_2_	4.1122	301.51	0.1346	40 (H-1 → L)	S_0_ → S_3_	4.5061	275.15	0.0296	30 (H-4 → L)	S_0_ → S_1_	4.1950	295.55	0.9118	98 (H → L)	S_0_ → S_1_	4.5048	275.23	0.5973	2 (H-1 → L+1)
					57 (H → L)					3 (H-4 → L+1)										97 (H → L)
										23 (H-2 → L)										
										7 (H-2 → L+1)										
										23 (H-1 → L)										
										6 (H-1 → L+1)										
										6 (H → L)										
water	S_0_ → S_3_	4.2541	291.45	0.1303	5 (H-4 → L)	S_0_ → S_3_	4.2544	291.43	0.0223	94 (H → L)	S_0_ → S_1_	3.9836	311.24	1.0022	99 (H → L)	S_0_ → S_1_	4.2407	292.37	0.6979	98 (H → L)
					24 (H-2 → L)					5 (H → L+1)										
					60 (H-1 → L)															
					8 (H → L)															
					2 (H → L+1)															
ethanol	S_0_ → S_3_	4.2606	291.00	0.1305	5 (H-4 → L)	S_0_ → S_3_	4.2651	290.70	0.0238	94 (H → L)	S_0_ → S_1_	3.9807	311.47	1.0075	99 (H → L)	S_0_ → S_1_	4.2424	292.25	0.7030	98 (H → L)
					24 (H-2 → L)					5 (H → L+1)										
					59 (H-1 → L)															
					8 (H → L)															
					2 (H → L+1)															
hexane	S_0_ → S_2_	4.0119	309.04	0.1607	7 (H-2 → L)	S_0_ → S_3_	4.4281	279.99	0.0492	69 (H → L)	S_0_ → S_1_	4.0324	307.47	1.0135	99 (H → L)	S_0_ → S_1_	4.3412	285.60	0.7019	99 (H → L)
					32 (H-1 → L)					29 (H → L+1)										
					57 (H → L)															

a trans., transition; *P*_trans_, probability of transition; *E*_ex_, excitation energy; *f*, oscillator strength; λ, wavelength.

Furthermore, the λ_max_ values obtained from these investigations confirm that these organic filters serve as effective UVA and UVB absorbers and play a crucial role in safeguarding the skin from the detrimental effects of ultraviolet radiation.^54^ In literature studies, the effect of solvents on the wavelength can be given as follows. The maximum wavelengths of avobenzone were found to be 363 nm (DMSO), 360 and 358 nm (methanol), 356 nm (ethyl acetate), 351 nm (cyclohexane), and 350 nm (ethanol).^14,54,55^ The maximum wavelengths of oxybenzone were found to be 328 nm (hexane), 325 nm (ethanol), 324 nm (ethanol-90%-water-10%), 321 nm (ethanol-70%-water-30%), and 286 nm (methanol-85%-water-15%).^21^ The maximum wavelengths of octinoxate were found to be 308 nm (methanol)^56^ and 312 nm (ethanol).^57^ The maximum wavelengths of Padimate O were found to be 272 nm (ethanol), 271 nm (ethanol-90%-water-10%), and 266 nm (ethanol-70%-water-30%).^21^ Additionally, the spectral range is reported to be from 300 nm in nonpolar solvents to 316 nm in polar solvents.^58^

### 3.4 NBO/NLMO/AIM Analysis

NBO analysis represents an effective method to study attractive properties of a molecular system. It offers a powerful perspective on intra- and intermolecular bonding and the interaction between bonds and it can also facilitate the understanding and exploration of charge transfer or conjugative interactions within the molecular system.^59,60^ Another useful aspect of the NBO method is that it provides information about interactions in both occupied and virtual orbital spaces, which can aid in a detailed analysis of intramolecular and intermolecular interactions. A second-order Fock matrix was used to evaluate donor–acceptor interactions in NBO analysis.^59,60^ For each donor NBO (*i*) and recipient NBO (*j*), the stabilization energy associated with *i* → *j* delocalization can be estimated as
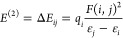
11

NBO analysis of the organic UV filters was carried out at the B3LYP/6-31G(d,p) level of theory to understand the second-order interactions between donor and acceptor orbitals, and the interactions are presented in Tables 3–6. The detailed interactions are given in Tables S1–S4. In the NBO analysis, larger *E*^(2)^ values indicate that the interaction between electron acceptors and electron donors is more intense. According to Table 3, the strong intramolecular conjugative interaction between LP(2)O1 and the antibond of C9–C11 (*n*–π*) of the oxybenzone has the maximum *E*^(2)^ value around 32.28 kcal/mol. According to Table 3, the stabilization energy values of the donor and acceptor interactions, the most significant transitions in the oxybenzone are LP(2) O2 to C7–C10 (*n*–π*) with 30.68 kcal/mol, C9–C11 to C4–C8 (π–π*) with 26.48 kcal/mol, C4–C8 to C7–C10 (π–π*) with 26.3 kcal/mol, and C7–C10 to C9–C11 (π–π*) with 24.36 kcal/mol. In the avobenzone, the strong intramolecular conjugative interaction between LP(2) O2 and the antibond of C3–C8 (*n*–π*) has the maximum *E*^(2)^ value around 32.78 kcal/mol. According to Table 4, the stabilization energy values of the donor and acceptor interactions, the most significant transitions in the avobenzone, C3–C8 to C6–C7 (σ–σ*) with 25.44 kcal/mol, C17–C18 to C14–C19 (π–π*) with 23.12 kcal/mol, C6–C7 to C4–C5 (π–π*) with 22.99 kcal/mol, C4–C5 to C3–C8 (π–π*) with 22.3 kcal/mol, LP(2)O10 to C9–C11 (*n*–σ*) with 22.21 kcal/mol, and LP(2)O13 to C11–C12 (*n*–σ*) with 20.62 kcal/mol. In the octinoxate and padimate O is noted that the strong intramolecular conjugative interactions between LP(2) O1 and the antibonding of O2–C12 (*n*–π*), which have the maximum *E*^(2)^ value around 46.84 and 46.13 kcal/mol, respectively. According to Table 5, the stabilization energy values of the donor and acceptor interactions, the most significant transitions in the octinoxate, LP(2)O2 to O1–C12 (*n*–σ*) with 34 kcal/mol, LP(2)O3 to C19–C20 (*n*–σ*) with 32.12 kcal/mol, C19–C20 to C15–C17 (π–π*) with 22.83 kcal/mol, C13–C14 to O2–C12 (π–π*) with 22 kcal/mol, and LP(2)O2 to C12–C13 (*n*–σ*) with 17.69 kcal/mol. According to Table 6, the stabilization energy values of the donor and acceptor interactions, the most significant transitions in the padimate O, LP(1)N3 to C14–C17 (*n*–π*) with 45.83 kcal/mol, LP(2)O2 to O1–C12 (*n*–σ*) with 33.93 kcal/mol, C14–C17 to C13–C15 (π–π*) with 26.94 kcal/mol, C13–C15 to O2–C12 (π–π*) with 25.43 kcal/mol, C13–C15 to C16–C18 (π–π*) with 23.96 kcal/mol, and LP(2)O2 to C12–C13 (*n*–σ*) with 17.55 kcal/mol. The other important interactions for organic UV filters are listed in Tables 3–6.

**Table 3:** Fock Matrix of Oxybenzone Analyzed through Second Order Perturbation Theory Using the NBO Method

no.	donor (*i*)	type	acceptor (*j*)	type	*E*^(2)^ (kJ mol^–1^)	*E*(*j*) – *E*(*i*) (au)	*F*(*i*, *j*) (au)
1	C4–C8	π	O3–C5	π*	13.97	0.27	0.057
2	C4–C8	π	C7–C10	π*	26.3	0.27	0.076
3	C4–C8	π	C9–C11	π*	14.84	0.27	0.057
4	C6–C13	π	O3–C5	π*	16.8	0.26	0.063
5	C6–C13	π	C12–C14	π*	20.31	0.28	0.069
6	C6–C13	π	C15–C16	π*	19.3	0.28	0.066
7	C7–C10	π	C4–C8	π*	13.12	0.3	0.057
8	C7–C10	π	C9–C11	π*	24.36	0.29	0.077
9	C9–C11	π	C4–C8	π*	26.48	0.29	0.079
10	C9–C11	π	C7–C10	π*	14.55	0.28	0.057
11	C12–C14	π	C6–C13	π*	18.96	0.28	0.066
12	C12–C14	π	C15–C16	π*	21.85	0.28	0.070
13	C15–C16	π	C6–C13	π*	21.54	0.29	0.070
14	C15–C16	π	C12–C14	π*	17.97	0.29	0.065
15	LP(2)O1		C9–C11	π*	32.28	0.34	0.100
16	LP(2)O2		C7–C10	π*	30.68	0.35	0.098
17	LP(2)O3		C4–C5	σ*	19.35	0.69	0.104
18	LP(2)O3		C5–C6	σ*	18.64	0.69	0.103

**Table 4:** Fock Matrix of Avobenzone Analyzed through Second Order Perturbation Theory Using the NBO Method

no.	donor (*i*)	type	acceptor (*j*)	type	*E*^(2)^ (kJ mol^–1^)	*E*(*j*) – *E*(*i*) (au)	*F*(*i*, *j*) (au)
1	C3–C8	σ	C4–C5	σ*	15.28	0.29	0.061
2	C3–C8	σ	C6–C7	σ*	25.24	0.29	0.077
3	C4–C5	π	C3–C8	π*	22.3	0.28	0.072
4	C4–C5	π	C6–C7	π*	15.55	0.29	0.061
5	C6–C7	π	C3–C8	π*	16.97	0.26	0.060
6	C6–C7	π	C4–C5	π*	22.99	0.28	0.072
7	C6–C7	π	C9–O10	π*	21.06	0.27	0.071
8	C14–C19	π	C12–O13	π*	19.29	0.27	0.069
9	C14–C19	π	C15–C16	π*	19.89	0.29	0.069
10	C14–C19	π	C17–C18	π*	17.8	0.29	0.064
11	C15–C16	π	C14–C19	π*	18	0.28	0.064
12	C15–C16	π	C17–C18	π*	21.61	0.29	0.070
13	C17–C18	π	C14–C19	π*	23.12	0.28	0.072
14	C17–C18	π	C15–C16	π*	16.38	0.29	0.062
15	LP(2)O2		C3–C8	π*	32.78	0.34	0.099
16	LP(2)O10		C6–C9	σ*	18.98	0.71	0.105
17	LP(2)O10		C9–C11	σ*	22.21	0.63	0.107
18	LP(2)O13		C11–C12	σ*	20.62	0.64	0.104
19	LP(2)O13		C12–C14	σ*	19.16	0.71	0.105

**Table 5:** Fock Matrix of Octinoxate Analyzed through Second Order Perturbation Theory Using the NBO Method

no.	donor (*i*)	type	acceptor (*j*)	type	*E*^(2)^ (kJ mol^–1^)	*E*(*j*) – *E*(*i*) (au)	*F*(*i*, *j*) (au)
1	C13–C14	π	O2–C12	π*	22	0.29	0.074
2	C13–C14	π	C15–C17	π*	11.18	0.3	0.055
3	C15–C17	π	C13–C14	π*	17.9	0.29	0.069
4	C15–C17	π	C16–C18	π*	20.37	0.28	0.069
5	C15–C17	π	C19–C20	π*	17.77	0.27	0.062
6	C16–C18	π	C15–C17	π*	16.05	0.29	0.062
7	C16–C18	π	C19–C20	π*	21.33	0.28	0.071
8	C19–C20	π	C15–C17	π*	22.83	0.29	0.074
9	C19–C20	π	C16–C18	π*	15.3	0.3	0.061
10	LP(2)O1		O2–C12	π*	46.84	0.33	0.113
11	LP(2)O2		C12–C13	σ*	17.69	0.7	0.102
12	LP(2)O3		C19–C20	π*	32.12	0.34	0.099

**Table 6:** Fock Matrix of Padimate O Analyzed through Second Order Perturbation Theory Using the NBO Method

no.	donor (*i*)	type	acceptor (*j*)	type	*E*^(2)^ (kJ mol^–1^)	*E*(*j*) – *E*(*i*) (au)	*F*(*i*, *j*) (au)
1	C13–C15	π	O2–C12	π*	25.43	0.26	0.074
2	C13–C15	π	C14–C17	π*	16.31	0.26	0.060
3	C13–C15	π	C16–C18	π*	23.96	0.28	0.074
4	C14–C17	π	C13–C15	π*	26.94	0.28	0.079
5	C14–C17	π	C16–C18	π*	13.92	0.29	0.058
6	C16–C18	π	C13–C15	π*	14.36	0.29	0.059
7	C16–C18	π	C14–C17	π*	22.04	0.27	0.072
8	LP(2)O1		O2–C12	π*	46.13	0.33	0.113
9	LP(2)O2		O1–C12	σ*	33.96	0.62	0.132
10	LP(2)O2		C12–C13	σ*	17.55	0.7	0.102
11	LP(1)N3		C14–C17	π*	45.83	0.27	0.103

NLMO analysis helps to understand how binding in a molecule is formed from orbitals localized on different atoms. NLMOs are derived from NBOs and provide direct insight into the nature of the “delocalization tails” of the localized molecular orbital.^61,62^Table 7 shows the significant NLMO occupancy, the percentage of the parent NBO, and the atomic hybrid contributions of the organic UV filters calculated at the B3LYP level using the 6-31G(d,p) basis set. The detailed NLMO occupancies are given in Tables S5–S8. In the oxybenzone, BD (2) C12-C14 is the most delocalized NLMO, having around 81.1% of contribution from the parent NBO. The localization tail (∼18.9%) consists of hybrids of O3, C5, C6, C13, C15, and C16. In the avobenzone, BD (2) C6-C7 is the most delocalized NLMO, having around 80.20% of contribution from the parent NBO. The localization tail (∼19.8%) consists of hybrids of C3, C4, C5, C8, C9, and O10. In the octinoxate, BD (2) C19-C20 is the most delocalized NLMO, having around 80.82% of contribution from the parent NBO. The localization tail (∼19.18%) consists of hybrids of O1, O2, O3, C12, C13, C14, C15, C16, C17, C18, and C21.

**Table 7:** Organic Filter’s Significant NLMO Occupancy, Percentage Derived from Its Parent NBO, and Atomic Hybrid Contributions

				hybrid contributions	
no.	bond	occupancy	percentage from parent NBO (%)	atom	percentage (%)
Oxybenzone					
1	LP(2)O1	2.00000	91.2035	O1	91.204
2	LP(1)O2	2.00000	98.9155	O2	98.916
3	LP(2)O2	2.00000	93.2204	O2	93.220
4	LP(1)O3	2.00000	98.9268	O3	98.927
5	LP(2)O3	2.00000	94.3765	O3	94.378
Avobenzone					
1	LP(1)O2	2.00000	98.1849	O2	98.185
2	LP(2)O2	2.00000	91.2336	O2	91.234
3	LP(1)O10	2.00000	98.8544	O10	98.854
4	LP(2)O10	2.00000	94.2895	O10	94.290
5	LP(1)O13	2.00000	98.8758	O13	98.876
6	LP(2)O13	2.00000	94.4746	O13	94.475
Octinoxate					
1	LP(1)O1	2.00000	98.1597	O1	98.160
2	LP(2)O1	2.00000	89.1463	O1	89.146
3	LP(1)O2	2.00000	98.8205	O2	98.821
4	LP(2)O2	2.00000	92.1420	O2	92.142
5	LP(1)O3	2.00000	98.1803	O3	98.181
6	LP(2)O3	2.00000	91.4526	O3	91.453
Padimate O					
1	LP(1)O1	2.00000	98.0790	O1	98.080
2	LP(2)O1	2.00000	89.6562	O1	89.656
3	LP(1)O2	2.00000	98.8295	O2	98.830
4	LP(2)O2	2.00000	92.3337	O2	92.334
5	LP(1)N3	2.00000	84.5573	N3	84.559

In the padimate of O, BD (2) C14-C17 is the most delocalized NLMO, having around 78.91% of contribution from the parent NBO. The localization tail (∼21.09%) consists of hybrids of O1, O2, N3, C12, C13, C15, C16, C18, C19, C20, and C21. The significant localization tail of an NLMO indicates that they are not strongly localized in the vicinal regions.

**Figure 4:**
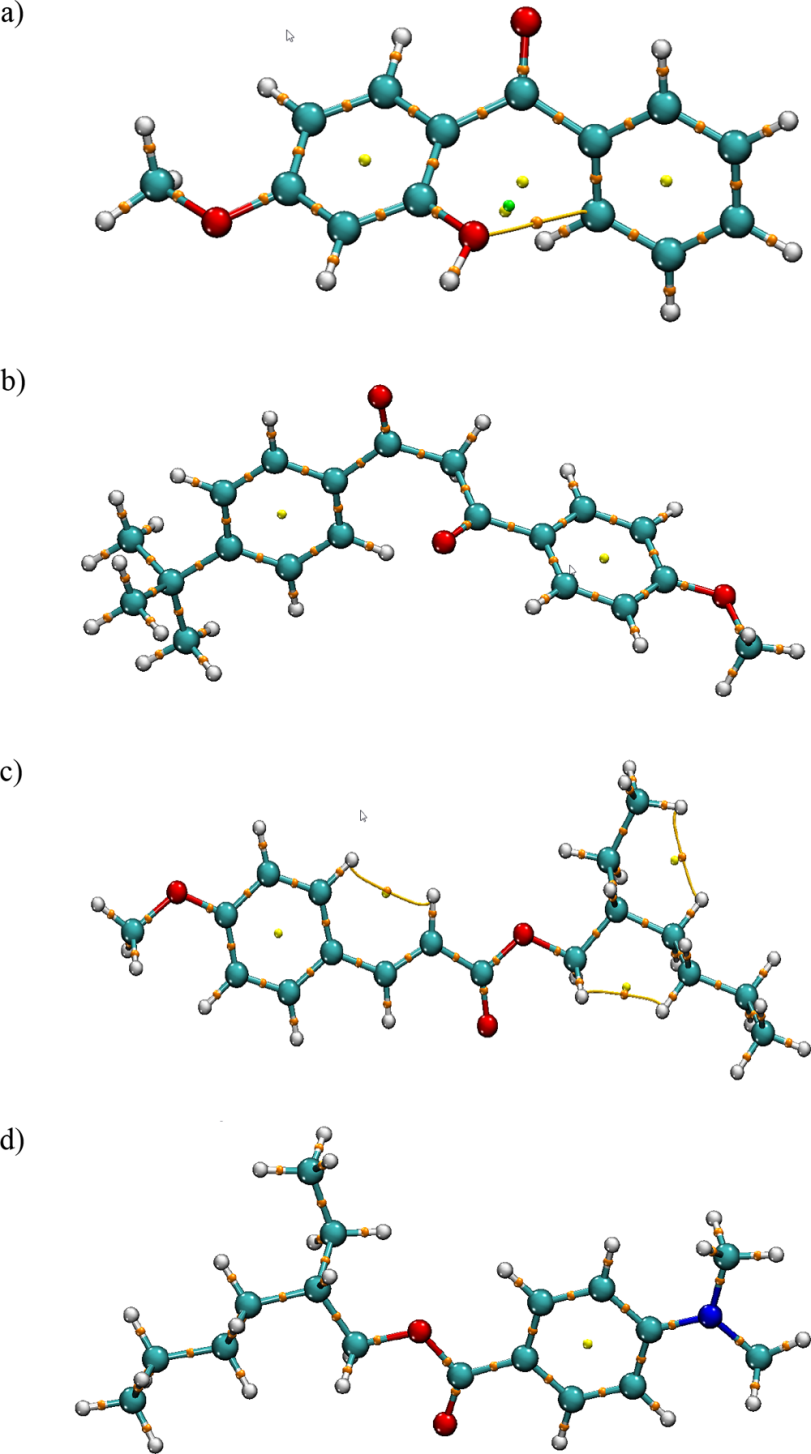
AIM analysis of oxybenzone (a), avobenzone (b), octinoxate (c), and padimate O (d).

The theory of atoms in molecules (AIM) was proposed by Bader widely used to determine the different types of interactions in various molecular systems.^63^ AIM analysis was used to obtain intramolecular BCPs (bond critical points) of the optimized organic filters, as shown in Figure 4.

According to the results obtained with the Multiwfn program, one BCP was found in oxybenzone, and three BCPs were found in octinoxate. In avobenzone and padimate O, no BCPs were found in the molecules. In oxybenzone, we observed an O2···C13 type of interaction, where the electron density value (ρ(*r*)) is 0.0099 au and the values of Laplacian of electron density (∇^2^ρ(*r*)) is 0.0361 au, potential energy density *V*(*r*) is −0.0066 au, and interaction energy (*E*_int_ = *V*(*r*)/2) is −2.07 kcal/mol, respectively. In octinoxate, we observed H28···H30, H39···H41 and H24···H35 type of interactions, where the electron density values (ρ(*r*)) are 0.0087, 0.0081, 0.0090 au and the values of Laplacian of electron density (∇^2^ρ(*r*)) are 0.0350, 0.0362, 0.0355 au. The potential energy density *V*(*r*) is −0.0046, −0.0042, and −0.0049 au, and the interaction energies^64^ (*E*_int_ = *V*(*r*)/2) are −1.44, −1.32, and −1.54 kcal/mol, respectively. We can say that these interactions in oxybenzone and octinoxate molecules contribute to the stability of the molecules.

### 3.5 Molecular Electrostatic Potential (MEP)

**Figure 5:**
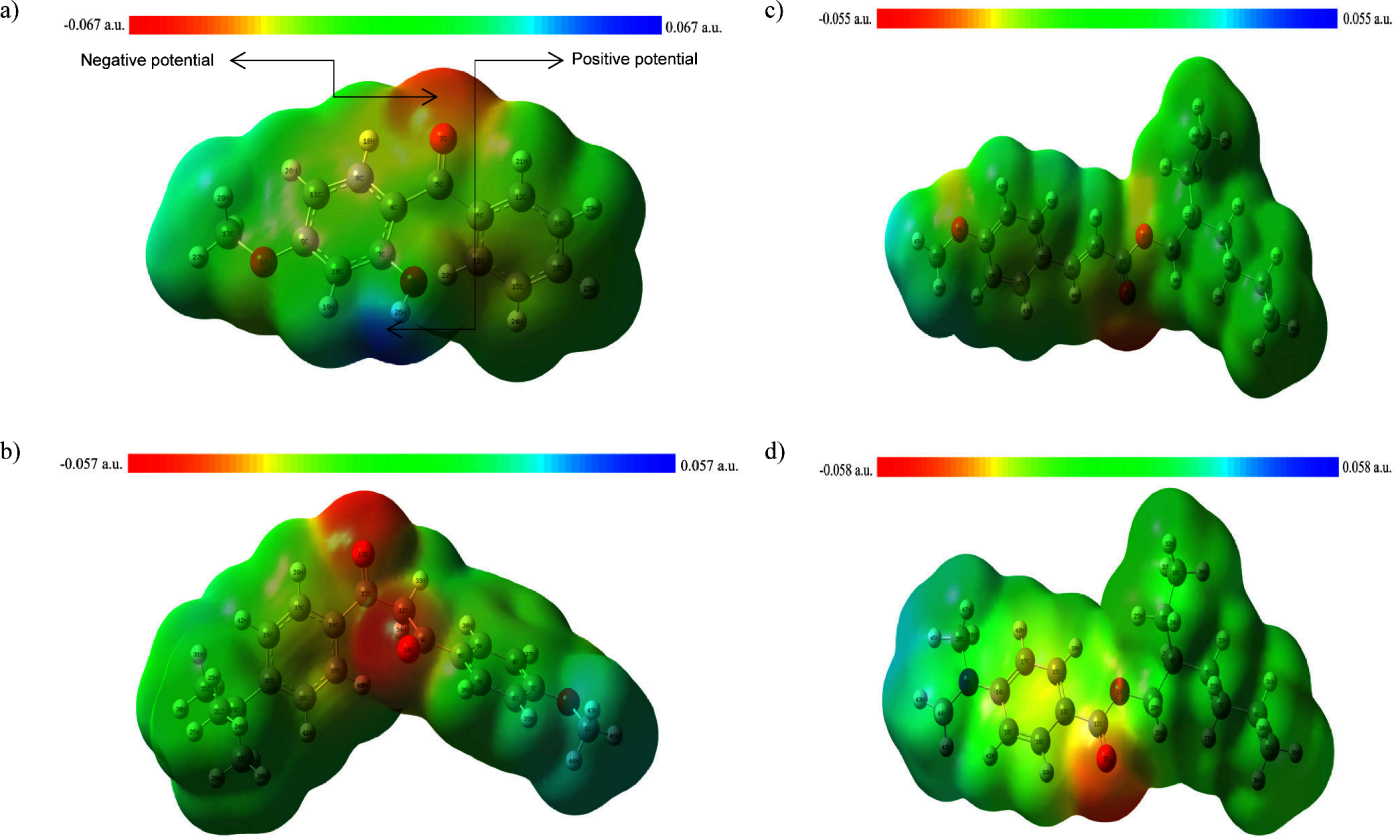
MEP surfaces of oxybenzone (a), avobenzone (b), octinoxate (c), and padimate O (d).

MEP surface analysis is an important method used to determine the electron-poor regions, i.e., nucleophilic attack zones, and electron-rich regions, i.e., electrophilic attack zones of molecules, where molecules are electrophilically and nucleophilically reactive, hydrogen bonding interactions, and electron-dense regions on the molecular surface.^60,65,66^ In addition, molecular shape and size indicate positive, negative, and neutral electrostatic potential regions.^60,65,66^ In short, MEP provides a visual method to understand the reactivity of a system and provides information based on color changes. Figure 5 depicts MEP surfaces for organic UV filters, such as oxybenzone, avobenzone, octinoxate, and padimate O, respectively. In the MEP surface map, red regions, i.e., regions with negative electrostatic potential, indicate the electron-dense region, while blue regions, i.e., regions with positive electrostatic potential, indicate electron-poor regions. The colors on the MEP map indicate different values of electrostatic potential. The potential increases from red to blue (red < orange < yellow < green < blue).^60,65,66^ Therefore, the MEP surface map is shown with different colors starting from the electron-rich region (red) toward the electron-poor region (blue). According to Figure 5a, the negative electrostatic potential zones related to electrophilic attack (in red) are mostly on the oxygen atom of the carbonyl group, while the positive electrostatic potential zones related to nucleophilic attack (in blue) are on the hydrogen of the hydroxyl group. In Figure 5b, the negative electrostatic potential zones are mostly on the oxygen atoms of carbonyl groups, while the positive electrostatic potential zones are on the methyl group linked to oxygen. In Figure 5c, the negative electrostatic potential zones are mostly on the oxygen atom of the carbonyl group, while the positive electrostatic potential zones are on the methyl group linked to oxygen. In Figure 5d, the negative electrostatic potential zones are mostly on the oxygen atom of the carbonyl group, while the positive electrostatic potential zones are on methyl groups linked to nitrogen.

### 3.6 NCI-RDG Analysis

The NCI method was used here to better understand and know the different types of intramolecular interactions of organic filters in different solvent media. The RDG approach is a dimensionless quantity obtained from the electron density and its first derivative. It was developed by Johnson et al. and can be described by the following formula.^67^
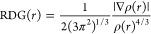
12

**Figure 6:**
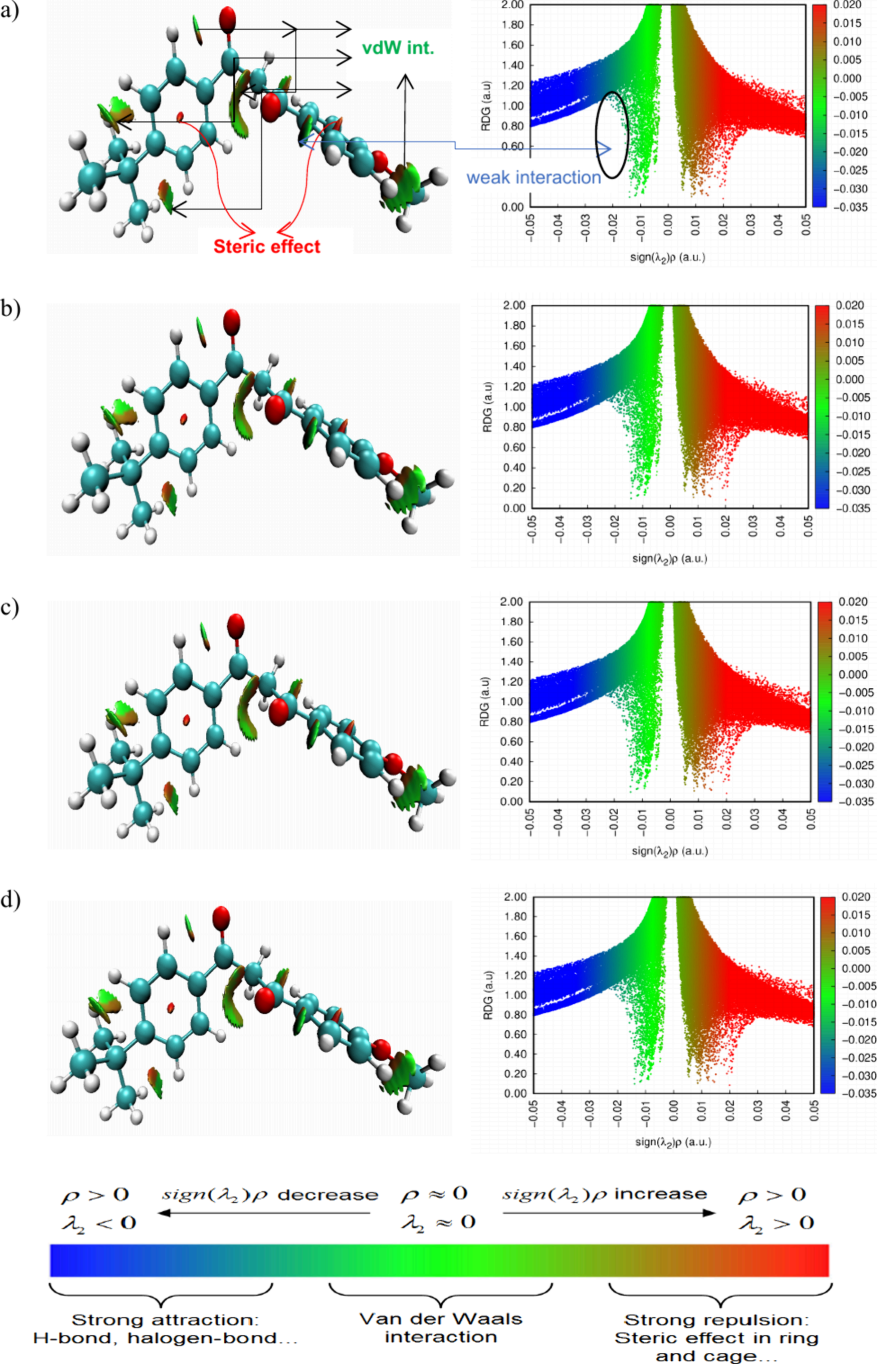
RDG (right)-NCI(left) analysis of avobenzone in various solvents: gas (a), water (b), ethanol (c), and *n*-hexane (d).

The NCI-RDG analyses were conducted by an isosurface value of 0.6, and are indicated, respectively, in gas and liquid phase. The 3D isosurface visualizer VMD software and Multiwfn software are used to produce the RDG scatter plots. The 3D isosurface and 2D RDG plots are indicated in Figure 6. According to Figure 6, the yellow, blue, and red colors in the RDG graph indicate van der Waals and hydrogen bond interactions, and steric effects, respectively. In studying the molecule (avobenzone), steric and van der Waals interactions were observed in all solvent environments, while hydrogen bonding interactions were not observed. In NCI analysis, intramolecular interactions are indicated by red, yellow, and blue discs. The red, blue, and green discs represent the evidence of significant steric repulsions or strong electrostatic interactions, strong attractive hydrogen bonds, and very weak interactions like H−π interactions and π–π dispersion interactions, respectively.^41,68^ Furthermore, the green regions, which are predominantly observed between hydrogen and electronegative atoms, indicate attractive forces that are critical for maintaining molecular stability.^68^ In the studied molecule, similar to the RDG analysis, the presence of steric effects and weak interactions can be mentioned, but not hydrogen bond interactions.^68^ That is, in all solvent environments, no significant changes were observed in the intramolecular interactions of avobenzone, and no hydrogen bond interactions were observed. Finally, avobenzone is a molecule notable for having the highest excitation energy and the shortest wavelength when evaluated in gas and various solvent environments, including water, ethanol, and *n*-hexane. To understand the role of noncovalent interactions in this behavior and maintaining molecular stability, we set out to analyze the intramolecular interactions of avobenzone across these different solvents. Our investigation revealed that there were no significant interactions present in any of the solvent environments studied. As a result of these findings, we concluded that further calculations of other molecules were unnecessary.

### 3.7 Analysis of Fukui Function

Fukui functions are employed to identify the most reactive regions for nucleophilic, electrophilic, and radical reactions within a molecule.^60,69^ In this context, the computation of Fukui functions was facilitated using Multiwfn software with the relevant expressions being as follows:
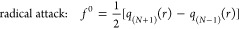
13
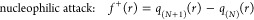
14
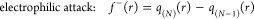
15
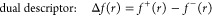
16

In the presented equations, *q*_(*N*+1)_(*r*), *q*_(*N*)_(*r*), and *q*_(*N*-1)_(*r*) represent the atomic charge at zone *r* within an anionic system, the atomic charge at zone *r* within a neutral system and the atomic charge at zone *r* within a cationic system, respectively.^60,69^ The dual descriptor is used to determine the molecule’s behavior of giving or accepting electrons during a reaction, i.e., the nucleophilic and electrophilic attack zones at a given point, with the negative sign indicating the electrophilic attack zone and the positive sign indicating the nucleophilic attack zone. The Fukui functions of the studied molecules are tabulated in Tables 8 and 9. As seen in the tables, the maximum nucleophilic attack zones for oxybenzone are on C5 and C16 atoms, while the maximum electrophilic attack zones are on C4 and C11 atoms. These attack zones are on C9 and C12, and on O2 and C6 for avobenzone, respectively. For the octinoxate molecule, on C14 and C12, and on O3 and C15, respectively. For the padimate molecule, on O2 and C14, and on O3 and C13, respectively.

**Table 8:** Fukui Functions (*f*^+^(*r*), *f*^–^(*r*), *f*^0^(*r*)) for Oxybenzone (a) and Avobenzone (b)

a					b				
atom	Δ*f*(*r*)	*f*^+^(*r*)	*f*^–^(*r*)	*f*^0^(*r*)	atom	Δ*f*(*r*)	*f*^+^(*r*)	*f*^–^(*r*)	*f*^0^(*r*)
O1	–0.0417	0.0280	0.0697	0.0489	C1	–0.0055	0.0117	0.0172	0.0144
O2	–0.0354	0.0124	0.0478	0.0301	O2	–0.0368	0.0274	0.0642	0.0458
O3	0.0102	0.1245	0.1143	0.1194	C3	0.0080	0.0499	0.0419	0.0459
C4	–0.0684	0.0111	0.0795	0.0453	C4	–0.0189	0.0289	0.0477	0.0383
C5	0.0859	0.1116	0.0257	0.0686	C5	0.0072	0.0290	0.0218	0.0254
C6	0.0252	0.0241	–0.0011	0.0115	C6	–0.0303	0.0190	0.0493	0.0341
C7	–0.0150	0.0222	0.0373	0.0298	C7	0.0083	0.0370	0.0287	0.0328
C8	0.0101	0.0406	0.0306	0.0356	C8	–0.0101	0.0266	0.0367	0.0317
C9	–0.0019	0.0519	0.0539	0.0529	C9	0.0507	0.0649	0.0142	0.0395
C10	–0.0031	0.0303	0.0335	0.0319	O10	0.0246	0.0703	0.0457	0.0580
C11	–0.0426	0.0292	0.0718	0.0505	C11	0.0041	0.0166	0.0125	0.0145
C12	0.0128	0.0434	0.0306	0.0370	C12	0.0391	0.0560	0.0169	0.0364
C13	0.0237	0.0381	0.0144	0.0262	O13	0.0060	0.0782	0.0722	0.0752
C14	0.0046	0.0393	0.0348	0.0370	C14	–0.0227	0.0171	0.0398	0.0285
C15	0.0009	0.0392	0.0383	0.0387	C15	0.0051	0.0303	0.0251	0.0277
C16	0.0366	0.0735	0.0369	0.0552	C16	–0.0029	0.0279	0.0308	0.0293
C17	–0.0061	0.0127	0.0188	0.0157	C17	–0.0025	0.0478	0.0503	0.0490
H18	–0.0037	0.0208	0.0245	0.0226	C18	–0.0089	0.0242	0.0331	0.0286
H19	–0.0033	0.0245	0.0279	0.0262	C19	0.0054	0.0243	0.0189	0.0216
H20	–0.0098	0.0225	0.0323	0.0274	C20	–0.0009	0.0027	0.0036	0.0031
H21	0.0082	0.0238	0.0156	0.0197	C21	–0.0027	0.0065	0.0092	0.0079
H22	0.0131	0.0189	0.0059	0.0124	C22	–0.0030	0.0067	0.0098	0.0082
H23	0.0065	0.0296	0.0231	0.0264	C23	–0.0002	0.0040	0.0042	0.0041
H24	0.0068	0.0288	0.0219	0.0253	H24	–0.0015	0.0041	0.0056	0.0049
H25	0.0139	0.0376	0.0237	0.0307	H25	–0.0013	0.0046	0.0059	0.0052
H26	–0.0059	0.0193	0.0252	0.0223	H26	–0.0007	0.0156	0.0163	0.0160
H27	–0.0059	0.0180	0.0239	0.0209	H27	–0.0007	0.0042	0.0049	0.0046
H28	–0.0073	0.0124	0.0197	0.0160	H28	–0.0008	0.0033	0.0041	0.0037
H29	–0.0080	0.0116	0.0196	0.0156	H29	0.0001	0.0134	0.0133	0.0134
					H30	–0.0010	0.0155	0.0165	0.0160
					H31	–0.0013	0.0048	0.0061	0.0055
					H32	–0.0013	0.0053	0.0066	0.0059
					H33	0.0109	0.0253	0.0144	0.0198
					H34	0.0069	0.0189	0.0120	0.0155
					H35	–0.0019	0.0196	0.0215	0.0205
					H36	0.0006	0.0187	0.0181	0.0184
					H37	–0.0030	0.0222	0.0251	0.0237
					H38	–0.0005	0.0159	0.0163	0.0161
					H39	0.0010	0.0179	0.0169	0.0174
					H40	–0.0001	0.0094	0.0096	0.0095
					H41	–0.0010	0.0167	0.0177	0.0172
					H42	0.0002	0.0197	0.0195	0.0196
					H43	–0.0071	0.0112	0.0183	0.0147
					H44	–0.0070	0.0107	0.0177	0.0142
					H45	–0.0033	0.0163	0.0196	0.0179

**Table 9:** Fukui Functions (*f*^+^(*r*), *f*^–^(*r*), *f*^0^(*r*)) for Octinoxate (c) and Padimate O (d)

c					d				
atom	Δ*f*(*r*)	*f*^+^(*r*)	*f*^–^(*r*)	*f*^0^(*r*)	atom	Δ*f*(*r*)	*f*^+^(*r*)	*f*^–^(*r*)	*f*^0^(*r*)
O1	0.0101	0.0323	0.0223	0.0273	O1	0.0190	0.0362	0.0171	0.0267
O2	0.0298	0.0836	0.0538	0.0687	O2	0.0554	0.1115	0.0562	0.0839
O3	–0.0475	0.0349	0.0823	0.0586	N3	–0.0945	0.0325	0.1271	0.0798
C4	0.0004	0.0010	0.0007	0.0008	C4	0.0009	0.0011	0.0002	0.0007
C5	0.0006	0.0029	0.0024	0.0027	C5	0.0009	0.0034	0.0025	0.0029
C6	–0.0003	–0.0003	0.0000	–0.0001	C6	0.0003	–0.0002	–0.0006	–0.0004
C7	0.0000	0.0000	0.0001	0.0001	C7	0.0002	0.0002	0.0000	0.0001
C8	0.0022	0.0097	0.0075	0.0086	C8	0.0036	0.0105	0.0069	0.0087
C9	0.0003	0.0023	0.0019	0.0021	C9	0.0006	0.0026	0.0020	0.0023
C10	0.0009	0.0045	0.0036	0.0040	C10	0.0017	0.0049	0.0033	0.0041
C11	0.0004	0.0026	0.0023	0.0024	C11	0.0007	0.0030	0.0024	0.0027
C12	0.0508	0.0681	0.0173	0.0427	C12	0.0847	0.1014	0.0167	0.0590
C13	–0.0192	0.0914	0.1106	0.1010	C13	–0.0368	0.0550	0.0918	0.0734
C14	0.0611	0.0986	0.0374	0.0680	C14	0.0474	0.0797	0.0323	0.0560
C15	–0.0409	0.0289	0.0698	0.0494	C15	0.0243	0.0646	0.0402	0.0524
C16	0.0073	0.0458	0.0385	0.0422	C16	0.0206	0.0592	0.0386	0.0489
C17	–0.0045	0.0480	0.0525	0.0503	C17	–0.0262	0.0385	0.0647	0.0516
C18	–0.0189	0.0353	0.0542	0.0448	C18	–0.0241	0.0392	0.0633	0.0512
C19	–0.0188	0.0356	0.0544	0.0450	C19	–0.0127	0.0162	0.0289	0.0226
C20	0.0013	0.0668	0.0655	0.0662	C20	–0.0125	0.0164	0.0289	0.0226
C21	–0.0069	0.0149	0.0218	0.0184	C21	0.0022	0.0029	0.0007	0.0018
H22	0.0010	0.0028	0.0018	0.0023	H22	0.0009	0.0045	0.0036	0.0041
H23	0.0006	0.0038	0.0032	0.0035	H23	0.0027	0.0097	0.0070	0.0084
H24	0.0017	0.0087	0.0070	0.0079	H24	0.0013	0.0063	0.0050	0.0056
H25	0.0008	0.0051	0.0043	0.0047	H25	0.0005	–0.0083	–0.0088	–0.0085
H26	–0.0004	–0.0071	–0.0067	–0.0069	H26	0.0011	0.0021	0.0011	0.0016
H27	0.0005	0.0019	0.0014	0.0016	H27	–0.0004	–0.0026	–0.0022	–0.0024
H28	–0.0004	–0.0029	–0.0025	–0.0027	H28	0.0038	0.0130	0.0092	0.0111
H29	0.0020	0.0117	0.0097	0.0107	H29	0.0038	0.0127	0.0088	0.0108
H30	0.0015	0.0102	0.0086	0.0094	H30	0.0005	0.0029	0.0025	0.0027
H31	0.0003	0.0025	0.0022	0.0024	H31	0.0012	0.0052	0.0040	0.0046
H32	0.0007	0.0048	0.0041	0.0044	H32	0.0027	0.0080	0.0053	0.0067
H33	0.0015	0.0071	0.0056	0.0063	H33	0.0017	0.0022	0.0006	0.0014
H34	0.0008	0.0024	0.0016	0.0020	H34	0.0017	0.0094	0.0077	0.0085
H35	0.0011	0.0080	0.0068	0.0074	H35	0.0015	0.0076	0.0061	0.0069
H36	0.0010	0.0068	0.0058	0.0063	H36	0.0002	0.0007	0.0005	0.0006
H37	0.0001	0.0003	0.0002	0.0003	H37	0.0009	0.0027	0.0019	0.0023
H38	0.0005	0.0024	0.0019	0.0022	H38	0.0047	0.0339	0.0292	0.0316
H39	0.0056	0.0378	0.0322	0.0350	H39	0.0034	0.0318	0.0285	0.0301
H40	0.0122	0.0353	0.0231	0.0292	H40	–0.0021	0.0280	0.0301	0.0290
H41	–0.0016	0.0232	0.0248	0.0240	H41	–0.0017	0.0281	0.0298	0.0289
H42	–0.0049	0.0261	0.0310	0.0286	H42	–0.0170	0.0179	0.0349	0.0264
H43	–0.0049	0.0277	0.0326	0.0302	H43	–0.0068	0.0220	0.0288	0.0254
H44	–0.0051	0.0254	0.0305	0.0279	H44	–0.0185	0.0215	0.0399	0.0307
H45	–0.0053	0.0203	0.0256	0.0230	H45	–0.0067	0.0220	0.0287	0.0254
H46	–0.0087	0.0143	0.0230	0.0187	H46	–0.0169	0.0183	0.0352	0.0267
H47	–0.0087	0.0143	0.0231	0.0187	H47	–0.0182	0.0215	0.0396	0.0305

### 3.8 ELF Analysis

**Figure 7:**
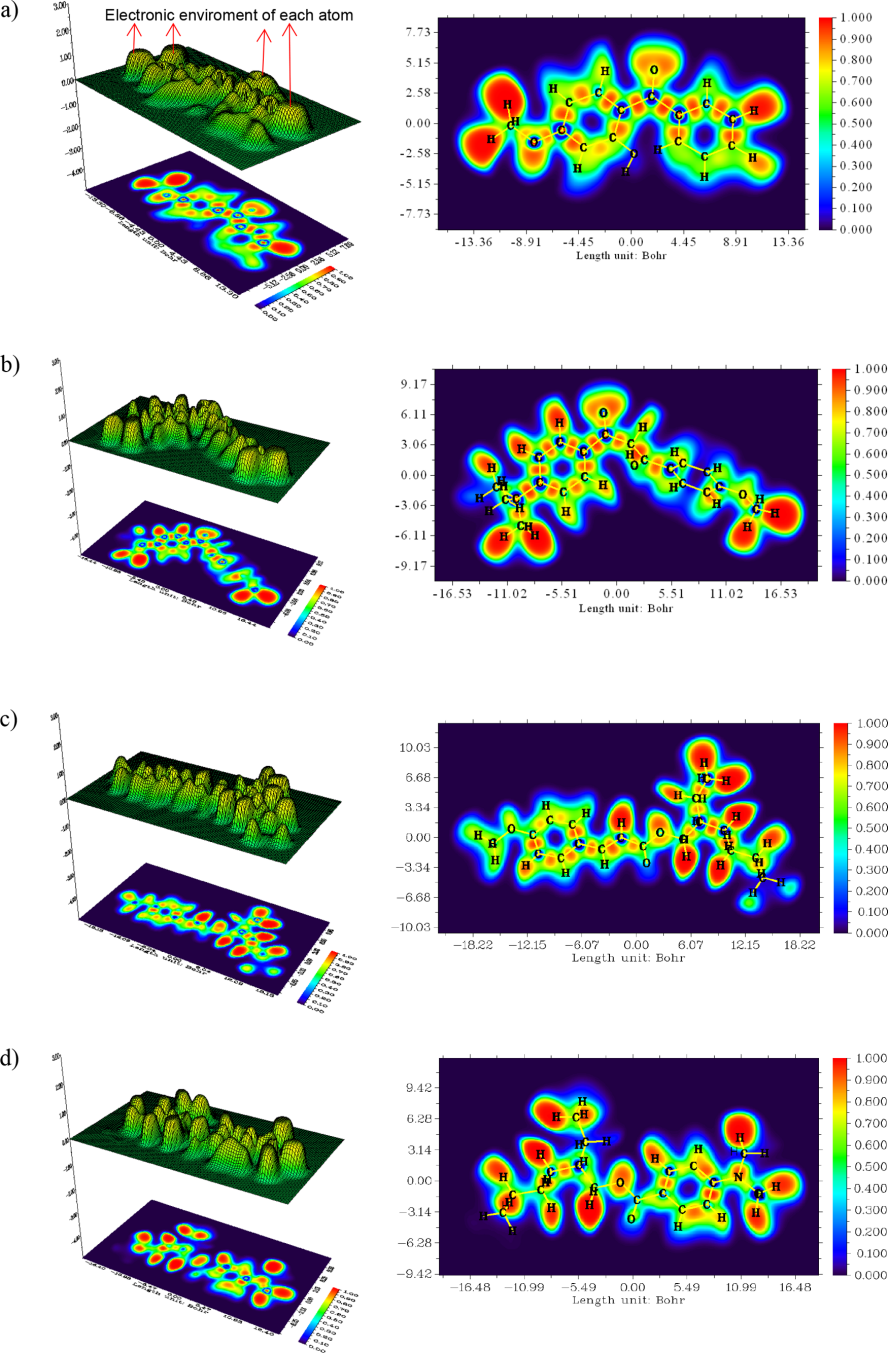
ELF projection maps filled with color for oxybenzone (a), avobenzone (b), octinoxate (c), and padimate O (d).

ELF, derived from kinetic energy density, is used for detailed chemical mapping to indicate chemically important regions such as lone pairs or bond pairs and to understand chemical bonds.^70^ ELF maps of the molecules studied were simulated using the Multiwfn program. The color-filled projection maps of ELF for organic UV filters such as oxybenzone, avobenzone, octinoxate, and padimate O are shown in Figure 7. While red color corresponds to high values of ELF, blue color creates the region where ELF value is low and shows the high ELF regions mostly around hydrogen atoms, and the delocalized electron cloud density surrounding the carbon and oxygen atoms is illustrated by blue color. That is, blue and especially dark blue areas revealed a decrease in the ELF. ELF analysis has also revealed the presence of an electronic lone pair in the vicinity of the attractive −C=O groups found in organic UV filters. This finding improves our understanding of the molecular interactions involved in these compounds.

### 3.9 Molecular Docking

**Figure 8:**
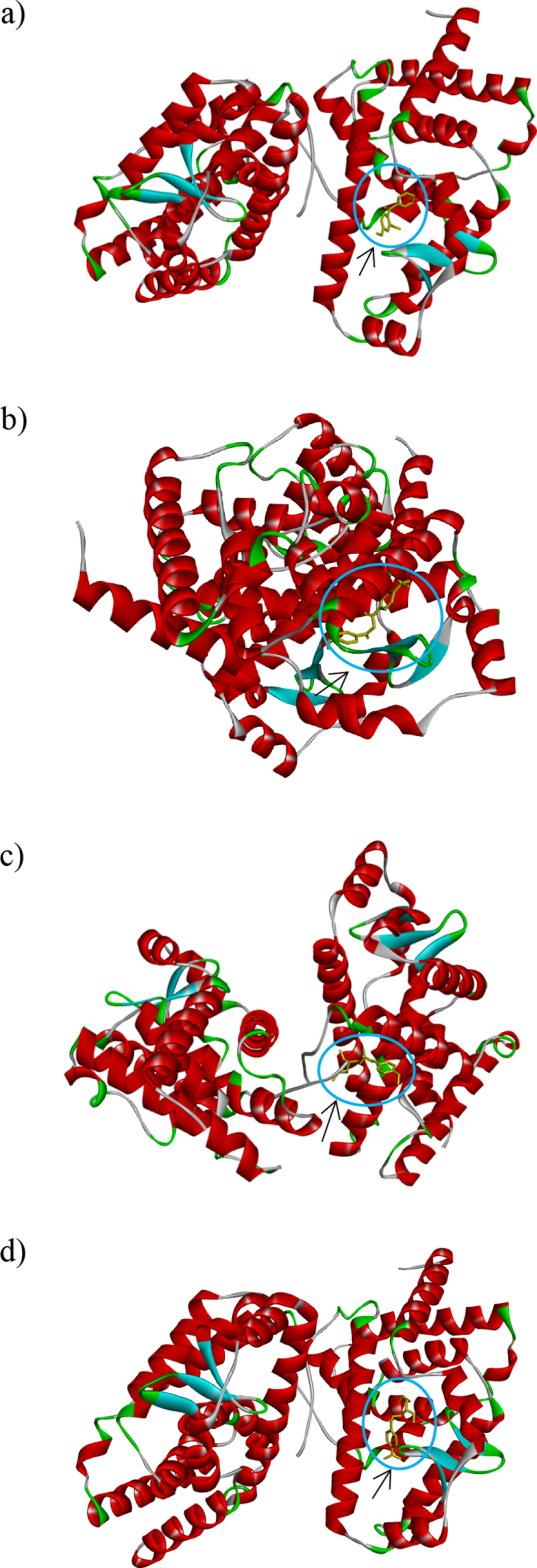
Molecular docking (best docked post) of oxybenzone (a), avobenzone (b), octinoxate (c), and padimate (d) with the retinoic acid-related orphan receptor γ(t) (RORγ(t)).

**Figure 9:**
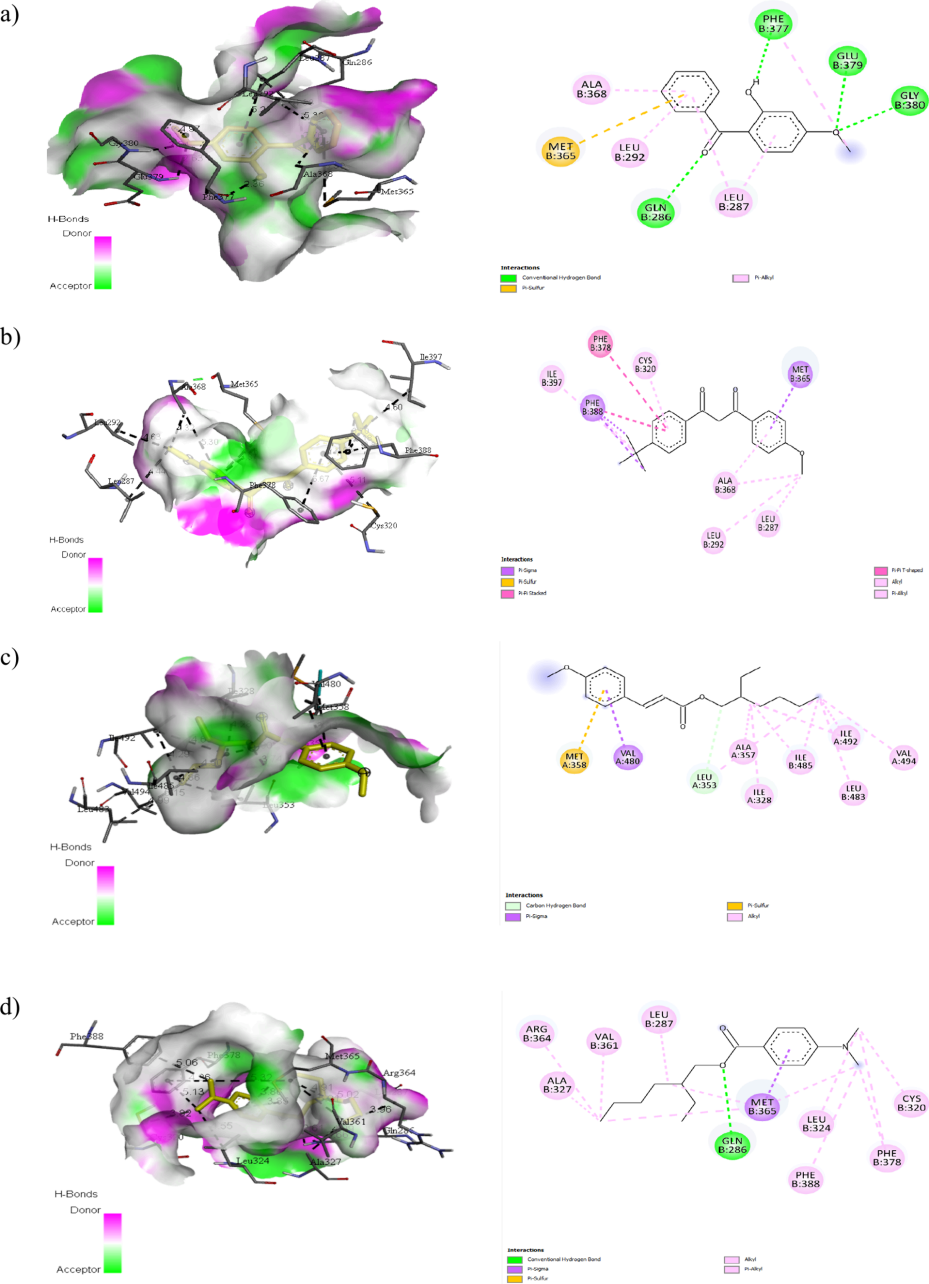
H-bond interaction surfaces (left) and 2D receptor–ligand interaction diagram (right) of oxybenzone (a), avobenzone (b), octinoxate (c), and padimate (d) with the retinoic acid-related orphan receptor γ(t) (RORγ(t)).

Due to drug developments' high cost and lengthy development time, molecular docking is used as an alternative technique to investigate binding interactions between ligands and receptors and identify the therapeutic potential of chemical substances.^41,71,72^ The transcription factor RORγt plays a crucial role in TH17 cell differentiation and IL-17/22 production, making it a key target for treating inflammatory diseases. RORγt facilitates the development of secondary lymphoid organs and regulates thymic T cell development. Its strong link to autoimmunity emphasizes its importance for drug development, as inhibiting RORγt may benefit various inflammatory and autoimmune disorders. Dysfunction of RORγ(t) (retinoic acid-related orphan receptor γt), which plays a role in regulating the immune system, has been associated with inflammatory and autoimmune diseases as well as skin cancer. Activation of RORγ(t) in the skin, resulting from exposure to environmental chemicals, can be linked to the promotion of inflammatory skin diseases.^22^ UV filters, commonly used as additives in cosmetics and body-care products, have raised concerns regarding their endocrine-disrupting properties. Limited information is available about the potential immune-disrupting effects of UV-filtering chemicals. Additionally, the activation of RORγ by xenobiotics may interfere with the immune responses. Therefore, this study focuses on the docking interactions between these filters and the RORγ(t) receptor. Here, molecular docking analysis was used to investigate the binding interaction of organic filters such as oxybenzone, avobenzone, octinoxate, and padimate O with the retinoic acid-related orphan receptor γ(t) (RORγ(t)). The RORγ(t) receptor was identified in the literature and subsequently acquired in PDB format from the RCSB Protein Data Bank (https://www.rcsb.org/). Oxybenzone, avobenzone, octinoxate, and padimate O were docked to the active domains of the 5ZA1 receptor (Human Nuclear receptor RORγ). Auto-Dock Vina version 1.5.7 and Discovery Studio Visualizer programs are useful tools that provide insight into the mechanisms of ligand-protein interactions and binding to receptors with a known 3D structure. A molecular docking study was conducted with these two valuable programs, and the molecular docking results are given in Figures 8 and 9.

The data are listed in Table 10. As seen in Figure 9, there were notable conventional hydrogen bond interactions of oxybenzone and padimate O with GLN286B. Oxybenzone was found to have conventional hydrogen bond interactions indicated by the light green line with GLN286B, PHE377B, GLU379B, and GLY380B. Table 10 shows the receptor–ligand interactions, and it can be observed that avobenzone has the lowest expected binding free energy of −8.6 kcal/mol. Moreover, the electrophilicity index can be effectively used as an important descriptor for evaluating the biological activity properties of various compounds, indicating that it can provide information on their potential interactions and effects within biological systems.^72,73^ According to the binding affinity results presented in Table 10, it can be said that the global electrophilicity index values shown in Table 1 are in agreement.^72,73^

**Table 10:** Molecular Docking Scores of RORγt Protein (5ZA1) and Ligand Oxybenzone (a), Avobenzone (b), Octinoxate (c), and Padimate O (d)

					distance from best mode							
	affinity (kcal/mol)				RMSD lower bound				RMSD upper bound			
mode	a	b	c	d	a	b	c	d	a	b	c	d
1	–7.3	–8.6	–7.6	–6.7	0.000	0.000	0.000	0.000	0.000	0.000	0.000	0.000
2	–7.2	–8.4	–7.4	–6.4	1.707	3.479	0.836	5.091	5.957	7.500	2.790	8.517
3	–6.9	–7.6	–7.2	–6.3	2.600	15.317	25.831	2.161	3.497	17.002	27.565	3.535
4	–6.9	–7.5	–7.2	–6.3	23.116	14.661	24.977	24.731	25.637	16.115	27.363	26.798
5	–6.8	–7.4	–7.1	–6.1	3.318	15.695	26.477	15.041	6.387	18.175	28.348	17.369
6	–6.8	–7.4	–7.1	–6.1	15.803	3.020	24.879	13.483	17.483	7.946	27.350	16.608
7	–6.7	–7.4	–7.0	–6.0	8.074	15.238	25.504	14.993	10.539	17.559	27.045	17.595
8	–6.7	–7.4	–6.6	–6.0	12.531	14.963	15.091	3.889	13.863	17.852	17.119	5.171
9	–6.7	–7.3	–6.5	–6.0	2.909	15.089	26.235	15.128	4.253	16.534	28.246	18.186

### 3.10 ADME Properties

**Figure 10:**
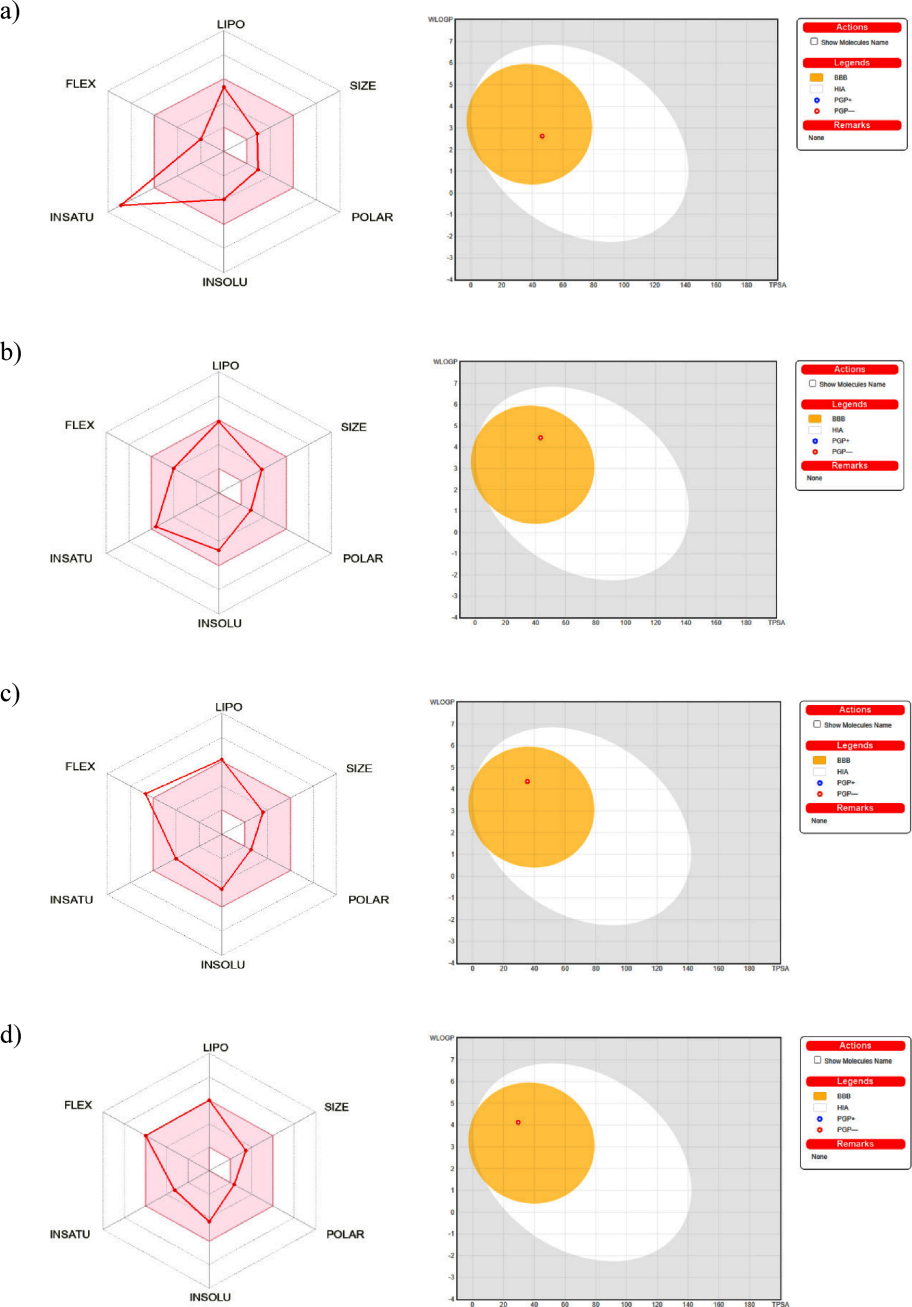
Bioavailability radar (left) and BOILED-Egg (right) for oxybenzone (a), avobenzone (b), octinoxate (c), and padimate O (d).

Research indicates that the antagonistic response of an inhibitor to an enzyme or protein receptor does not automatically imply its effectiveness as a potential medication. Therefore, to facilitate informed decision-making regarding the use of inhibitors within biological systems, it is essential to conduct ADME studies. These studies, along with drug-likeness analysis, play a vital role in the drug development process, ensuring a comprehensive understanding of how inhibitors may interact within the body.^74−76^ The prediction of ADME properties can reduce the risk of disadvantage during clinical evaluation. ADME characteristics such as physiological and pharmacokinetic parameters are calculated by the SwissADME web server, and the results are tabulated in Tables 11 and 12. Based on Lipinski’s rule of five, we examined that organic filters should have the following five features; (a) number of hydrogen bond donors (HBDs) donors <5, (b) number of hydrogen bond acceptors (HBAs) < 10, (c) Log *P* < 5, (d) number of rotatable bonds <10 and, (e) molecular weight <500 Da.^77^ The findings show that none of the calculated organic filters violate any of the established criteria. Thus, these molecules perfectly comply with the Lipinski rule. In order to evaluate the impact of polar fragmentation on the surface of a chemical structure, the topological polar surface area (TPSA) is often used. It is recommended that the TPSA value of a compound should not exceed 140 Å^2^ as a higher value may hinder blood-brain barrier penetration and lead to a decrease in membrane permeability. The TPSA values of the organic filters studied were found to be within the specified range, i.e., less than 140 Å^2^. Drug transport properties such as blood-brain barrier (BBB) penetration^75^ and intestinal absorption (HIA) of the organic filters studied with the BOILED-Egg model provided by the SwissADME web server are shown in Figure 10. The SwissADME web server also enables the determination of whether a chemical is in the substrate (PGP+) and nonsubstrate (PGP-) parameters.^78^ According to the results, the red dots (PGP-) seen in all organic filters are for molecules expected to be excreted from the central nervous system by P-glycoprotein. Moreover, the percentage of absorption estimated for oxybenzone, avobenzone, octinoxate, and padimate O suggests that these molecules can be easily absorbed by humans. In conclusion, predicting the potential hazards of UV filters using molecular modeling has significant benefits for both our health and the environment. By applying advanced computer techniques to study the structure and behavior of these substances, we can better understand their risks and find ways to reduce exposure. This approach helps protect people from harmful chemicals while preserving ecosystems, leading to a healthier planet for future generations.

**Table 11:** ADME Properties Obtained by SwissADME of Oxybenzone (a) and Avobenzone (b)^a^

physicochemical properties			pharmacokinetics			water solubility		
	a	b		a	b		a	b
F	C_14_H_12_O_3_	C_20_H_22_O_3_	GI	high	high	Log *S* (ESOL)	–3.97	–4.75
MW	228.24 g/mol	310.39 g/mol	BBB	yes	yes	S	2.46e-02 mg/mL; 1.08e-04 mol/L	5.48e-03 mg/mL; 1.76e-05 mol/L
nHA	17	23	P-gp	no	no	C	soluble	moderately soluble
nAHA	12	12	CYP1A2 inh.	yes	yes	Log *S* (Ali)	–4.46	–5.40
fraction Csp3	0.07	0.30	CYP2C19 inh.	yes	yes	S	7.89e-03 mg/mL; 3.46e-05 mol/L	1.23e-03 mg/mL; 3.97e-06 mol/L
nRB	3	6	CYP2C9 inh.	yes	no	C	moderately soluble	moderately soluble
nHBA	3	3	CYP2D6 inh.	no	Yes	Log *S* (SILICOS-IT)	–4.42	–6.54
nHBD	1	0	CYP3A4 inh.	no	yes	S	8.62e-03 mg/mL; 3.78e-05 mol/L	8.99e-05 mg/mL; 2.90e-07 mol/L
MR	64.83	92.11	Log *K*_p_ (skin perm.)	–5.00 cm/s	–4.81 cm/s	C	moderately soluble	poorly soluble
TPSA	46.53 Å^2^	43.37 Å^2^						

lipophilicity			druglikeness			medicinal chemistry		
	a	b		a	b		a	b
Log *P*_o/w_ (iLOGP)	2.43	3.00	Lipinski	yes; 0 violation	yes; 0 violation	PAINS	0 alert	0 alert
Log *P*_o/w_ (XLOGP3)	3.79	4.76	Ghose	yes	yes	Brenk	0 alert	1 alert: beta_keto_anhydride
Log *P*_o/w_ (WLOGP)	2.63	4.45	Veber	yes	yes	leadlikeness	no; 2 violations: MW < 250. XLOGP3 > 3.5	no; 1 violation: XLOGP3 > 3.5
Log *P*_o/w_ (MLOGP)	1.99	3.09	Egan	yes	yes	SA	1.80	2.00
Log *P*_o/w_ (SILICOS-IT)	2.92	5.05	Muegge	yes	yes			
consensus Log *P*_o/w_	2.75	4.07	BS	0.55	0.55			

a SA: Synthetic accessibility; nHBA: Num. H-bond acceptors; nHBD: Num. H-bond donors; nRB: Num. rotatable bonds; MR: Molar Refractivity; BS: Bioavailability Score; MW: Molecular weight; F: Formula; nHA: Num. heavy atoms; nAHA: Num. arom. heavy atoms; GI: gastrointestinal absorption; BBB: blood–brain barrier permeant; P-gp: P-glycoprotein substrate; S: Solubility; TPSA: topological polar surface area; C: Class; MLogP: Moriguchi partition coefficient.

**Table 12:** ADME Properties Obtained By SwissADME of Octinoxate (c) and Padimate O (d)

physicochemical properties			pharmacokinetics			water solubility		
	c	d		c	d		c	d
F	C_18_H_26_O_3_	C_17_H_27_NO_2_	GI	high	high	Log *S* (ESOL)	–4.53	–4.36
MW	290.40 g/mol	277.40 g/mol	BBB	yes	yes	S	8.55e-03 mg/mL; 2.95e-05 mol/L	1.22e-02 mg/mL; 4.40e-05 mol/L
nHA	21	20	P-gp	no	no	C	moderately soluble	moderately soluble
nAHA	6	6	CYP1A2 inh.	yes	Yes	Log *S* (Ali)	–5.80	–5.39
fraction Csp3	0.50	0.59	CYP2C19 inh.	yes	no	S	4.63e-04 mg/mL; 1.60e-06 mol/L	1.13e-03 mg/mL; 4.06e-06 mol/L
nRB	10	9	CYP2C9 inh.	yes	no	C	moderately soluble	moderately soluble
nHBA	3	2	CYP2D6 inh.	yes	yes	Log *S* (SILICOS-IT)	–5.14	–5.04
nHBD	0	0	CYP3A4 inh.	no	no	S	2.08e-03 mg/mL; 7.18e-06 mol/L	2.52e-03 mg/mL; 9.08e-06 mol/L
MR	87.57	85.58	Log *K*_p_ (skin perm.)	–4.31 cm/s	–4.42 cm/s	C	moderately soluble	moderately soluble
TPSA	35.53 Å^2^	29.54 Å^2^						

lipophilicity			druglikeness			medicinal chemistry		
	c	d		c	d		c	d
Log *P*_o/w_ (iLOGP)	4.14	4.01	Lipinski	yes; 0 violation	yes; 0 violation	PAINS	0 alert	0 alert
Log *P*_o/w_ (XLOGP3)	5.30	5.03	Ghose	yes	yes	Brenk	1 alert: michael_acceptor_1	0 alert
Log *P*_o/w_ (WLOGP)	4.36	4.13	Veber	yes	yes	leadlikeness	no; 2 violations: Rotors >7. XLOGP3 > 3.5	no; 2 violations: Rotors >7. XLOGP3 > 3.5
Log *P*_o/w_ (MLOGP)	3.68	3.80	Egan	yes	Yes	SA	3.31	2.79
Log *P*_o/w_ (SILICOS-IT)	4.87	3.88	Muegge	no; 1 violation: XLOGP3 > 5	no; 1 violation: XLOGP3 > 5			
consensus Log *P*_o/w_	4.47	4.17	BS	0.55	0.55			

### 3.11 Hirshfeld Surface Analysis

**Figure 11:**
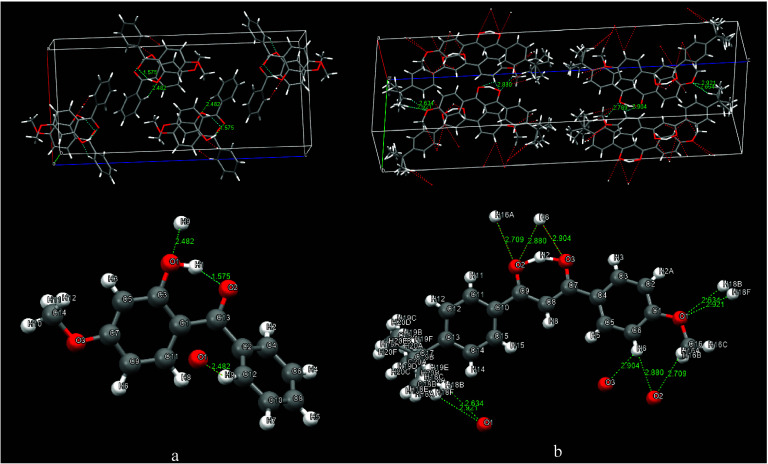
Crystal packing diagram of oxybenzone (a) and avobenzone (b).

The molecular crystals, such as oxybenzone and avobenzone, are categorized under the *Pbca* space group. Figure 11 presents a crystal packing diagram that delineates both intra- and intermolecular interactions, specifically, O–H···O and C–H···O interactions. The distances for these interactions are as follows: 1.575 Å (O1–H1···O2), 2.482 Å (H1–O1···H9), Å for A; 2.880 Å (C6–H6···O2), 2.904 Å (C6–H6···O3), 2.709 Å (C16–H16A···O2), 2.634 Å (C18–H18B···O1), 2.921 Å (C18–H18F···O1) for b. A Hirshfeld surface analysis was executed to investigate the intermolecular contact points and interactions between donor and acceptor groups in the crystal packing of oxybenzone and avobenzone.

**Figure 12:**
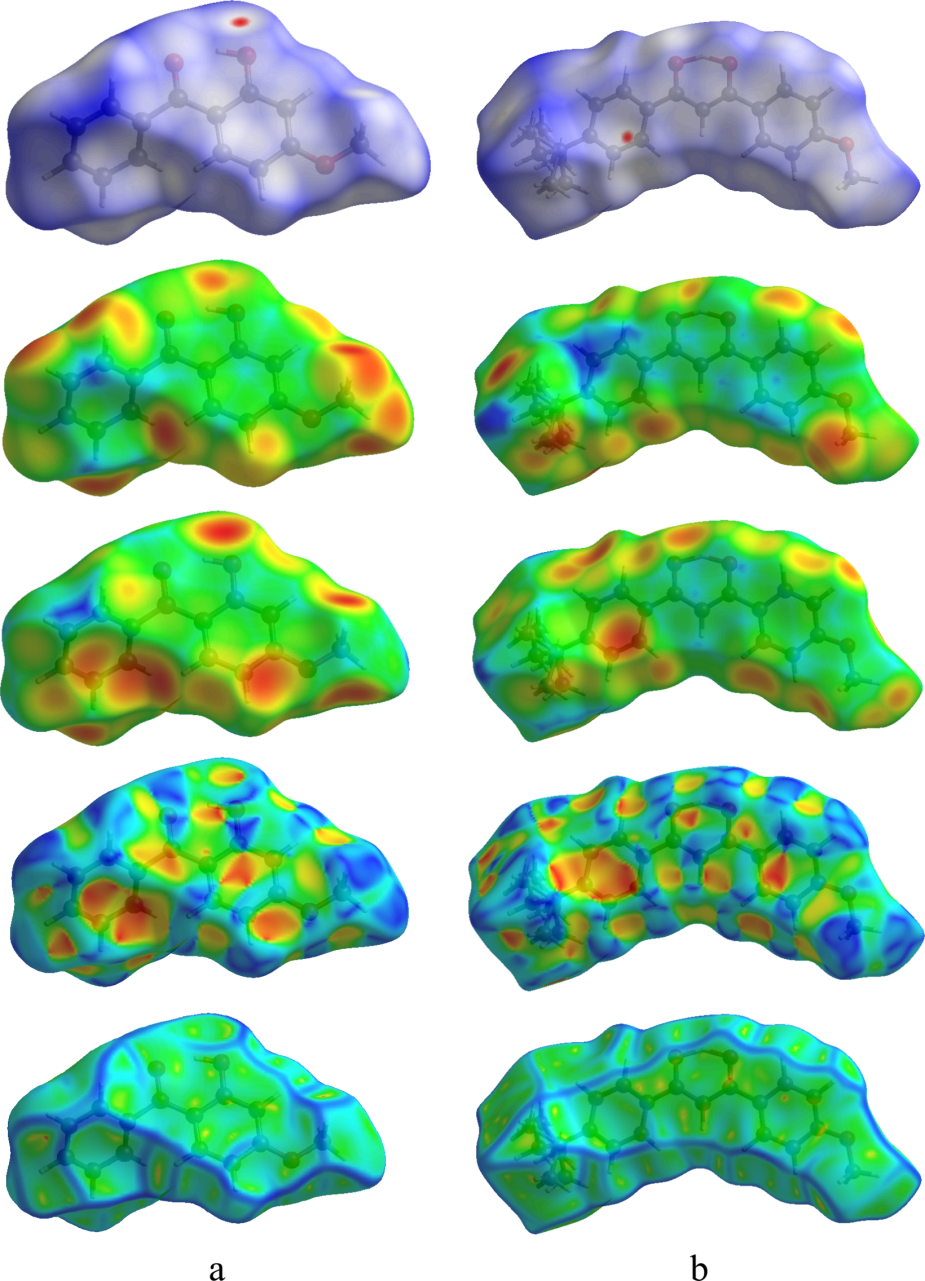
3D Hirsfeld surface of oxybenzone (a) and avobenzone (b) as the dnorm, di, de, shape index, and curvedness index, respectively.

The CrystalExplorer 17.5 software package was utilized to generate 3D surfaces for dnorm, di, de, shape index, and curvedness as well as their corresponding 2D fingerprint histograms. The three-dimensional mapping of normalized contact distance (dnorm), di, de, shape index, and curvedness index for oxybenzone and avobenzone was presented in Figure 12, respectively. As per Figure 12, the H-bonding contacts are denoted by the deep red impressions visible on the dnorm surfaces.^79^ The presence of blue and red triangles in the shape index is evidence for C–H−π and π–π interactions.^80^

**Figure 13:**
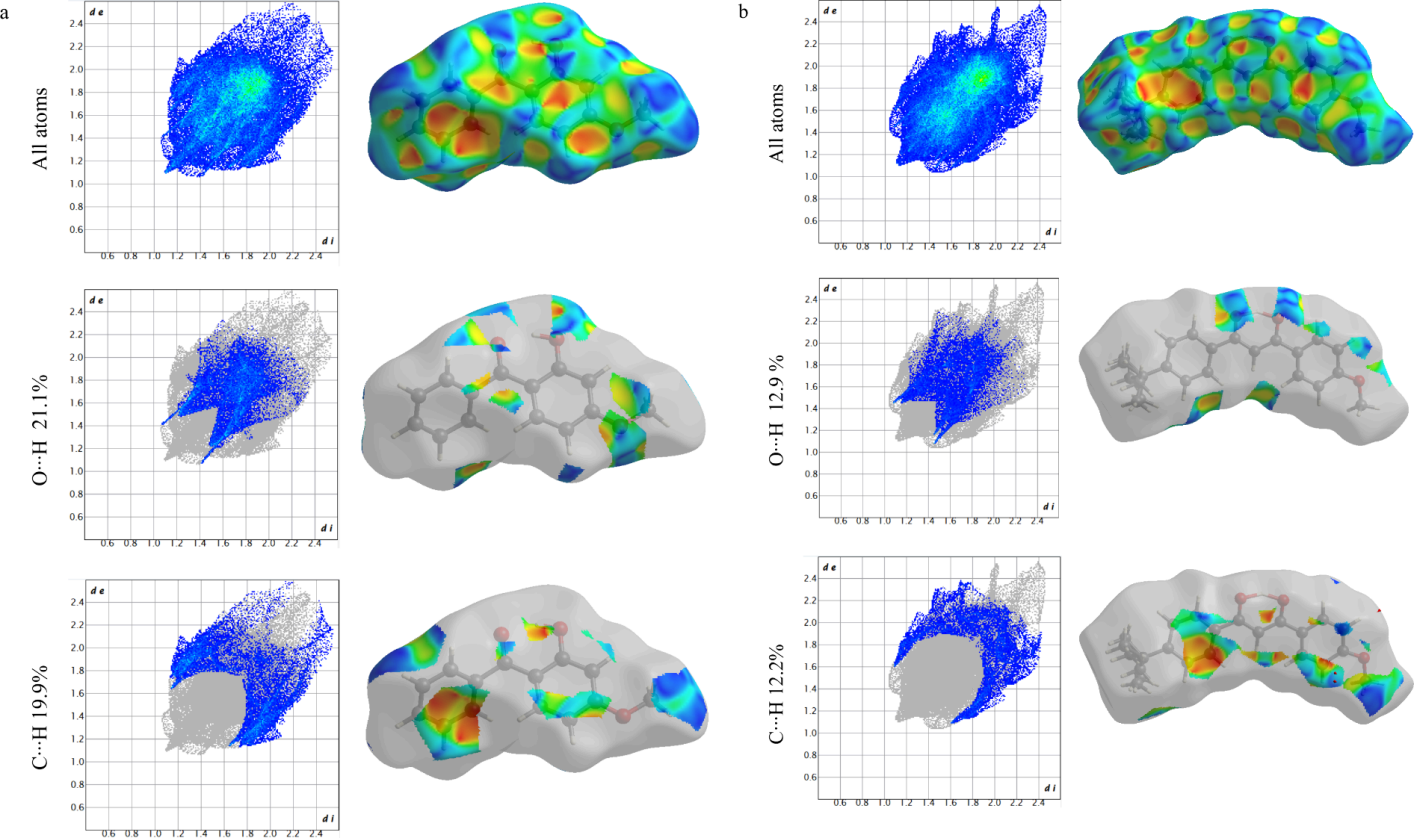
Fingerprint plots (hirshfeld surface plotted on the shape index and quantitative noncovalent interactions) for oxybenzone (a) and avobenzone (b).

The inherent properties facilitate the investigation and exploration of short atom–atom contacts, enabling an assessment of their potential for π-stacking and hydrogen bonding interactions.^81^ However, this study primarily focused on the O···H interactions and C atom (C–H···O) interactions. The 2-D fingerprint plot,^82^ illustrated in Figure 13, offers quantitative insights into the various intermolecular interactions present within the crystal structure. This figure comprehensively represents all noncovalent contacts, accounting for 100% of the interactions observed. The analysis of the Hirshfeld surface (HS) for oxybenzone and avobenzone demonstrates distinct patterns that play a significant role in their crystalline stability^83^ and are mainly stabilized by the O···H and C···H interactions, respectively. The O···H and C···H interactions for oxybenzone (a) and avobenzone (b) account for for 21.1 and 19.9%, and 12.9 and 12.2%, respectively. The analysis of Hirshfeld surfaces further reveals the presence of additional weak intermolecular interactions.^84^

## 4 Conclusions

This study presents a theoretical study of some organic UV filters, such as oxybenzone, avobenzone, octinoxate, and padimate O. In the first part of the study, the theoretically optimized geometries of organic filters containing UV-sensitive groups were determined by DFT using the B3LYP/6-31G(d,p) basis set. FMOs and some chemical parameters were determined by using the same method. According to the HOMO–LUMO study, the octinoxate molecule has higher chemical reactivity than other molecules, with an energy of 4.34 eV in both gas and solution environments. The oscillator strengths, absorption wavelengths, and excitation energies in gas, water, ethanol, and *n*-hexane phases will be determined using the TD-DFT with the CPCM solvent model to understand the effect of solvents on the chemical parameters, electronic properties, and reactivity of organic filters. The wavelengths of greatest absorption for oxybenzone, avobenzone, octinoxate, and padimate O are observed in *n*-hexane (309.04 nm), water (291.43 nm), ethanol (311.47 nm), and water (292.37 nm), respectively. NBO and NLMO analyses were conducted to determine charge transfer or conjugative interactions. The stability and intramolecular interactions were interpreted by NBO/NLMO/AIM, Hirsfeld and RDG/NCI analysis, and the processes that stabilize the structure were determined by second-order perturbation energy calculations. MEP and Fukui function analyses were conducted, and it was seen that the electrophilicity reactivity was mostly on oxygen atoms. Finally, molecular docking, ADME, and topological properties were conducted. According to the prediction of the bioactivity score, the organic filters exhibit bioactivity. It is believed that this study will lead to a better understanding of organic filter chemistry in a shorter period of time, opening up new horizons.

## Data Availability

The data is available throughout the manuscript and supporting files.

## Supporting Information Available

Additional detail tables for all compounds (PDF)
