# Supplementary material for: Quantum Chemical Calculations, Topological Properties, ADME/Molecular Docking Studies, and Hirshfeld Surface Analysis on Some Organic UV-Filters
Source: ACS Omega. 2025 Apr 8;10(15):14884–907. doi: 10.1021/acsomega.4c10102 (PMC12019759; doi:10.1021/acsomega.4c10102)
Supplement: Supplementary file 1 — ao4c10102_si_001.pdf [file ao4c10102_si_001.pdf]

Supporting Information

for

Quantum chemical calculations, topological properties, ADME/molecular docking studies, and Hirshfeld surface analysis on some organic UV-filters

[a,b]Feride Akman* and [c]Buşra Kutlu

[a,c]Vocational School of Food, Agriculture and Livestock, Bingol University, 12000 Bingöl, Turkey

[b]Chemistry Program, Institute of Sciences, Bingol University, 12000 Bingol, Turkey

---

*Corresponding Author: E–mail: ferideakman@bingol.edu.tr, chemakman@gmail.com (Bingol University, 1200, Bingol, Turkey)

Coauthor:kutlubusra752@gmail.com (Bingol University, 1200, Bingol, Turkey)

Table of Contents

Table S1. The Fock matrix of oxybenzone can be analyzed through second order perturbation theory using the Natural Bond Orbital (NBO) method.

Table S2. The Fock matrix of avobenzone can be analyzed through second order perturbation theory using the Natural Bond Orbital (NBO) method.

Table S3. The Fock matrix of octinoxate can be analyzed through second order perturbation theory using the Natural Bond Orbital (NBO) method

Table S4. The Fock matrix of padimate can be analyzed through second order perturbation theory using the Natural Bond Orbital (NBO) method.

Table S5. Oxybenzone's significant NLMO occupancy, percentage derived from its parent NBO, and atomic hybrid contributions

Table S6. Avobenzone's significant NLMO occupancy, percentage derived from its parent NBO, and atomic hybrid contributions

Table S7. Octinoxate's significant NLMO occupancy, percentage derived from its parent NBO, and atomic hybrid contributions

Table S8. Padimate O's significant NLMO occupancy, percentage derived from its parent NBO, and atomic hybrid contributions

**Table S1.** The Fock matrix of oxybenzone can be analyzed through second order perturbation theory using the Natural Bond Orbital (NBO) method.							
No	Donor (i)	Type	Acceptor (j)	Type	$E^{(2)a}$(KJ mol^{-1})	E(j)-E(i)^{b} (a.u)	F(i.j)^{C} (a.u)
1	O1-C9	σ	C7-C10	σ*	1.52	1.48	0.042
2	O1-C9	σ	C8-C11	σ*	0.97	1.5	0.034
3	O1-C9	σ	C9-C10	σ*	0.54	1.47	0.025
4	O1-C9	σ	C9-C11	σ*	0.72	1.46	0.029
5	O1-C9	σ	C17-H27	σ*	0.63	1.36	0.026
6	O1-C17	σ	C9-C10	σ*	2.84	1.38	0.056
7	O2-C7	σ	C4-C7	σ*	0.61	1.47	0.027
8	O2-C7	σ	C4-C8	σ*	1.48	1.49	0.042
9	O2-C7	σ	C7-C10	σ*	0.77	1.49	0.030
10	O2-C7	σ	C9-C10	σ*	1.36	1.48	0.040
11	O2-H26	σ	C4-C7	σ*	5.1	1.29	0.073
12	O3-C5	σ	C4-C5	σ*	1.03	1.49	0.036
13	O3-C5	σ	C4-C7	σ*	1.14	1.59	0.038
14	O3-C5	σ	C5-C6	σ*	0.98	1.49	0.035
15	O3-C5	σ	C6-C13	σ*	1.19	1.63	0.039
16	O3-C5	π	C4-C7	σ*	0.95	0.9	0.026
17	O3-C5	π	C4-C8	π*	2.63	0.39	0.032
18	O3-C5	π	C6-C13	π*	4.32	0.4	0.041
19	C4-C5	σ	O3-C5	σ*	0.54	1.24	0.023
20	C4-C5	σ	C4-C7	σ*	2.22	1.19	0.046
21	C4-C5	σ	C4-C8	σ*	2.25	1.22	0.047
22	C4-C5	σ	C5-C6	σ*	0.53	1.09	0.022
23	C4-C5	σ	C6-C12	σ*	1.82	1.22	0.042
24	C4-C5	σ	C7-C10	σ*	2.38	1.21	0.048
25	C4-C5	σ	C8-C11	σ*	2.67	1.24	0.051
26	C4-C7	σ	O2-H26	σ*	1.98	1.13	0.043

27	C4-C7	σ	O3-C5	σ*	0.99	1.29	0.032
28	C4-C7	σ	C4-C5	σ*	1.87	1.15	0.042
29	C4-C7	σ	C4-C8	σ*	3.24	1.28	0.058
30	C4-C7	σ	C7-C10	σ*	3.21	1.27	0.057
31	C4-C7	σ	C8-H18	σ*	1.93	1.19	0.043
32	C4-C7	σ	C10-H19	σ*	2.09	1.16	0.044
33	C4-C8	σ	O2-C7	σ*	3.93	1.04	0.057
34	C4-C8	σ	O3-C5	π*	0.67	0.71	0.020
35	C4-C8	σ	C4-C5	σ*	2.15	1.13	0.044
36	C4-C8	σ	C4-C7	σ*	3.33	1.23	0.057
37	C4-C8	σ	C5-C6	σ*	1.72	1.13	0.040
38	C4-C8	σ	C8-C11	σ*	2.59	1.28	0.052
39	C4-C8	σ	C8-H18	σ*	1.09	1.17	0.032
40	C4-C8	σ	C11-H20	σ*	2.22	1.16	0.046
41	C4-C8	π	O3-C5	σ*	1.66	0.84	0.036
42	C4-C8	π	O3-C5	π*	13.97	0.27	0.057
43	C4-C8	π	C4-C8	π*	1.24	0.28	0.017
44	C4-C8	π	C5-C6	σ*	1.44	0.69	0.030
45	C4-C8	π	C7-C10	π*	26.3	0.27	0.076
46	C4-C8	π	C9-C11	π*	14.84	0.27	0.057
47	C5-C6	σ	O3-C5	σ*	0.58	1.24	0.024
48	C5-C6	σ	C4-C5	σ*	0.51	1.09	0.021
49	C5-C6	σ	C4-C8	σ*	1.49	1.22	0.038
50	C5-C6	σ	C6-C12	σ*	1.85	1.22	0.042
51	C5-C6	σ	C6-C13	σ*	2.13	1.22	0.046
52	C5-C6	σ	C12-C14	σ*	2.3	1.24	0.048
53	C5-C6	σ	C13-C15	σ*	2.1	1.23	0.045
54	C6-C12	σ	C4-C5	σ*	1.92	1.12	0.042
55	C6-C12	σ	C5-C6	σ*	1.84	1.13	0.041
56	C6-C12	σ	C6-C13	σ*	3.68	1.26	0.061
57	C6-C12	σ	C12-C14	σ*	2.38	1.27	0.049
58	C6-C12	σ	C12-H21	σ*	1.13	1.18	0.033

59	C6-C12	σ	C13-H22	σ*	2.29	1.16	0.046
60	C6-C12	σ	C14-H23	σ*	2.31	1.16	0.046
61	C6-C13	σ	O3-C5	σ*	1.38	1.28	0.038
62	C6-C13	σ	C5-C6	σ*	1.73	1.13	0.040
63	C6-C13	σ	C6-C12	σ*	3.74	1.26	0.061
64	C6-C13	σ	C12-H21	σ*	1.96	1.18	0.043
65	C6-C13	σ	C13-C15	σ*	2.62	1.27	0.052
66	C6-C13	σ	C13-H22	σ*	1.22	1.17	0.034
67	C6-C13	σ	C15-H24	σ*	2.19	1.17	0.045
68	C6-C13	π	O3-C5	σ*	0.58	0.83	0.022
69	C6-C13	π	O3-C5	π*	16.8	0.26	0.063
70	C6-C13	π	C12-C14	π*	20.31	0.28	0.069
71	C6-C13	π	C15-C16	π*	19.3	0.28	0.066
72	C7-C10	σ	O1-C9	σ*	3.17	1.09	0.052
73	C7-C10	σ	C4-C5	σ*	3.07	1.16	0.054
74	C7-C10	σ	C4-C7	σ*	4.15	1.27	0.065
75	C7-C10	σ	C9-C10	σ*	2.76	1.28	0.053
76	C7-C10	σ	C10-H19	σ*	1.38	1.17	0.036
77	C7-C10	π	C4-C8	π*	13.12	0.3	0.057
78	C7-C10	π	C7-C10	π*	1.53	0.29	0.019
79	C7-C10	π	C9-C11	π*	24.36	0.29	0.077
80	C8-C11	σ	O1-C9	σ*	4.91	1.07	0.065
81	C8-C11	σ	C4-C5	σ*	2.68	1.14	0.050
82	C8-C11	σ	C4-C8	σ*	2.93	1.27	0.055
83	C8-C11	σ	C8-H18	σ*	1.22	1.19	0.034
84	C8-C11	σ	C9-C11	σ*	2.89	1.25	0.054
85	C8-C11	σ	C11-H20	σ*	1.55	1.17	0.038
86	C8-H18	σ	C4-C7	σ*	4.43	1.06	0.061
87	C8-H18	σ	C4-C8	σ*	1.01	1.09	0.030
88	C8-H18	σ	C8-C11	σ*	0.92	1.11	0.029
89	C8-H18	σ	C9-C11	σ*	3.94	1.07	0.058
90	C9-C10	σ	O1-C17	σ*	2.99	0.98	0.049

91	C9-C10	σ	O2-C7	σ*	3.96	1.06	0.058
92	C9-C10	σ	C7-C10	σ*	2.61	1.27	0.051
93	C9-C10	σ	C9-C11	σ*	3.49	1.26	0.059
94	C9-C10	σ	C10-H19	σ*	1.41	1.16	0.036
95	C9-C10	σ	C11-H20	σ*	2.26	1.18	0.046
96	C9-C11	σ	C8-C11	σ*	2.66	1.3	0.053
97	C9-C11	σ	C8-H18	σ*	2.11	1.2	0.045
98	C9-C11	σ	C9-C10	σ*	3.71	1.27	0.061
99	C9-C11	σ	C10-H19	σ*	2.07	1.16	0.044
100	C9-C11	σ	C11-H20	σ*	1.29	1.18	0.035
101	C9-C11	π	C4-C8	π*	26.48	0.29	0.079
102	C9-C11	π	C7-C10	π*	14.55	0.28	0.057
103	C9-C11	π	C9-C11	π*	1.78	0.28	0.020
104	C10-H19	σ	O1-C9	σ*	0.95	0.9	0.026
105	C10-H19	σ	O2-C7	σ*	0.77	0.88	0.023
106	C10-H19	σ	O2-H26	σ*	0.51	0.96	0.020
107	C10-H19	σ	C4-C7	σ*	4.43	1.08	0.062
108	C10-H19	σ	C7-C10	σ*	0.97	1.1	0.029
109	C10-H19	σ	C9-C10	σ*	0.93	1.09	0.028
110	C10-H19	σ	C9-C11	σ*	4.1	1.08	0.060
111	C11-H20	σ	C4-C8	σ*	3.82	1.1	0.058
112	C11-H20	σ	C8-C11	σ*	1.24	1.12	0.033
113	C11-H20	σ	C9-C10	σ*	3.83	1.09	0.058
114	C11-H20	σ	C9-C11	σ*	0.92	1.08	0.028
115	C12-C14	σ	C5-C6	σ*	3	1.14	0.053
116	C12-C14	σ	C6-C12	σ*	2.84	1.27	0.054
117	C12-C14	σ	C12-H21	σ*	1.28	1.18	0.035
118	C12-C14	σ	C14-C16	σ*	2.5	1.27	0.050
119	C12-C14	σ	C14-H23	σ*	1.16	1.17	0.033
120	C12-C14	σ	C16-H25	σ*	2.3	1.17	0.046
121	C12-C14	π	C6-C13	π *	18.96	0.28	0.066
122	C12-C14	π	C15-C16	π *	21.85	0.28	0.070

123	C12-H21	σ	C6-C12	σ*	1.02	1.09	0.030
124	C12-H21	σ	C6-C13	σ*	4.29	1.09	0.061
125	C12-H21	σ	C12-C14	σ*	0.9	1.1	0.028
126	C12-H21	σ	C14-C16	σ*	3.61	1.09	0.056
127	C13-C15	σ	C5-C6	σ*	3.34	1.14	0.056
128	C13-C15	σ	C6-C13	σ*	3.05	1.27	0.056
129	C13-C15	σ	C13-H22	σ*	1.28	1.17	0.035
130	C13-C15	σ	C15-C16	σ*	2.53	1.27	0.051
131	C13-C15	σ	C15-H24	σ*	1.14	1.17	0.033
132	C13-C15	σ	C16-H25	σ*	2.28	1.17	0.046
133	C13-H22	σ	C6-C12	σ*	4.08	1.1	0.060
134	C13-H22	σ	C6-C13	σ*	1.02	1.1	0.030
135	C13-H22	σ	C13-C15	σ*	0.87	1.11	0.028
136	C13-H22	σ	C15-C16	σ*	3.56	1.1	0.056
137	C14-C16	σ	C12-C14	σ*	2.54	1.28	0.051
138	C14-C16	σ	C12-H21	σ*	2.35	1.18	0.047
139	C14-C16	σ	C14-H23	σ*	1.13	1.17	0.032
140	C14-C16	σ	C15-C16	σ*	2.42	1.27	0.050
141	C14-C16	σ	C15-H24	σ*	2.35	1.17	0.047
142	C14-C16	σ	C16-H25	σ*	1.1	1.17	0.032
143	C14-H23	σ	C6-C12	σ*	3.73	1.09	0.057
144	C14-H23	σ	C12-C14	σ*	0.87	1.11	0.028
145	C14-H23	σ	C14-C16	σ*	0.75	1.1	0.026
146	C14-H23	σ	C15-C16	σ*	3.54	1.1	0.056
147	C15-C16	σ	C13-C15	σ*	2.58	1.28	0.051
148	C15-C16	σ	C13-H22	σ*	2.34	1.17	0.047
149	C15-C16	σ	C14-C16	σ*	2.42	1.27	0.049
150	C15-C16	σ	C14-H23	σ*	2.29	1.17	0.046
151	C15-C16	σ	C15-H24	σ*	1.15	1.17	0.033
152	C15-C16	σ	C16-H25	σ*	1.11	1.17	0.032
153	C15-C16	π	C6-C13	π *	21.54	0.29	0.070
154	C15-C16	π	C12-C14	π *	17.97	0.29	0.065

155	C15-H24	σ	C6-C13	σ*	3.77	1.1	0.058
156	C15-H24	σ	C13-C15	σ*	0.85	1.11	0.027
157	C15-H24	σ	C14-C16	σ*	3.52	1.1	0.056
158	C15-H24	σ	C15-C16	σ*	0.78	1.1	0.026
159	C16-H25	σ	C12-C14	σ*	3.5	1.11	0.056
160	C16-H25	σ	C13-C15	σ*	3.58	1.11	0.056
161	C16-H25	σ	C14-C16	σ*	0.74	1.1	0.025
162	C16-H25	σ	C15-C16	σ*	0.75	1.1	0.026
163	C17-H27	σ	O1-C9	σ*	3.4	0.91	0.050
164	LP(1)O1		C9-C10	σ*	0.67	1.11	0.024
165	LP(1)O1		C9-C11	σ*	7.43	1.11	0.081
166	LP(1)O1		C17-H27	σ*	1.82	1	0.038
167	LP(1)O1		C17-H28	σ*	1.21	0.98	0.031
168	LP(1)O1		C17-H29	σ*	1.24	0.98	0.031
169	LP(2)O1		C9-C11	π*	32.28	0.34	0.100
170	LP(2)O1		C17-H28	σ*	5.33	0.74	0.058
171	LP(2)O1		C17-H29	σ*	5.3	0.74	0.058
172	LP(1)O2		C7-C10	σ*	5.94	1.16	0.074
173	LP(2)O2		C7-C10	π*	30.68	0.35	0.098
174	LP(1)O3		C4-C5	σ*	1.59	1.12	0.038
175	LP(1)O3		C5-C6	σ*	1.83	1.12	0.041
176	LP(2)O3		C4-C5	σ*	19.35	0.69	0.104
177	LP(2)O3		C5-C6	σ*	18.64	0.69	0.103

**Table S2.** The Fock matrix of avobenzone can be analyzed through second order perturbation theory using the Natural Bond Orbital (NBO) method.							
No	Donor (i)	Type	Acceptor(j)	Type	$E^{(2)a}$(KJ mol^{-1})	E(j)-E(i)[b] (a.u)	F(i.j)[C] (a.u)
1	C1-O2	σ	C3-C4	σ*	2.82	1.38	0.056
2	C1-H45	σ	O2-C3	σ*	3.4	0.91	0.050
3	O2-C3	σ	C1-H45	σ*	0.61	1.37	0.026
4	O2-C3	σ	C3-C4	σ*	0.5	1.46	0.024
5	O2-C3	σ	C3-C8	σ*	0.71	1.46	0.029
6	O2-C3	σ	C4-C5	σ*	1.36	1.51	0.040
7	O2-C3	σ	C7-C8	σ*	0.99	1.5	0.034
8	C3-C4	π	C1-O2	π*	3.06	0.98	0.049
9	C3-C4	π	C3-C8	π*	3.31	1.25	0.057
10	C3-C4	π	C4-C5	π*	2.39	1.29	0.050
11	C3-C4	σ	C4-H37	σ*	1.08	1.18	0.032
12	C3-C4	σ	C5-H38	σ*	2.29	1.17	0.046
13	C3-C4	σ	C8-H35	σ*	2.24	1.17	0.046
14	C3-C8	σ	C3-C4	σ*	3.47	1.26	0.059
15	C3-C8	σ	C4-H37	σ*	1.87	1.19	0.042
16	C3-C8	σ	C7-C8	σ*	2.67	1.3	0.053
17	C3-C8	σ	C7-H36	σ*	2.08	1.2	0.045
18	C3-C8	σ	C8-H35	σ*	1.21	1.18	0.034
19	C3-C8	σ	C3-C8	σ*	1.1	0.28	0.016
20	C3-C8	σ	C4-C5	σ*	15.28	0.29	0.061
21	C3-C8	σ	C6-C7	σ*	25.24	0.29	0.077
22	C4-C5	σ	O2-C3	σ*	3.4	1.08	0.054
23	C4-C5	σ	C3-C4	σ*	2.42	1.26	0.049
24	C4-C5	σ	C4-H37	σ*	1.45	1.18	0.037
25	C4-C5	σ	C5-C6	σ*	3.17	1.27	0.057
26	C4-C5	σ	C5-H38	σ*	1.25	1.18	0.034

27	C4-C5	σ	C6-C9	σ*	3.34	1.16	0.056
28	C4-C5	π	C3-C8	π*	22.3	0.28	0.072
29	C4-C5	π	C6-C7	π*	15.55	0.29	0.061
30	C4-H37	σ	O2-C3	σ*	0.95	0.89	0.026
31	C4-H37	σ	C3-C4	σ*	0.7	1.07	0.025
32	C4-H37	σ	C3-C8	σ*	4.2	1.07	0.060
33	C4-H37	σ	C4-C5	σ*	1.17	1.12	0.032
34	C4-H37	σ	C5-C6	σ*	3.9	1.09	0.058
35	C5-C6	σ	C4-C5	σ*	2.75	1.28	0.053
36	C5-C6	σ	C4-H37	σ*	2.31	1.17	0.047
37	C5-C6	σ	C5-H38	σ*	1.22	1.16	0.034
38	C5-C6	σ	C6-C7	σ*	3.62	1.26	0.060
39	C5-C6	σ	C6-C9	σ*	1.88	1.14	0.042
40	C5-C6	σ	C7-H36	σ*	1.98	1.18	0.043
41	C5-C6	σ	C9-O10	σ*	1.62	1.28	0.041
42	C5-H38	σ	C3-C4	σ*	3.9	1.08	0.058
43	C5-H38	σ	C4-C5	σ*	0.93	1.12	0.029
44	C5-H38	σ	C5-C6	σ*	0.93	1.1	0.028
45	C5-H38	σ	C6-C7	σ*	3.86	1.1	0.058
46	C6-C7	σ	C5-C6	σ*	3.59	1.25	0.060
47	C6-C7	σ	C5-H38	σ*	2.37	1.16	0.047
48	C6-C7	σ	C6-C9	σ*	2.05	1.14	0.044
49	C6-C7	σ	C7-C8	σ*	2.45	1.28	0.050
50	C6-C7	σ	C7-H36	σ*	1.15	1.18	0.033
51	C6-C7	σ	C8-H35	σ*	2.32	1.16	0.046
52	C6-C7	σ	C9-C11	σ*	2.07	1.06	0.042
53	C6-C7	π	C3-C8	π*	16.97	0.26	0.060
54	C6-C7	π	C4-C5	π*	22.99	0.28	0.072
55	C6-C7	π	C6-C7	π*	0.99	0.28	0.015
56	C6-C7	π	C9-O10	π*	21.06	0.27	0.071
57	C6-C9	σ	C4-C5	σ*	1.86	1.25	0.043
58	C6-C9	σ	C5-C6	σ*	2.32	1.22	0.048

59	C6-C9	σ	C6-C7	σ*	2.03	1.23	0.045
60	C6-C9	σ	C7-C8	σ*	2.34	1.25	0.048
61	C6-C9	σ	C9-O10	σ*	0.59	1.25	0.024
62	C6-C9	σ	C11-C12	σ*	1.18	1.05	0.032
63	C7-C8	σ	O2-C3	σ*	4.65	1.07	0.063
64	C7-C8	σ	C3-C8	σ*	2.89	1.25	0.054
65	C7-C8	σ	C6-C7	σ*	2.91	1.27	0.054
66	C7-C8	σ	C6-C9	σ*	2.86	1.15	0.052
67	C7-C8	σ	C7-H36	σ*	1.21	1.19	0.034
68	C7-C8	σ	C8-H35	σ*	1.48	1.17	0.037
69	C7-H36	σ	C3-C8	σ*	3.99	1.06	0.058
70	C7-H36	σ	C5-C6	σ*	4.35	1.08	0.061
71	C7-H36	σ	C6-C7	σ*	1.05	1.09	0.030
72	C7-H36	σ	C7-C8	σ*	0.93	1.11	0.029
73	C8-H35	σ	C3-C4	σ*	3.82	1.08	0.057
74	C8-H35	σ	C3-C8	σ*	0.83	1.08	0.027
75	C8-H35	σ	C6-C7	σ*	3.75	1.1	0.057
76	C8-H35	σ	C7-C8	σ*	1.15	1.12	0.032
77	C9-O10	σ	C5-C6	σ*	1.28	1.63	0.041
78	C9-O10	σ	C6-C9	σ*	1.09	1.52	0.037
79	C9-O10	σ	C9-C11	σ*	0.61	1.44	0.027
80	C9-O10	π	C6-C7	π*	3.68	0.4	0.038
81	C9-O10	π	C11-H33	σ*	1.33	0.81	0.029
82	C9-O10	π	C11-H34	σ*	1.47	0.8	0.031
83	C9-C11	σ	C6-C7	σ*	2.25	1.2	0.046
84	C9-C11	σ	C11-C12	σ*	0.51	1.02	0.021
85	C9-C11	σ	C11-H33	σ*	0.66	1.07	0.024
86	C9-C11	σ	C11-H34	σ*	0.61	1.06	0.023
87	C9-C11	σ	C12-O13	σ*	0.7	1.23	0.026
88	C9-C11	σ	C12-O13	π*	2.65	0.66	0.038
89	C11-C12	σ	C6-C9	σ*	2.42	1.07	0.046
90	C11-C12	σ	C9-C11	σ*	0.91	1	0.027

91	C11-C12	σ	C11-H33	σ*	0.66	1.07	0.024
92	C11-C12	σ	C11-H34	σ*	0.69	1.05	0.024
93	C11-C12	σ	C14-C15	σ*	2.21	1.2	0.046
94	C11-H33	σ	C9-O10	σ*	1.93	1.09	0.041
95	C11-H33	σ	C9-O10	π*	4.31	0.52	0.044
96	C11-H33	σ	C11-C12	σ*	0.55	0.9	0.020
97	C11-H33	σ	C12-O13	σ*	0.8	1.11	0.027
98	C11-H33	σ	C12-C14	σ*	3.65	0.96	0.053
99	C11-H34	σ	C9-O10	σ*	1.6	1.1	0.038
100	C11-H34	σ	C9-O10	π*	4.17	0.53	0.043
101	C11-H34	σ	C12-O13	σ*	2.15	1.12	0.044
102	C11-H34	σ	C12-O13	π*	2.64	0.55	0.035
103	C12-O13	σ	C11-C12	σ*	0.67	1.45	0.028
104	C12-O13	σ	C12-C14	σ*	0.97	1.52	0.035
105	C12-O13	σ	C14-C19	σ*	1.27	1.65	0.041
106	C12-O13	π	C9-C11	σ*	1.97	0.74	0.034
107	C12-O13	π	C11-H34	σ*	1.14	0.79	0.027
108	C12-O13	π	C14-C19	π*	5.02	0.4	0.044
109	C12-C14	σ	C11-H33	σ*	0.73	1.1	0.025
110	C12-C14	σ	C12-O13	σ*	0.52	1.25	0.023
111	C12-C14	σ	C14-C15	σ*	1.93	1.23	0.043
112	C12-C14	σ	C14-C19	σ*	2.33	1.24	0.048
113	C12-C14	σ	C15-C16	σ*	2.19	1.26	0.047
114	C12-C14	σ	C18-C19	σ*	1.97	1.24	0.044
115	C14-C15	σ	C11-C12	σ*	2.08	1.07	0.042
116	C14-C15	σ	C12-C14	σ*	1.92	1.13	0.042
117	C14-C15	σ	C14-C19	σ*	3.69	1.26	0.061
118	C14-C15	σ	C15-C16	σ*	2.5	1.28	0.051
119	C14-C15	σ	C15-H39	σ*	1.11	1.17	0.032
120	C14-C15	σ	C16-H42	σ*	2.33	1.16	0.047
121	C14-C15	σ	C19-H40	σ*	2.51	1.15	0.048
122	C14-C19	σ	C12-O13	σ*	1.52	1.29	0.040

123	C14-C19	σ	C12-C14	σ*	1.86	1.15	0.042
124	C14-C19	σ	C14-C15	σ*	3.72	1.27	0.061
125	C14-C19	σ	C15-H39	σ*	1.98	1.19	0.043
126	C14-C19	σ	C18-C19	σ*	2.74	1.28	0.053
127	C14-C19	σ	C18-H41	σ*	2.05	1.17	0.044
128	C14-C19	σ	C19-H40	σ*	1.28	1.17	0.035
129	C14-C19	π	C12-O13	π*	19.29	0.27	0.069
130	C14-C19	π	C15-C16	π*	19.89	0.29	0.069
131	C14-C19	π	C17-C18	π*	17.8	0.29	0.064
132	C15-C16	σ	C12-C14	σ*	2.93	1.15	0.052
133	C15-C16	σ	C14-C15	σ*	2.91	1.27	0.054
134	C15-C16	σ	C15-H39	σ*	1.33	1.19	0.036
135	C15-C16	σ	C16-C17	σ*	3.29	1.26	0.058
136	C15-C16	σ	C16-H42	σ*	1.33	1.17	0.035
137	C15-C16	σ	C17-C20	σ*	3.1	1.1	0.052
138	C15-C16	π	C14-C19	π*	18	0.28	0.064
139	C15-C16	π	C17-C18	π*	21.61	0.29	0.070
140	C15-H39	σ	C14-C15	σ*	1	1.08	0.029
141	C15-H39	σ	C14-C19	σ*	4.19	1.09	0.060
142	C15-H39	σ	C15-C16	σ*	1.02	1.11	0.030
143	C15-H39	σ	C16-C17	σ*	4.08	1.08	0.059
144	C16-C17	σ	C15-C16	σ*	2.93	1.29	0.055
145	C16-C17	σ	C15-H39	σ*	2.09	1.18	0.044
146	C16-C17	σ	C16-H42	σ*	1.15	1.16	0.033
147	C16-C17	σ	C17-C18	σ*	3.27	1.26	0.057
148	C16-C17	σ	C17-C20	σ*	2.02	1.08	0.042
149	C16-C17	σ	C18-H41	σ*	2.44	1.16	0.048
150	C16-C17	σ	C20-C23	σ*	1.22	1.05	0.032
151	C16-H42	σ	C14-C15	σ*	3.83	1.09	0.058
152	C16-H42	σ	C15-C16	σ*	0.93	1.12	0.029
153	C16-H42	σ	C16-C17	σ*	1.03	1.08	0.030
154	C16-H42	σ	C17-C18	σ*	3.82	1.1	0.058

155	C17-C18	σ	C16-C17	σ*	3.37	1.26	0.058
156	C17-C18	σ	C16-H42	σ*	2.26	1.17	0.046
157	C17-C18	σ	C17-C20	σ*	2.32	1.09	0.045
158	C17-C18	σ	C18-C19	σ*	2.99	1.28	0.055
159	C17-C18	σ	C18-H41	σ*	1.39	1.17	0.036
160	C17-C18	σ	C19-H40	σ*	1.97	1.16	0.043
161	C17-C18	π	C14-C19	π*	23.12	0.28	0.072
162	C17-C18	π	C15-C16	π*	16.38	0.29	0.062
163	C17-C18	π	C20-C21	σ*	2.26	0.61	0.036
164	C17-C18	π	C20-C22	σ*	2.24	0.61	0.036
165	C17-C20	σ	C15-C16	σ*	2.05	1.2	0.045
166	C17-C20	σ	C16-C17	σ*	1.93	1.17	0.043
167	C17-C20	σ	C17-C18	σ*	2.23	1.18	0.046
168	C17-C20	σ	C18-C19	σ*	2.03	1.19	0.044
169	C17-C20	σ	C20-C21	σ*	0.73	0.96	0.024
170	C17-C20	σ	C20-C22	σ*	0.73	0.96	0.024
171	C17-C20	σ	C20-C23	σ*	0.64	0.97	0.022
172	C17-C20	σ	C21-H26	σ*	1.49	1.05	0.036
173	C17-C20	σ	C22-H30	σ*	1.48	1.05	0.035
174	C17-C20	σ	C23-H29	σ*	1.17	1.06	0.032
175	C18-C19	σ	C12-C14	σ*	3.47	1.14	0.057
176	C18-C19	σ	C14-C19	σ*	3.17	1.27	0.057
177	C18-C19	σ	C17-C18	σ*	3.32	1.27	0.058
178	C18-C19	σ	C17-C20	σ*	3.51	1.09	0.056
179	C18-C19	σ	C18-H41	σ*	1.28	1.17	0.035
180	C18-C19	σ	C19-H40	σ*	1.25	1.17	0.034
181	C18-H41	σ	C14-C19	σ*	3.84	1.1	0.058
182	C18-H41	σ	C16-C17	σ*	3.81	1.09	0.058
183	C18-H41	σ	C17-C18	σ*	1.27	1.1	0.033
184	C18-H41	σ	C18-C19	σ*	0.83	1.11	0.027
185	C19-H40	σ	C14-C15	σ*	3.93	1.1	0.059
186	C19-H40	σ	C14-C19	σ*	1	1.11	0.030

187	C19-H40	σ	C17-C18	σ*	3.96	1.1	0.059
188	C19-H40	σ	C18-C19	σ*	0.86	1.11	0.028
189	C20-C21	σ	C17-C18	σ*	1.45	1.16	0.037
190	C20-C21	σ	C17-C18	π*	2.04	0.62	0.034
191	C20-C21	σ	C17-C20	σ*	1.01	0.98	0.028
192	C20-C21	σ	C20-C22	σ*	0.65	0.94	0.022
193	C20-C21	σ	C20-C23	σ*	0.75	0.95	0.024
194	C20-C21	σ	C21-H25	σ*	0.52	1.04	0.021
195	C20-C21	σ	C22-H32	σ*	1.69	1.04	0.038
196	C20-C21	σ	C23-H27	σ*	1.89	1.03	0.040
197	C20-C22	σ	C17-C18	σ*	1.48	1.16	0.037
198	C20-C22	σ	C17-C18	π*	1.96	0.62	0.034
199	C20-C22	σ	C17-C20	σ*	1	0.98	0.028
200	C20-C22	σ	C20-C21	σ*	0.65	0.94	0.022
201	C20-C22	σ	C20-C23	σ*	0.74	0.95	0.024
202	C20-C22	σ	C21-H24	σ*	1.67	1.04	0.037
203	C20-C22	σ	C22-H30	σ*	0.51	1.03	0.021
204	C20-C22	σ	C22-H31	σ*	0.52	1.04	0.021
205	C20-C22	σ	C23-H28	σ*	1.88	1.03	0.040
206	C20-C23	σ	C16-C17	σ*	2.55	1.16	0.048
207	C20-C23	σ	C17-C20	σ*	1.07	0.99	0.029
208	C20-C23	σ	C20-C21	σ*	0.78	0.95	0.024
209	C20-C23	σ	C20-C22	σ*	0.78	0.95	0.024
210	C20-C23	σ	C21-H25	σ*	1.69	1.05	0.038
211	C20-C23	σ	C22-H31	σ*	1.7	1.05	0.038
212	C20-C23	σ	C23-H27	σ*	0.53	1.04	0.021
213	C20-C23	σ	C23-H28	σ*	0.53	1.04	0.021
214	C20-C23	σ	C23-H29	σ*	0.59	1.04	0.022
215	C21-H24	σ	C20-C22	σ*	2.85	0.86	0.044
216	C21-H25	σ	C20-C23	σ*	2.62	0.87	0.043
217	C21-H26	σ	C17-C20	σ*	2.86	0.9	0.045
218	C22-H30	σ	C17-C20	σ*	2.87	0.9	0.046

219	C22-H31	σ	C20-C23	σ*	2.61	0.87	0.043
220	C22-H32	σ	C20-C21	σ*	2.84	0.86	0.044
221	C23-H27	σ	C20-C21	σ*	2.74	0.86	0.043
222	C23-H28	σ	C20-C22	σ*	2.75	0.86	0.044
223	C23-H29	σ	C17-C20	σ*	3.38	0.9	0.049
224	C23-H29	σ	C20-C23	σ*	0.53	0.87	0.019
225	LP(1)O2		C1-H43	σ*	1.22	0.98	0.031
226	LP(1)O2		C1-H44	σ*	1.22	0.98	0.031
227	LP(1)O2		C1-H45	σ*	1.83	1	0.038
228	LP(1)O2		C3-C4	σ*	0.69	1.1	0.025
229	LP(1)O2		C3-C8	σ*	7.58	1.1	0.082
230	LP(2)O2		C1-H43	σ*	5.26	0.74	0.058
231	LP(2)O2		C1-H44	σ*	5.27	0.74	0.058
232	LP(2)O2		C3-C8	π*	32.78	0.34	0.099
233	LP(1)O10		C6-C9	σ*	2.08	1.13	0.044
234	LP(1)O10		C9-C11	σ*	1.43	1.05	0.035
235	LP(2)O10		C6-C9	σ*	18.98	0.71	0.105
236	LP(2)O10		C9-C11	σ*	22.21	0.63	0.107
237	LP(2)O10		C12-O13	π*	1.44	0.28	0.018
238	LP(1)O13		C11-C12	σ*	1.45	1.06	0.035
239	LP(1)O13		C12-C14	σ*	2.1	1.13	0.044
240	LP(2)O13		C11-C12	σ*	20.62	0.64	0.104
241	LP(2)O13		C12-C14	σ*	19.16	0.71	0.105

**Table S3.** The Fock matrix of octinoxate can be analyzed through second order perturbation theory using the Natural Bond Orbital (NBO) method.							
No	Donor (i)	Type	Acceptor(j)	Type	$E^{(2)a}$(KJ mol^{-1})	E(j)-E(i)^{b} (a.u)	F(i.j)^{C} (a.u)
1	O1-C8	σ	C4-C5	σ*	1.58	1.19	0.039
2	O1-C8	σ	C12-C13	σ*	2.49	1.26	0.050
3	O1-C12	σ	C4-C8	σ*	1.08	1.29	0.033
4	O1-C12	σ	C13-C14	σ*	1.47	1.55	0.043
5	O2-C12	σ	C12-C13	σ*	1.58	1.52	0.044
6	O2-C12	σ	C13-H39	σ*	0.63	1.54	0.028
7	O2-C12	π	O2-C12	π*	0.92	0.38	0.018
8	O2-C12	π	C13-C14	π*	3.52	0.41	0.035
9	O3-C20	σ	C16-C18	σ*	1.34	1.51	0.040
10	O3-C20	σ	C17-C19	σ*	1.01	1.49	0.035
11	O3-C20	σ	C19-C20	σ*	0.74	1.46	0.030
12	O3-C20	σ	C21-H45	σ*	0.64	1.36	0.026
13	O3-C21	σ	C18-C20	σ*	2.81	1.38	0.056
14	C4-C5	σ	O1-C8	σ*	2.76	0.85	0.043
15	C4-C5	σ	C4-C6	σ*	0.71	0.96	0.023
16	C4-C5	σ	C4-C8	σ*	0.66	0.97	0.023
17	C4-C5	σ	C4-H22	σ*	0.62	1.03	0.023
18	C4-C5	σ	C5-C7	σ*	0.74	0.98	0.024
19	C4-C5	σ	C5-H24	σ*	0.55	1.04	0.021
20	C4-C5	σ	C6-H26	σ*	1.25	1.05	0.032
21	C4-C5	σ	C7-C9	σ*	1.62	0.98	0.036
22	C4-C6	σ	C4-C5	σ*	0.92	0.96	0.027
23	C4-C6	σ	C4-C8	σ*	0.64	0.97	0.022
24	C4-C6	σ	C4-H22	σ*	0.59	1.03	0.022
25	C4-C6	σ	C5-C7	σ*	2.01	0.98	0.040
26	C4-C6	σ	C6-C10	σ*	0.58	0.97	0.021

27	C4-C6	σ	C6-H26	σ*	0.52	1.05	0.021
28	C4-C6	σ	C8-H30	σ*	1.58	1.04	0.036
29	C4-C6	σ	C10-H33	σ*	1.31	1.04	0.033
30	C4-C8	σ	O1-C12	σ*	1.91	0.98	0.039
31	C4-C8	σ	C4-C5	σ*	0.68	0.99	0.023
32	C4-C8	σ	C4-C6	σ*	0.8	0.99	0.025
33	C4-C8	σ	C4-H22	σ*	0.61	1.05	0.023
34	C4-C8	σ	C5-H24	σ*	1.47	1.06	0.035
35	C4-C8	σ	C6-C10	σ*	2.04	1	0.040
36	C4-C8	σ	C8-H30	σ*	0.57	1.07	0.022
37	C4-H22	σ	O1-C8	σ*	0.8	0.75	0.022
38	C4-H22	σ	C5-H23	σ*	2.59	0.92	0.044
39	C4-H22	σ	C6-H25	σ*	2.55	0.93	0.044
40	C4-H22	σ	C8-H29	σ*	2.78	0.93	0.045
41	C5-C7	σ	C4-C5	σ*	0.75	0.97	0.024
42	C5-C7	σ	C4-C6	σ*	1.74	0.97	0.037
43	C5-C7	σ	C5-H23	σ*	0.56	1.03	0.022
44	C5-C7	σ	C5-H24	σ*	0.65	1.04	0.023
45	C5-C7	σ	C7-C9	σ*	0.66	0.98	0.023
46	C5-C7	σ	C7-H27	σ*	0.53	1.03	0.021
47	C5-C7	σ	C7-H28	σ*	0.58	1.04	0.022
48	C5-C7	σ	C9-C11	σ*	1.87	0.98	0.038
49	C5-H23	σ	C4-H22	σ*	2.52	0.94	0.043
50	C5-H23	σ	C7-H27	σ*	2.62	0.93	0.044
51	C5-H24	σ	C4-C8	σ*	2.89	0.88	0.045
52	C5-H24	σ	C7-H28	σ*	2.88	0.94	0.047
53	C6-C10	σ	C4-C6	σ*	0.76	0.97	0.024
54	C6-C10	σ	C4-C8	σ*	1.85	0.98	0.038
55	C6-C10	σ	C6-H25	σ*	0.53	1.04	0.021
56	C6-C10	σ	C6-H26	σ*	0.57	1.06	0.022
57	C6-C10	σ	C10-H33	σ*	0.54	1.05	0.021
58	C6-H25	σ	C4-H22	σ*	2.6	0.93	0.044

59	C6-H25	σ	C10-H34	σ*	2.73	0.94	0.045
60	C6-H26	σ	C4-C5	σ*	3.21	0.86	0.047
61	C6-H26	σ	C10-H35	σ*	2.98	0.94	0.047
62	C7-C9	σ	C4-C5	σ*	2.14	0.97	0.041
63	C7-C9	σ	C5-C7	σ*	0.75	0.98	0.024
64	C7-C9	σ	C7-H27	σ*	0.55	1.03	0.021
65	C7-C9	σ	C7-H28	σ*	0.58	1.04	0.022
66	C7-C9	σ	C9-C11	σ*	0.54	0.98	0.020
67	C7-C9	σ	C9-H31	σ*	0.5	1.04	0.020
68	C7-C9	σ	C11-H36	σ*	1.47	1.04	0.035
69	C7-H27	σ	C5-H23	σ*	2.8	0.93	0.046
70	C7-H27	σ	C9-H31	σ*	2.69	0.94	0.045
71	C7-H28	σ	C5-H24	σ*	2.46	0.94	0.043
72	C7-H28	σ	C9-H32	σ*	2.69	0.94	0.045
73	C8-H29	σ	C4-H22	σ*	2.55	0.96	0.044
74	C8-H30	σ	C4-C6	σ*	2.91	0.9	0.046
75	C8-H30	σ	C4-C8	σ*	0.57	0.9	0.020
76	C9-C11	σ	C5-C7	σ*	2.07	0.98	0.040
77	C9-C11	σ	C7-C9	σ*	0.68	0.98	0.023
78	C9-C11	σ	C9-H31	σ*	0.52	1.04	0.021
79	C9-C11	σ	C9-H32	σ*	0.52	1.04	0.021
80	C9-C11	σ	C11-H36	σ*	0.52	1.05	0.021
81	C9-H31	σ	C7-H27	σ*	2.77	0.93	0.045
82	C9-H31	σ	C11-H38	σ*	2.81	0.94	0.046
83	C9-H32	σ	C7-H28	σ*	2.71	0.94	0.045
84	C9-H32	σ	C11-H37	σ*	2.8	0.94	0.046
85	C10-H33	σ	C4-C6	σ*	3.35	0.87	0.048
86	C10-H34	σ	C6-H25	σ*	2.68	0.94	0.045
87	C10-H35	σ	C6-H26	σ*	2.3	0.96	0.042
88	C11-H36	σ	C7-C9	σ*	3.1	0.89	0.047
89	C11-H37	σ	C9-H32	σ*	2.62	0.94	0.044
90	C11-H38	σ	C9-H31	σ*	2.61	0.95	0.044

91	C12-C13	σ	O1-C8	σ*	3.52	0.94	0.051
92	C12-C13	σ	O2-C12	σ*	1.1	1.27	0.033
93	C12-C13	σ	C13-C14	σ*	2.11	1.32	0.047
94	C12-C13	σ	C13-H39	σ*	0.52	1.14	0.022
95	C12-C13	σ	C14-C15	σ*	4.44	1.17	0.064
96	C13-C14	σ	O1-C12	σ*	1.8	1.11	0.041
97	C13-C14	σ	C12-C13	σ*	1.69	1.19	0.040
98	C13-C14	σ	C13-H39	σ*	1.91	1.21	0.043
99	C13-C14	σ	C14-C15	σ*	2.85	1.24	0.053
100	C13-C14	σ	C14-H40	σ*	1.56	1.21	0.039
101	C13-C14	σ	C15-C17	σ*	1.83	1.3	0.044
102	C13-C14	π	O2-C12	π*	22	0.29	0.074
103	C13-C14	π	C15-C17	π*	11.18	0.3	0.055
104	C13-H39	σ	O1-C12	σ*	0.57	0.9	0.021
105	C13-H39	σ	O2-C12	σ*	4.26	1.13	0.062
106	C13-H39	σ	C13-C14	σ*	1.89	1.18	0.042
107	C13-H39	σ	C14-H40	σ*	3.93	1	0.056
108	C14-C15	σ	C12-C13	σ*	2.25	1.12	0.045
109	C14-C15	σ	C13-C14	σ*	2.97	1.32	0.056
110	C14-C15	σ	C14-H40	σ*	0.54	1.14	0.022
111	C14-C15	σ	C15-C16	σ*	2.68	1.22	0.051
112	C14-C15	σ	C15-C17	σ*	2.44	1.23	0.049
113	C14-C15	σ	C16-C18	σ*	1.76	1.26	0.042
114	C14-C15	σ	C17-C19	σ*	2.13	1.25	0.046
115	C14-H40	σ	C13-C14	σ*	1.41	1.17	0.036
116	C14-H40	σ	C13-H39	σ*	5.28	0.99	0.065
117	C14-H40	σ	C15-C16	σ*	4.61	1.07	0.063
118	C15-C16	σ	C14-C15	σ*	2.67	1.19	0.050
119	C15-C16	σ	C14-H40	σ*	1.15	1.16	0.033
120	C15-C16	σ	C15-C17	σ*	3.3	1.25	0.057
121	C15-C16	σ	C16-C18	σ*	2.68	1.28	0.053
122	C15-C16	σ	C16-H41	σ*	1.07	1.16	0.032

123	C15-C16	σ	C17-H42	σ*	2.18	1.16	0.045
124	C15-C16	σ	C18-H43	σ*	2.34	1.16	0.047
125	C15-C17	σ	C13-C14	σ*	1.89	1.35	0.045
126	C15-C17	σ	C14-C15	σ*	2.4	1.2	0.048
127	C15-C17	σ	C15-C16	σ*	3.27	1.25	0.057
128	C15-C17	σ	C16-H41	σ*	2.2	1.16	0.045
129	C15-C17	σ	C17-C19	σ*	2.56	1.27	0.051
130	C15-C17	σ	C17-H42	σ*	1.1	1.16	0.032
131	C15-C17	σ	C19-H44	σ*	2.18	1.16	0.045
132	C15-C17	π	C13-C14	π*	17.9	0.29	0.069
133	C15-C17	π	C15-C17	π*	0.69	0.28	0.012
134	C15-C17	π	C16-C18	π*	20.37	0.28	0.069
135	C15-C17	π	C19-C20	π*	17.77	0.27	0.062
136	C16-C18	σ	O3-C20	σ*	3.38	1.07	0.054
137	C16-C18	σ	C14-C15	σ*	3.23	1.21	0.056
138	C16-C18	σ	C15-C16	σ*	2.96	1.26	0.055
139	C16-C18	σ	C16-H41	σ*	1.25	1.18	0.034
140	C16-C18	σ	C18-C20	σ*	2.45	1.25	0.049
141	C16-C18	σ	C18-H43	σ*	1.46	1.18	0.037
142	C16-C18	π	C15-C17	π*	16.05	0.29	0.062
143	C16-C18	π	C19-C20	π*	21.33	0.28	0.071
144	C16-H41	σ	C15-C16	σ*	0.89	1.08	0.028
145	C16-H41	σ	C15-C17	σ*	3.85	1.09	0.058
146	C16-H41	σ	C16-C18	σ*	0.94	1.12	0.029
147	C16-H41	σ	C18-C20	σ*	4.01	1.07	0.059
148	C17-C19	σ	O3-C20	σ*	4.78	1.07	0.064
149	C17-C19	σ	C14-C15	σ*	3.01	1.21	0.054
150	C17-C19	σ	C15-C17	σ*	2.88	1.27	0.054
151	C17-C19	σ	C17-H42	σ*	1.1	1.17	0.032
152	C17-C19	σ	C19-C20	σ*	2.96	1.25	0.054
153	C17-C19	σ	C19-H44	σ*	1.46	1.17	0.037
154	C17-H42	σ	C15-C16	σ*	4.09	1.08	0.060

155	C17-H42	σ	C15-C17	σ*	0.89	1.09	0.028
156	C17-H42	σ	C17-C19	σ*	0.86	1.11	0.027
157	C17-H42	σ	C19-C20	σ*	3.88	1.08	0.058
158	C18-C20	σ	O3-C21	σ*	3.02	0.98	0.049
159	C18-C20	σ	C16-C18	σ*	2.41	1.3	0.050
160	C18-C20	σ	C16-H41	σ*	2.3	1.17	0.047
161	C18-C20	σ	C18-H43	σ*	1.05	1.18	0.032
162	C18-C20	σ	C19-C20	σ*	3.37	1.25	0.058
163	C18-C20	σ	C19-H44	σ*	2.34	1.17	0.047
164	C18-H43	σ	O3-C20	σ*	0.95	0.89	0.026
165	C18-H43	σ	C15-C16	σ*	3.98	1.08	0.059
166	C18-H43	σ	C16-C18	σ*	1.2	1.12	0.033
167	C18-H43	σ	C18-C20	σ*	0.66	1.07	0.024
168	C18-H43	σ	C19-C20	σ*	4.16	1.08	0.060
169	C19-C20	σ	C17-C19	σ*	2.71	1.3	0.053
170	C19-C20	σ	C17-H42	σ*	2.09	1.18	0.044
171	C19-C20	σ	C18-C20	σ*	3.51	1.26	0.060
172	C19-C20	σ	C18-H43	σ*	1.84	1.19	0.042
173	C19-C20	σ	C19-H44	σ*	1.27	1.18	0.035
174	C19-C20	π	C15-C17	π*	22.83	0.29	0.074
175	C19-C20	π	C16-C18	π*	15.3	0.3	0.061
176	C19-C20	π	C19-C20	π*	0.82	0.28	0.014
177	C19-H44	σ	C15-C17	σ*	3.78	1.1	0.058
178	C19-H44	σ	C17-C19	σ*	1.1	1.12	0.031
179	C19-H44	σ	C18-C20	σ*	3.82	1.08	0.057
180	C19-H44	σ	C19-C20	σ*	0.89	1.09	0.028
181	C21-H45	σ	O3-C20	σ*	3.4	0.91	0.050
182	LP (1)O1		O2-C12	σ*	7.78	1.16	0.085
183	LP (1)O1		C4-C5	σ*	0.53	0.94	0.020
184	LP (1)O1		C4-C8	σ*	0.98	0.95	0.027
185	LP (1)O1		C8-H29	σ*	0.98	1.01	0.028
186	LP (1)O1		C8-H30	σ*	0.93	1.02	0.028

187	LP (2)O1		O2-C12	π*	46.84	0.33	0.113
188	LP (2)O1		C8-H29	σ*	4.48	0.76	0.055
189	LP (2)O1		C8-H30	σ*	4.14	0.78	0.053
190	LP (1)O2		O1-C12	σ*	1.31	1.05	0.034
191	LP (1)O2		C12-C13	σ*	2.81	1.13	0.051
192	LP (2)O2		O1-C12	σ*	34	0.62	0.132
193	LP (2)O2		C4-C8	σ*	0.83	0.64	0.021
194	LP (2)O2		C12-C13	σ*	17.69	0.7	0.102
195	LP (1)O3		C18-C20	σ*	0.68	1.1	0.025
196	LP (1)O3		C19-C20	σ*	7.54	1.11	0.082
197	LP (1)O3		C21-H45	σ*	1.83	1	0.039
198	LP (1)O3		C21-H46	σ*	1.23	0.98	0.031
199	LP (1)O3		C21-H47	σ*	1.24	0.98	0.031
200	LP (2)O3		C19-C20	π*	32.12	0.34	0.099
201	LP (2)O3		C21-H46	σ*	5.34	0.74	0.058
202	LP (2)O3		C21-H47	σ*	5.35	0.74	0.058

**Table S4.** The Fock matrix of padimate can be analyzed through second order perturbation theory using the Natural Bond Orbital (NBO) method.							
No	Donor(i)	Type	Acceptor(j)	Type	$E^{(2)a}$(KJ mol^{-1})	E(j)-(i)^{b}(a.u)	F(i.j)^{C}(a.u)
1	O1-C8	σ	C4-C5	σ*	1.58	1.19	0.039
2	O1-C8	σ	C12-C13	σ*	2.66	1.26	0.052
3	O1-C12	σ	C4-C8	σ*	1.05	1.29	0.033
4	O1-C12	σ	C13-C16	σ*	1.72	1.48	0.045
5	O2-C12	σ	C12-C13	σ*	1.7	1.52	0.046
6	O2-C12	σ	C13-C15	σ*	1.19	1.64	0.040
7	O2-C12	π	O2-C12	π*	0.81	0.38	0.017
8	O2-C12	π	C13-C15	π*	4.11	0.4	0.040
9	N3-C14	σ	N3-C19	σ*	1.05	1.15	0.031
10	N3-C14	σ	N3-C20	σ*	1.05	1.15	0.031
11	N3-C14	σ	C14-C17	σ*	1.56	1.35	0.041
12	N3-C14	σ	C14-C18	σ*	1.55	1.35	0.041
13	N3-C14	σ	C15-C17	σ*	1.09	1.41	0.035
14	N3-C14	σ	C16-C18	σ*	1.07	1.41	0.035
15	N3-C14	σ	C19-H43	σ*	0.59	1.25	0.024
16	N3-C14	σ	C20-H45	σ*	0.59	1.25	0.024
17	N3-C19	σ	N3-C14	σ*	1.61	1.22	0.040
18	N3-C19	σ	N3-C20	σ*	0.56	1.08	0.022
19	N3-C19	σ	C14-C17	σ*	3.05	1.28	0.056
20	N3-C19	σ	C20-H46	σ*	0.56	1.17	0.023
21	N3-C20	σ	N3-C14	σ*	1.6	1.22	0.040
22	N3-C20	σ	N3-C19	σ*	0.56	1.08	0.022
23	N3-C20	σ	C14-C18	σ*	3.04	1.28	0.056
24	N3-C20	σ	C19-H42	σ*	0.57	1.17	0.023
25	C4-C5	σ	O1-C8	σ*	2.72	0.86	0.043
26	C4-C5	σ	C4-C6	σ*	0.7	0.96	0.023

27	C4-C5	σ	C4-C8	σ*	0.67	0.97	0.023
28	C4-C5	σ	C4-H21	σ*	0.62	1.03	0.023
29	C4-C5	σ	C5-C7	σ*	0.75	0.98	0.024
30	C4-C5	σ	C5-H23	σ*	0.55	1.04	0.021
31	C4-C5	σ	C6-H25	σ*	1.25	1.05	0.033
32	C4-C5	σ	C7-C9	σ*	1.61	0.98	0.036
33	C4-C6	σ	C4-C5	σ*	0.92	0.96	0.027
34	C4-C6	σ	C4-C8	σ*	0.64	0.97	0.022
35	C4-C6	σ	C4-H21	σ*	0.59	1.03	0.022
36	C4-C6	σ	C5-C7	σ*	2.03	0.98	0.040
37	C4-C6	σ	C6-C10	σ*	0.58	0.97	0.021
38	C4-C6	σ	C6-H25	σ*	0.52	1.05	0.021
39	C4-C6	σ	C8-H29	σ*	1.58	1.04	0.036
40	C4-C6	σ	C10-H32	σ*	1.31	1.04	0.033
41	C4-C8	σ	O1-C12	σ*	1.92	0.98	0.040
42	C4-C8	σ	C4-C5	σ*	0.69	0.99	0.023
43	C4-C8	σ	C4-C6	σ*	0.8	0.98	0.025
44	C4-C8	σ	C4-H21	σ*	0.61	1.05	0.023
45	C4-C8	σ	C5-H23	σ*	1.46	1.06	0.035
46	C4-C8	σ	C6-C10	σ*	2.04	1	0.040
47	C4-C8	σ	C8-H29	σ*	0.56	1.07	0.022
48	C4-H21	σ	O1-C8	σ*	0.81	0.75	0.022
49	C4-H21	σ	C5-H22	σ*	2.6	0.92	0.044
50	C4-H21	σ	C6-H24	σ*	2.55	0.93	0.044
51	C4-H21	σ	C8-H28	σ*	2.74	0.93	0.045
52	C5-C7	σ	C4-C5	σ*	0.76	0.97	0.024
53	C5-C7	σ	C4-C6	σ*	1.73	0.97	0.037
54	C5-C7	σ	C5-H22	σ*	0.56	1.03	0.022
55	C5-C7	σ	C5-H23	σ*	0.65	1.04	0.023
56	C5-C7	σ	C7-C9	σ*	0.66	0.98	0.023
57	C5-C7	σ	C7-H26	σ*	0.53	1.03	0.021
58	C5-C7	σ	C7-H27	σ*	0.58	1.04	0.022

59	C5-C7	σ	C9-C11	σ*	1.88	0.98	0.038
60	C5-H22	σ	C4-H21	σ*	2.53	0.94	0.043
61	C5-H22	σ	C7-H26	σ*	2.63	0.93	0.044
62	C5-H23	σ	C4-C8	σ*	2.89	0.88	0.045
63	C5-H23	σ	C7-H27	σ*	2.88	0.94	0.047
64	C6-C10	σ	C4-C6	σ*	0.76	0.97	0.024
65	C6-C10	σ	C4-C8	σ*	1.84	0.98	0.038
66	C6-C10	σ	C6-H24	σ*	0.53	1.04	0.021
67	C6-C10	σ	C6-H25	σ*	0.57	1.06	0.022
68	C6-C10	σ	C10-H32	σ*	0.54	1.05	0.021
69	C6-C10	σ	C10-H34	σ*	0.5	1.05	0.020
70	C6-H24	σ	C4-H21	σ*	2.6	0.93	0.044
71	C6-H24	σ	C10-H33	σ*	2.74	0.94	0.045
72	C6-H25	σ	C4-C5	σ*	3.18	0.87	0.047
73	C6-H25	σ	C10-H34	σ*	2.98	0.94	0.047
74	C7-C9	σ	C4-C5	σ*	2.15	0.97	0.041
75	C7-C9	σ	C5-C7	σ*	0.75	0.98	0.024
76	C7-C9	σ	C7-H26	σ*	0.54	1.03	0.021
77	C7-C9	σ	C7-H27	σ*	0.58	1.04	0.022
78	C7-C9	σ	C9-C11	σ*	0.54	0.98	0.020
79	C7-C9	σ	C9-H30	σ*	0.5	1.04	0.020
80	C7-C9	σ	C11-H35	σ*	1.47	1.04	0.035
81	C7-H26	σ	C5-H22	σ*	2.79	0.93	0.045
82	C7-H26	σ	C9-H30	σ*	2.7	0.94	0.045
83	C7-H27	σ	C5-H23	σ*	2.46	0.94	0.043
84	C7-H27	σ	C9-H31	σ*	2.69	0.94	0.045
85	C8-H28	σ	C4-H21	σ*	2.55	0.96	0.044
86	C8-H29	σ	C4-C6	σ*	2.91	0.89	0.046
87	C8-H29	σ	C4-C8	σ*	0.55	0.9	0.020
88	C9-C11	σ	C5-C7	σ*	2.07	0.98	0.040
89	C9-C11	σ	C7-C9	σ*	0.68	0.98	0.023
90	C9-C11	σ	C9-H30	σ*	0.52	1.04	0.021

91	C9-C11	σ	C9-H31	σ*	0.51	1.04	0.021
92	C9-C11	σ	C11-H35	σ*	0.52	1.05	0.021
93	C9-H30	σ	C7-H26	σ*	2.77	0.93	0.045
94	C9-H30	σ	C11-H37	σ*	2.81	0.94	0.046
95	C9-H31	σ	C7-H27	σ*	2.72	0.94	0.045
96	C9-H31	σ	C11-H36	σ*	2.79	0.94	0.046
97	C10-H32	σ	C4-C6	σ*	3.34	0.87	0.048
98	C10-H33	σ	C6-H24	σ*	2.68	0.94	0.045
99	C10-H34	σ	C6-H25	σ*	2.3	0.96	0.042
100	C11-H35	σ	C7-C9	σ*	3.09	0.89	0.047
101	C11-H36	σ	C9-H31	σ*	2.62	0.94	0.044
102	C11-H37	σ	C9-H30	σ*	2.61	0.95	0.044
103	C12-C13	σ	O1-C8	σ*	3.36	0.94	0.050
104	C12-C13	σ	O2-C12	σ*	1.05	1.27	0.033
105	C12-C13	σ	C13-C15	σ*	2.42	1.24	0.049
106	C12-C13	σ	C13-C16	σ*	1.8	1.24	0.042
107	C12-C13	σ	C15-C17	σ*	1.91	1.27	0.044
108	C12-C13	σ	C16-C18	σ*	2.38	1.27	0.049
109	C13-C15	σ	O2-C12	σ*	2.09	1.29	0.046
110	C13-C15	σ	C12-C13	σ*	1.79	1.14	0.041
111	C13-C15	σ	C13-C16	σ*	3.86	1.26	0.062
112	C13-C15	σ	C15-C17	σ*	2.64	1.29	0.052
113	C13-C15	σ	C15-H38	σ*	1.22	1.17	0.034
114	C13-C15	σ	C16-H39	σ*	2.13	1.18	0.045
115	C13-C15	σ	C17-H40	σ*	2.23	1.16	0.046
116	C13-C15	π	O2-C12	π*	25.43	0.26	0.074
117	C13-C15	π	C13-C15	π*	1.26	0.28	0.017
118	C13-C15	π	C14-C17	π*	16.31	0.26	0.060
119	C13-C15	π	C16-C18	π*	23.96	0.28	0.074
120	C13-C16	σ	O1-C12	σ*	2.15	1.06	0.043
121	C13-C16	σ	C12-C13	σ*	1.89	1.14	0.042
122	C13-C16	σ	C13-C15	σ*	3.82	1.26	0.062

123	C13-C16	σ	C15-H38	σ*	2.35	1.17	0.047
124	C13-C16	σ	C16-C18	σ*	2.55	1.29	0.051
125	C13-C16	σ	C16-H39	σ*	1.17	1.17	0.033
126	C13-C16	σ	C18-H41	σ*	2.31	1.16	0.046
127	C14-C17	σ	N3-C14	σ*	1.67	1.17	0.039
128	C14-C17	σ	N3-C19	σ*	3.42	1.03	0.053
129	C14-C17	σ	C14-C18	σ*	3.3	1.23	0.057
130	C14-C17	σ	C15-C17	σ*	2.74	1.29	0.053
131	C14-C17	σ	C15-H38	σ*	1.97	1.17	0.043
132	C14-C17	σ	C17-H40	σ*	1.13	1.16	0.033
133	C14-C17	σ	C18-H41	σ*	2.12	1.16	0.045
134	C14-C17	π	C13-C15	π*	26.94	0.28	0.079
135	C14-C17	π	C14-C17	π*	0.99	0.27	0.015
136	C14-C17	π	C16-C18	π*	13.92	0.29	0.058
137	C14-C18	σ	N3-C14	σ*	1.67	1.17	0.039
138	C14-C18	σ	N3-C20	σ*	3.43	1.03	0.053
139	C14-C18	σ	C14-C17	σ*	3.29	1.23	0.057
140	C14-C18	σ	C16-C18	σ*	2.76	1.29	0.053
141	C14-C18	σ	C16-H39	σ*	2.02	1.18	0.044
142	C14-C18	σ	C17-H40	σ*	2.14	1.16	0.045
143	C14-C18	σ	C18-H41	σ*	1.12	1.16	0.032
144	C15-C17	σ	N3-C14	σ*	3.79	1.18	0.060
145	C15-C17	σ	C12-C13	σ*	3.34	1.16	0.056
146	C15-C17	σ	C13-C15	σ*	3.15	1.28	0.057
147	C15-C17	σ	C14-C17	σ*	3.08	1.24	0.055
148	C15-C17	σ	C15-H38	σ*	1.22	1.18	0.034
149	C15-C17	σ	C17-H40	σ*	1.49	1.17	0.037
150	C15-H38	σ	C13-C15	σ*	0.92	1.1	0.028
151	C15-H38	σ	C13-C16	σ*	4.01	1.1	0.059
152	C15-H38	σ	C14-C17	σ*	4.38	1.06	0.061
153	C15-H38	σ	C15-C17	σ*	1.04	1.12	0.030
154	C16-C18	σ	N3-C14	σ*	3.81	1.18	0.060

155	C16-C18	σ	C12-C13	σ*	2.82	1.16	0.051
156	C16-C18	σ	C13-C16	σ*	3.06	1.28	0.056
157	C16-C18	σ	C14-C18	σ*	3.08	1.24	0.055
158	C16-C18	σ	C16-H39	σ*	1.25	1.19	0.035
159	C16-C18	σ	C18-H41	σ*	1.51	1.17	0.038
160	C16-C18	π	C13-C15	π*	14.36	0.29	0.059
161	C16-C18	π	C14-C17	π*	22.04	0.27	0.072
162	C16-H39	σ	C13-C15	σ*	4.15	1.09	0.060
163	C16-H39	σ	C13-C16	σ*	0.92	1.09	0.028
164	C16-H39	σ	C14-C18	σ*	4.36	1.06	0.061
165	C16-H39	σ	C16-C18	σ*	1.06	1.12	0.031
166	C17-H40	σ	C13-C15	σ*	3.94	1.1	0.059
167	C17-H40	σ	C14-C17	σ*	0.81	1.07	0.026
168	C17-H40	σ	C14-C18	σ*	3.75	1.07	0.057
169	C17-H40	σ	C15-C17	σ*	1.14	1.13	0.032
170	C18-H41	σ	C13-C16	σ*	3.9	1.1	0.058
171	C18-H41	σ	C14-C17	σ*	3.76	1.07	0.057
172	C18-H41	σ	C14-C18	σ*	0.8	1.07	0.026
173	C18-H41	σ	C16-C18	σ*	1.17	1.13	0.033
174	C19-H42	σ	N3-C20	σ*	2.31	0.87	0.040
175	C19-H43	σ	N3-C14	σ*	3.75	1.01	0.055
176	C19-H44	σ	N3-C20	σ*	1.24	0.87	0.029
177	C20-H45	σ	N3-C14	σ*	3.75	1.01	0.055
178	C20-H46	σ	N3-C19	σ*	2.26	0.87	0.040
179	C20-H47	σ	N3-C19	σ*	1.27	0.87	0.030
180	LP(1)O1		O2-C12	σ*	7.48	1.16	0.084
181	LP(1)O1		C4-C5	σ*	0.54	0.94	0.020
182	LP(1)O1		C4-C8	σ*	0.99	0.95	0.027
183	LP(1)O1		C8-H28	σ*	1.19	1.01	0.031
184	LP(1)O1		C8-H29	σ*	0.76	1.02	0.025
185	LP(2)O1		O2-C12	π*	46.13	0.33	0.113
186	LP(2)O1		C8-H28	σ*	4.19	0.76	0.053

187	LP(2)O1		C8-H29	σ*	4.47	0.78	0.055
188	LP(1)O2		O1-C12	σ*	1.23	1.05	0.033
189	LP(1)O2		C12-C13	σ*	2.88	1.13	0.051
190	LP(2)O2		O1-C12	σ*	33.96	0.62	0.132
191	LP(2)O2		C4-C8	σ*	0.87	0.64	0.022
192	LP(2)O2		C12-C13	σ*	17.55	0.7	0.102
193	LP(1)N3		C14-C17	π*	45.83	0.27	0.103
194	LP(1)N3		C19-H42	σ*	4.46	0.67	0.053
195	LP(1)N3		C19-H44	σ*	6.47	0.67	0.063
196	LP(1)N3		C20-H46	σ*	4.57	0.67	0.053
197	LP(1)N3		C20-H47	σ*	6.4	0.67	0.063

**Table S5.** Oxybenzone's significant NLMO occupancy, percentage derived from its parent NBO, and atomic hybrid contributions					
No	Bond	Occupancy	Percentage from parent NBO (%)	Hybrid contributions	
				Atom	Percentage (%)
1	BD (1) O 1 -C 9	2.00000	99.5894	O1<br>C9	67.096<br>32.500
2	BD (1) O 1- C17	2.00000	99.6443	O1<br>C17	67.966<br>31.682
3	BD (1) O 2 -C 7	2.00000	99.7197	O2<br>C7	66.742<br>32.983
4	BD (1) O 2- H26	2.00000	99.4239	O2<br>H26	74.717<br>24.707
5	BD (1) O 3 -C 5	2.00000	99.7109	O3<br>C5	65.410<br>34.316
6	BD (2) O 3 -C 5	2.00000	98.4586	O3<br>C5	65.667<br>32.793
7	BD (1) C 4 -C 5	2.00000	98.8156	C4<br>C5	51.983<br>46.935
8	BD (1) C 4 -C 7	2.00000	98.4490	C4<br>C7	49.758<br>48.892
9	BD (1) C 4 -C 8	2.00000	98.4087	C4<br>C8	50.453<br>48.171
10	BD (2) C 4 -C 8	2.00000	81.9948	C4<br>C8	48.811<br>33.243
11	BD (1) C 5 -C 6	2.00000	98.9232	C5	47.546

				C6	47.546
12	BD (1) C 6- C12	2.00000	98.6786	C6<br>C12	50.586<br>48.293
13	BD (1) C 6 -C13	2.00000	98.7612	C6<br>C13	50.386<br>48.582
14	BD (2) C 6- C13	2.00000	81.2882	C6<br>C13	43.259<br>38.036
15	BD (1) C 7- C10	2.00000	98.8750	C7<br>C10	49.597<br>49.446
16	BD (2) C 7 -C10	2.00000	83.2024	C7<br>C10	33.800<br>49.472
17	BD (1) C 8 -C11	2.00000	98.7475	C8<br>C11	49.119<br>49.808
18	BD (1) C 8- H18	2.00000	98.9383	C8<br>H18	62.515<br>36.475
19	BD (1) C 9 -C10	2.00000	98.5667	C9<br>C10	49.393<br>49.352
20	BD (1) C 9- C11	2.00000	99.0192	C9<br>C11	49.949<br>49.234
21	BD (2) C 9 -C11	2.00000	81.2789	C9<br>C11	32.534<br>48.859
22	BD (1) C10- H19	2.00000	98.7631	C10<br>H19	61.502<br>37.307
23	BD (1) C11-H20	2.00000	98.8946	C11<br>H20	61.702<br>37.253
24	BD (1) C12- C14	2.00000	99.0119	C12<br>C14	49.647<br>49.519
25	BD (2) C12- C14	2.00000	81.0960	O3<br>C5<br>C6<br>C12<br>C13<br>C14	0.041<br>0.089<br>4.113<br>39.089<br>5.012<br>42.008

				C15<br>C16	4.565<br>5.060
26	BD (1) C12- H21	2.00000	99.0386	C12<br>H21	62.534<br>42.008
27	BD (1) C13- C15	2.00000	98.9878	C13<br>C15	49.796<br>49.352
28	BD (1) C13- H22	2.00000	99.0471	C15<br>H22	61.804<br>37.298
29	BD (1) C14- C16	2.00000	99.0569	C14<br>C16	49.547<br>49.656
30	BD (1) C14- H23	2.00000	99.1371	C14<br>H23	61.706<br>37.477
31	BD (1) C15- C16	2.00000	99.0516	C15<br>C16	49.636
32	BD (2) C15- C16	2.00000	82.0037	C15<br>C16	39.947<br>42.106
33	BD (1) C15 -H24	2.00000	99.1383	C15<br>H24	61.624<br>37.560
34	BD (1) C16- H25	2.00000	99.1608	C16<br>H25	61.621<br>37.584
35	BD (1) C17- H27	2.00000	99.5422	C17<br>H27	61.495<br>38.052
36	BD (1) C17-H28	2.00000	99.7861	C17<br>H28	60.969<br>38.828
37	BD (1) C17 -H29	2.00000	99.7845	C17<br>H29	60.917<br>38.878
38	LP (2) O 1	2.00000	91.2035	O1	91.204
39	LP (1) O 2	2.00000	98.9155	O2	98.916
40	LP (2) O 2	2.00000	93.2204	O2	93.220
41	LP (1) O 3	2.00000	98.9268	O3	98.927
42	LP (2) O 3	2.00000	94.3765	O3	94.378

**Table S6.** Avobenzone's significant NLMO occupancy, percentage derived from its parent NBO, and atomic hybrid contributions					
No	Bağ	Occupancy	Percentage from parent NBO (%)	Hybrid contributions	
				Atom	Percentage (%)
1	BD (1) O1-C8	2.00000	99.4530	O1<br>C8	68.691<br>30.766
2	BD (1) O1-C12	2.00000	99.5988	O1<br>C12	68.969<br>30.638
3	BD (1) C1- H44	2.00000	979.738	C1<br>H44	60.933<br>38.852
4	BD (1) C1- H45	2.00000	99.545	C1<br>H45	61.593<br>37.957
5	BD (1) O2 -C3	2.00000	99.6069	O2<br>C3	67.150<br>32.464
6	BD (1) C3 -C4	2.00000	98.7429	C3<br>C4	50.016<br>48.882
7	BD (1) C3 -C8	2.00000	99.0522	C3<br>C8	49.790<br>49.419
8	BD (2) C3 -C8	2.00000	80.3758	C3<br>C8	32.906<br>47.611
9	BD (1) C4 -C5	2.00000	98.8501	C4<br>C5	49.424<br>49.601
10	BD (2) C4 -C5	2.00000	83.9538	C4<br>C5	45.720<br>38.261
11	BD (1) C4 -H37	2.00000	98.9679	C4<br>H37	62.175<br>36.844
12	BD (1) C5 -C6	2.00000	98.8017	C5<br>C6	48.786<br>50.225
13	BD (1) C5 -H38	2.00000	99.0192	C5<br>H38	61.671<br>37.398
14	BD (1) C6 -C7	2.00000	98.6778	C6<br>C7	50.464<br>48.420
15	BD (2) C6 -C7	2.00000	80.2026	C3<br>C4	2.908<br>2.351

				C5<br>C6<br>C7<br>C8<br>C9<br>O10	6.832<br>47.611<br>32.679<br>2.955<br>3.070<br>1.548
16	BD (1) C6 -C9	2.00000	98.9945	C6<br>C9	51.778<br>47.318
17	BD (1) C7 -C8	2.00000	98.7867	C7<br>C8	48.996<br>49.968
18	BD (1) C7 -H36	2.00000	98.9718	C7<br>H36	62.825<br>36.202
19	BD (1) C8 -H35	2.00000	98.9327	C8<br>H35	61.764<br>37.223
20	BD (1) C9 -O10	2.00000	99.7766	C9<br>O10	34.102<br>65.687
21	BD (2) C9 -O10	2.00000	98.6982	C9<br>O10	32.829<br>65.870
22	BD (1) C9 -C11	2.00000	98.7822	C9<br>C11	47.028<br>51.792
23	BD (1) C11 -C12	2.00000	99.2023	C11<br>C12	52.187<br>47.047
24	BD (1) C11- H33	2.00000	98.2669	C11<br>H33	62.756<br>35.537
25	BD (1) C11 -H34	2.00000	98.1063	C11<br>H34	61.654<br>36.465
26	BD (1) C12- O13	2.00000	99.7894	C12<br>O13	33.946<br>65.854
27	BD (2) C12 -O13	2.00000	98.6274	C12<br>O13	33.147<br>65.481
28	BD (1) C12- C14	2.00000	99.0305	C11<br>C14	47.304<br>51.824
29	BD (1) C14 -C15	2.00000	98.6626	C14	50.594

				C15	48.274
30	BD (1) C14 -C19	2.00000	98.8071	C14<br>C19	50.325<br>48.696
31	BD (2) C14 -C19	2.00000	81.9209	C14<br>C19	43.766<br>38.158
32	BD (1) C15 -C16	2.00000	98.9611	C15<br>C16	49.339<br>49.799
33	BD (2) C15 -C16	2.00000	82.7772	C15<br>C16	37.622<br>45.193
34	BD (1) C15 -H39	2.00000	99.0164	C15<br>H39	62.687<br>36.387
35	BD (1) C16 -C17	2.00000	98.7424	C16<br>C17	48.720<br>50.237
36	BD (1) C16- H42	2.00000	99.0402	C16<br>H42	61.448<br>37.647
37	BD (1) C17- C18	2.00000	98.7750	C16<br>C17	50.195<br>48.797
38	BD (2) C17- C18	2.00000	80.4822	C17<br>C18	37.356<br>43.138
39	BD (1) C17 -C20	2.00000	98.3700	C17<br>C20	49.633<br>48.874
40	BD (1) C18 -C19	2.00000	98.9213	C18<br>C19	49.526<br>49.577
41	BD (1) C18- H41	2.00000	99.0366	C18<br>H41	61.648<br>37.448
42	BD (1) C19- H40	2.00000	99.0488	C19<br>H40	61.382<br>37.718
43	BD (1) C20 -C21	2.00000	98.4716	C20<br>C21	50.598<br>47.935
44	BD (1) C20 -C22	2.00000	98.4993	C20<br>C22	50.532<br>48.018
45	BD (1) C20- C23	2.00000	98.9394	C20<br>C23	50.558<br>48.440

46	BD (1) C21 -H24	2.00000	99.4982	C21<br>C24	61.801<br>37.713
47	BD (1) C21- H25	2.00000	99.4802	C21<br>H25	61.512<br>37.982
48	BD (1) C21- H26	2.00000	99.4188	C21<br>H36	61.409<br>38.021
49	BD (1) C22- H30	2.00000	99.4220	C22<br>H30	61.433<br>38.000
50	BD (1) C22 -H31	2.00000	99.4824	C22<br>H31	61.496<br>38.000
51	BD (1) C22- H32	2.00000	99.5030	C22<br>H32	61.715<br>37.804
52	BD (1) C23 -H27	2.00000	99.4915	C23<br>H27	61.607<br>37.901
53	BD (1) C23- H28	2.00000	99.4880	C23<br>C28	61.658<br>37.846
54	BD (1) C23 -H29	2.00000	99.4321	C23<br>C29	61.739<br>37.713
55	LP (1) O 2	2.00000	98.1849	O2	98.185
56	LP (2) O 2	2.00000	91.2336	O2	91.234
57	LP (1) O10	2.00000	98.8544	O10	98.854
58	LP (2) O10	2.00000	94.2895	O10	94.290
59	LP (1) O 13	2.00000	98.8758	O13	98.876
60	LP (2) O13	2.00000	94.4746	O13	94.475

Table S7. Octinoxate's significant NLMO occupancy, percentage derived from its parent NBO, and atomic hybrid contributions					
No	Bond	Occupancy	Percentage from parent NBO (%)	Hybrid contributions	
				Atom	Percentage (%)
1	BD (1) O 1- C 8	2.00000	99.4530	O1<br>C8	68.691<br>30.766
2	BD (1) O 1 -C12	2.00000	99.5988	O1<br>C12	68.969<br>30.638
3	BD (1) O 2- C12	2.00000	99.8362	O2<br>C12	65.549<br>34.299
4	BD (2) O 2- C12	2.00000	99.2342	O2<br>C12	69.309<br>29.925
5	BD (1) O 3- C20	2.00000	99.5989	O3<br>C20	67.142<br>32.463
6	BD (1) O 3- C21	2.00000	99.6503	O3<br>C21	67.918<br>31.736
7	BD (1) C 4 -C 5	2.00000	98.7469	C4<br>C5	49.777<br>49.031
8	BD (1) C 4- C 6	2.00000	98.9261	C4<br>C6	50.262<br>48.720
9	BD (1) C 4- C 8	2.00000	98.9802	C4<br>C8	49.773<br>49.247
10	BD (1) C 4 -H22	2.00000	98.5511	C4<br>H22	61.531<br>37.038
11	BD (1) C5- C 7	2.00000	99.2165	C5<br>C7	49.856<br>49.408
12	BD (1) C 5- H23	2.00000	99.0539	C5<br>H23	61.146<br>37.926
13	BD (1) C 5 -H24	2.00000	98.9980	C5<br>H24	61.360<br>37.660
14	BD (1) C 6- C10	2.00000	99.5249	C6<br>C10	50.034<br>49.526
15	BD (1) C 6- H25	2.00000	99.0681	C6<br>H25	61.041<br>38.042

16	BD (1) C 6- H26	2.00000	99.0167	C6<br>H25	61.948<br>37.089
17	BD (1) C 7- C 9	2.00000	99.2235	C7<br>C9	49.931<br>49.336
18	BD (1) C 7 -H27	2.00000	99.0446	C7<br>H27	61.062<br>37.998
19	BD (1) C 7- H28	2.00000	99.0317	C7<br>H28	60.978<br>38.071
20	BD (1) C 8- H29	2.00000	99.3015	C8<br>H29	61.354<br>37.967
21	BD (1) C 8- H30	2.00000	99.2918	C8<br>H30	61.529<br>37.783
22	BD (1) C 9- C11	2.00000	99.5250	C9<br>C11	50.074<br>49.483
23	BD (1) C 9- H31	2.00000	99.0651	C9<br>H31	61.120<br>37.959
24	BD (1) C9- H32	2.00000	99.0680	C9<br>H32	61.060<br>38.022
25	BD (1) C10- H33	2.00000	99.4667	C10<br>H33	61.567<br>37.913
26	BD (1) C10- H34	2.00000	99.5345	C10<br>H34	61.296<br>38.251
27	BD (1) C10- H35	2.00000	99.5300	C10<br>H35	61.122<br>38.422
28	BD (1) C11- H36	2.00000	99.5122	C11<br>H36	61.503<br>38.022
29	BD (1) C11 -H37	2.00000	99.5451	C11<br>H37	61.311<br>38.246
30	BD (1) C11- H38	2.00000	99.5473	C11<br>H38	61.264<br>38.295
31	BD (1) C12 -C13	2.00000	98.8002	C12<br>C13	48.129<br>50.751
32	BD (1) C13- C14	2.00000	99.0739	C13	49.361

				C14	49.887
33	BD (2) C13- C14	2.00000	92.7317	C13<br>C14	50.880<br>41.853
34	BD (1) C13- H39	2.00000	98.9443	C13<br>H39	61.474<br>37.525
35	BD (1) C14- C15	2.00000	98.8289	C14<br>C15	48.409<br>50.591
36	BD (1) C14- H40	2.00000	98.7819	C14<br>H40	62.053<br>36.777
37	BD (1) C15- C16	2.00000	98.7103	C15<br>C16	50.389<br>48.533
38	BD (1) C15 -C17	2.00000	98.7685	C15<br>C17	50.421<br>48.557
39	BD (2) C15 -C17	2.00000	81.5319	C15<br>C17	43.349<br>38.183
40	BD (1) C16 -C18	2.00000	98.8700	C16<br>C18	49.440<br>49.599
41	BD (2) C16- C18	2.00000	84.5093	C16<br>C18	38.853<br>45.685
42	BD (1) C16 -H41	2.00000	99.0289	C16<br>H41	61.582<br>37.497
43	BD (1) C17- C19	2.00000	98.7852	C17<br>C19	49.083<br>49.880
44	BD (1) C17 -H42	2.00000	99.0563	C17<br>H42	61.684<br>37.416
45	BD (1) C18 -C20	2.00000	98.7336	C18<br>C20	48.900<br>49.989
46	BD (1) C18- H43	2.00000	98.9629	C18<br>H43	62.122<br>36.892
47	BD (1) C19 -C20	2.00000	99.0349	C19<br>C20	49.517<br>49.680
48	BD (2) C19- C20	2.00000	80.8158	O1<br>O2	0.017<br>0.052

				O3<br>C12<br>C13<br>C14<br>C15<br>C16<br>C17<br>C18<br>C19<br>C20<br>C21	0.045<br>0.121<br>0.381<br>0.658<br>4.333<br>3.518<br>6.333<br>3.541<br>45.704<br>35.148<br>0.055
49	BD (1) C19- H44	2.00000	98.9275%	C19<br>H44	61.689<br>37.296
50	BD (1) C21- H45	2.00000	99.5451	C21<br>H45	61.545<br>38.004
51	BD (1) C21 -H46	2.00000	99.7756	C21<br>H46	60.894<br>38.892
52	BD (1) C21-H47	2.00000	99.7756	C21<br>H47	60.891<br>38.895
53	LP (1) O 1	2.00000	98.1597	O1	98.160
54	LP (2) O 1	2.00000	89.1463	O1	89.146
55	LP (1) O 2	2.00000	98.8205	O2	98.821
56	LP (2) O 2	2.00000	92.1420	O2	92.142
57	LP (1) O 3	2.00000	98.1803	O3	98.181
58	LP (2) O 3	2.00000	91.4526	O3	91.453

**Table S8.** Padimate O's significant NLMO occupancy, percentage derived from its parent NBO, and atomic hybrid contributions					
No	Bond	Occupancy	Percentage from parent NBO (%)	Hybrid contributions	
				Atom	Percentage (%)
1	BD (1) O 1- C 8	2.00000	99.4396	O1<br>C8	68.757<br>30.687
2	BD (1) O 1 -C12	2.00000	99.5793	O1<br>C12	69.000<br>30.584
3	BD (1) O 2-C12	2.00000	99.8161	O2<br>C12	65.544<br>34.284
4	BD (2) O 2- C12	2.00000	99.1861	O2<br>C12	69.247<br>29.939
5	BD (1) N 3 -C14	2.00000	99.2896	N3<br>C14	60.803<br>38.579
6	BD (1) N 3- C19	2.00000	99.4341	N3<br>C19	61.776<br>37.681
7	BD (1) N 3 -C20	2.00000	99.4348	N3<br>C20	61.754<br>37.703
8	BD (1) C 4 -C 5	2.00000	98.7517	C4<br>C5	49.765<br>49.048
9	BD (1) C 4- C 6	2.00000	98.9244	C4<br>C6	50.235<br>48.745
10	BD (1) C 4 -C 8	2.00000	98.9746	C4<br>C8	49.831<br>49.183
11	BD (1) C 4- H21	2.00000	98.5571	C4<br>C21	61.502<br>37.073
12	BD (1) C 5 -C 7	2.00000	99.2162	C5<br>C7	49.851<br>49.413
13	BD (1) C 5- H22	2.00000	99.0519	C5<br>H22	61.155<br>37.916
14	BD (1) C 5- H23	2.00000	98.9984	C5<br>H23	61.338<br>37.682
15	BD (1) C 6 -C10	2.00000	99.5262	C6<br>C10	50.007<br>49.554

16	BD (1) C 6- H24	2.00000	99.0660	C6<br>H24	61.057<br>38.024
17	BD (1) C 6- H25	2.00000	99.0176	C6<br>H25	61.929<br>37.109
18	BD (1) C 7- C 9	2.00000	99.2230	C7<br>C9	49.919<br>49.348
19	BD (1) C 7 -H26	2.00000	99.0458	C7<br>H26	61.051<br>38.010
20	BD (1) C7- H27	2.00000	99.0331	C7<br>H27	60.993<br>38.058
21	BD (1) C 8- H28	2.00000	99.3210	C8<br>H28	61.448<br>37.892
22	BD (1) C 8- H29	2.00000	99.2984	C8<br>H29	61.458<br>37.861
23	BD (1) C 9- C11	2.00000	99.5259	C9<br>C11	50.067<br>49.490
24	BD (1) C 9- H30	2.00000	99.0652	C9<br>H30	61.122<br>37.957
25	BD (1) C 9 -H31	2.00000	99.0682	C9<br>H31	61.051<br>61.051
26	BD (1) C10- H32	2.00000	99.4678	C10<br>H32	61.557<br>37.924
27	BD (1) C10- H33	2.00000	99.5357	C10<br>H33	61.265<br>38.283
28	BD (1) C10 -H34	2.00000	99.5296	C10<br>H34	61.124<br>38.419
29	BD (1) C11- H35	2.00000	99.5127	C11<br>H35	61.498<br>38.028
30	BD (1) C11- H36	2.00000	99.5449	C11<br>H36	61.313<br>38.244
31	BD (1) C11- H37	2.00000	99.5475	C11<br>H37	61.259<br>38.301
32	BD (1) C12 -C13	2.00000	98.7184	C12	47.204

				C13	51.631
33	BD (1) C13- C15	2.00000	98.7628	C13<br>C15	50.435<br>48.544
34	BD (2) C13 -C15	2.00000	81.1806	C13<br>C15	48.880<br>32.431
35	BD (1) C13 -C16	2.00000	98.6373	C13<br>C16	50.423<br>48.423
36	BD (1) C14- C17	2.00000	98.6719	C14<br>C17	50.603<br>48.273
37	BD (2) C14- C17	2.00000	78.9123	O1<br>O2<br>N3<br>C12<br>C13<br>C14<br>C15<br>C16<br>C17<br>C18<br>C19<br>C20	0.081<br>0.254<br>0.103<br>0.587<br>2.849<br>30.973<br>10.586<br>3.013<br>48.167<br>3.156<br>0.033<br>0.048
38	BD (1) C14 -C18	2.00000	98.6701	C14<br>C18	50.644<br>48.234
39	BD (1) C15- C17	2.00000	98.8907	C15<br>C17	49.278<br>49.798
40	BD (1) C15- H38	2.00000	99.0039	C15<br>H38	62.216<br>36.840
41	BD (1) C16- C18	2.00000	98.8960	C16<br>C18	49.198<br>49.879
42	BD (2) C16- C18	2.00000	85.3406	C16<br>C18	36.984<br>48.376
43	BD (1) C16- H39	2.00000	98.9777	C16<br>H39	62.525<br>36.508

44	BD (1) C17 -H40	2.00000	98.9846	C17<br>H40	61.418<br>37.625
45	BD (1) C18- H41	2.00000	98.9820	C18<br>H41	61.461<br>37.578
46	BD (1) C19- H42	2.00000	99.5198	C19<br>H42	61.665<br>37.863
47	BD (1) C19- H43	2.00000	99.5216	C19<br>H43	61.554<br>37.976
48	BD (1) C19 -H44	2.00000	99.5892	C19<br>H44	61.550<br>38.045
49	BD (1) C20 -H45	2.00000	99.5222	C20<br>H45	61.564<br>37.966
50	BD (1) C20- H46	2.00000	99.5219	C20<br>H46	61.633<br>37.897
51	BD (1) C20- H47	2.00000	99.5881	C20<br>H47	61.538<br>38.055
52	LP (1) O 1	2.00000	98.0790	O1	98.080
53	LP (2) O 1	2.00000	89.6562	O1	89.656
54	LP (1) O 2	2.00000	98.8295	O2	98.830
55	LP (2) O 2	2.00000	92.3337	O2	92.334
56	LP (1) N3	2.00000	84.5573	N3	84.559
